# Supplementary figures and images for: Commissureless acts as a substrate adapter in a conserved Nedd4 E3 ubiquitin ligase pathway to promote axon growth across the midline
Source: eLife. 2025 May 23;13:RP92757. doi: 10.7554/eLife.92757 (PMC12101832; doi:10.7554/eLife.92757)

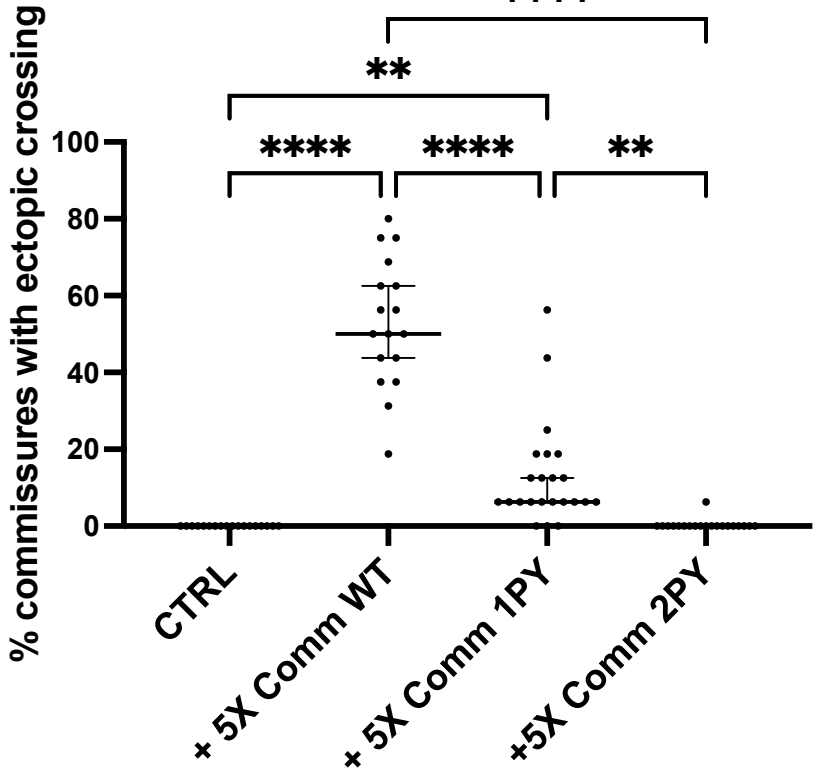

Supplement: Figure 1—source data 3. [file elife-92757-fig1-data3.pdf]

% segments with ectopic crossing

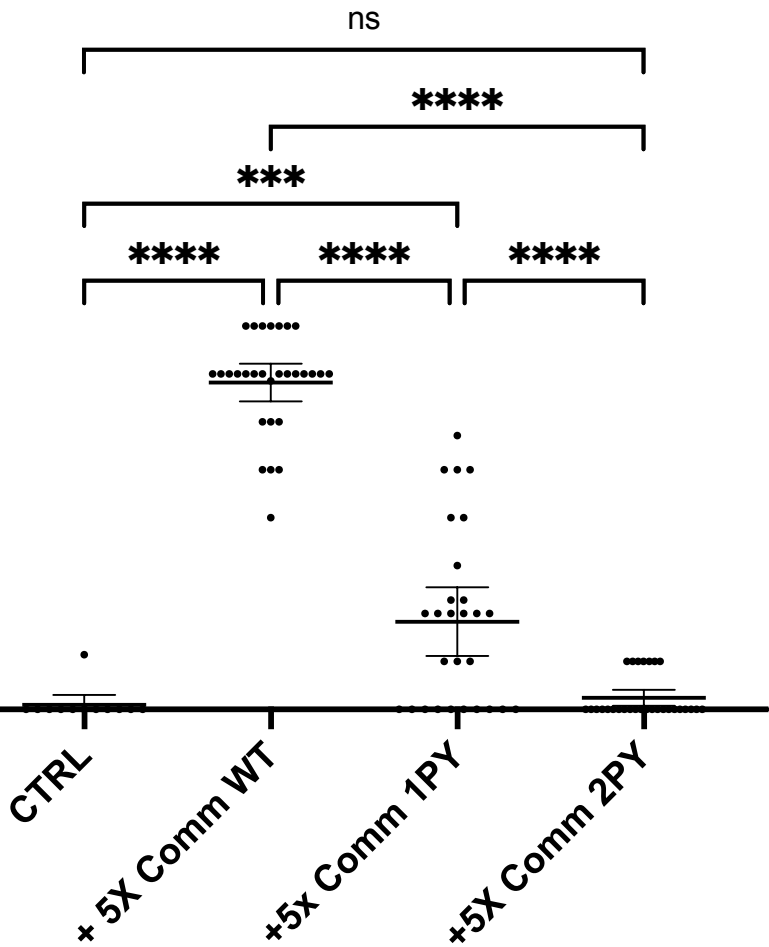

Supplement: Figure 1—source data 6. [file elife-92757-fig1-data6.pdf]

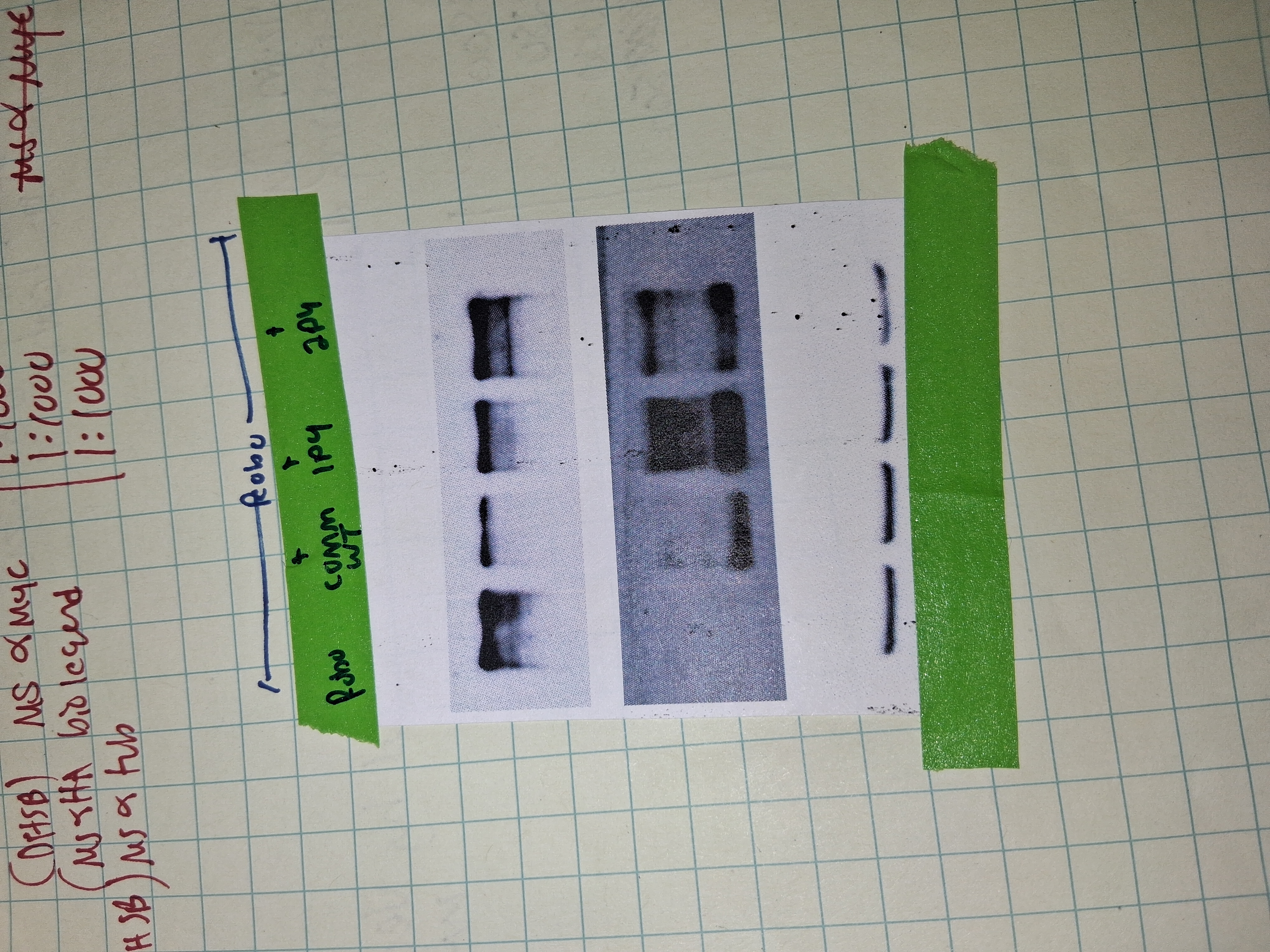

Supplement: Figure 2—source data 1. [file elife-92757-fig2-data1.zip › Fig 2 A-C source data 1/WB 1.jpg]

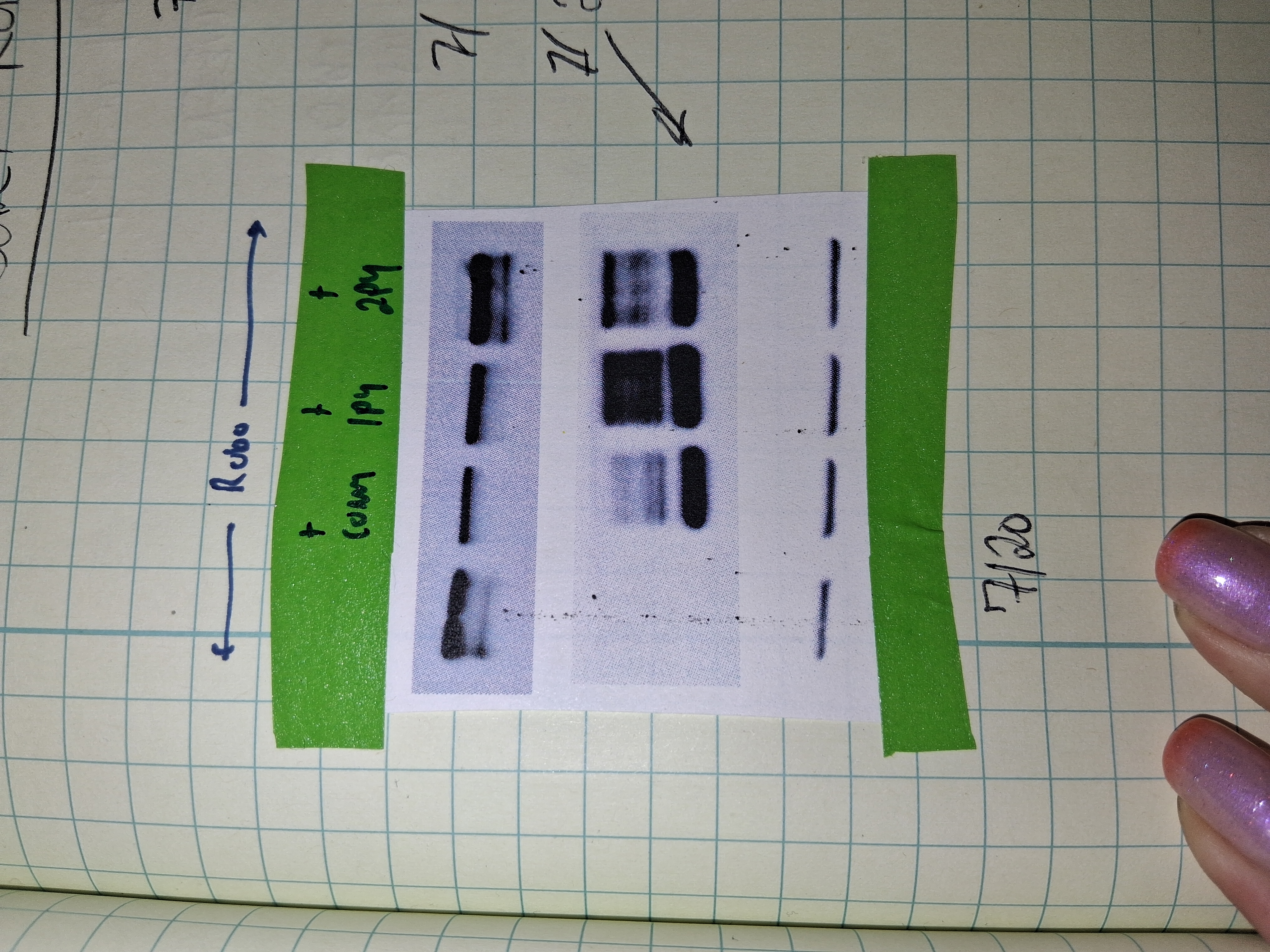

Supplement: Figure 2—source data 1. [file elife-92757-fig2-data1.zip › Fig 2 A-C source data 1/WB 2.jpg]

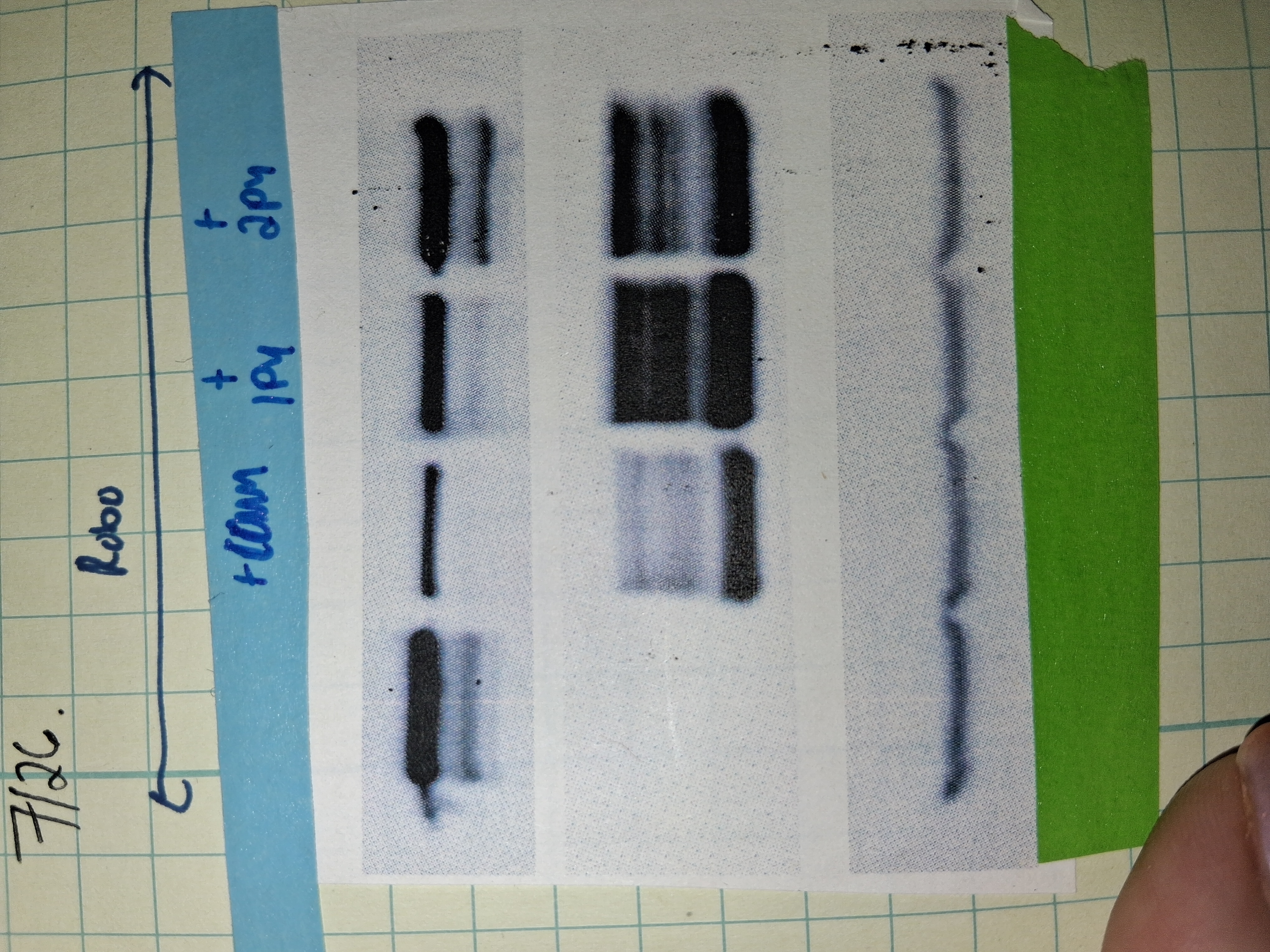

Supplement: Figure 2—source data 1. [file elife-92757-fig2-data1.zip › Fig 2 A-C source data 1/WB 3.jpg]

Western blot 1, Jul 13

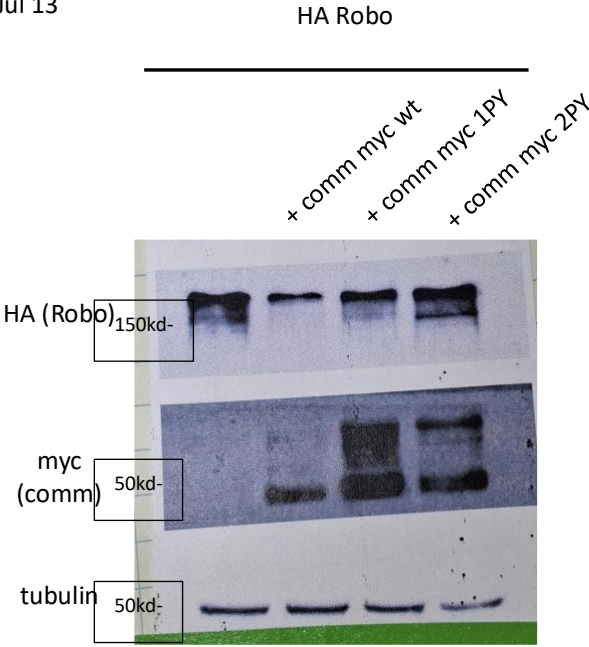

Jul 20 Western blot 2

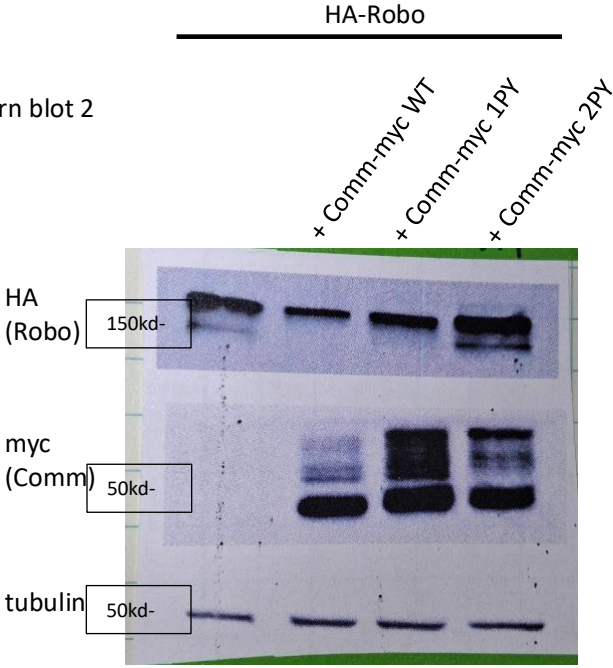

Jul 26 Western blot 3

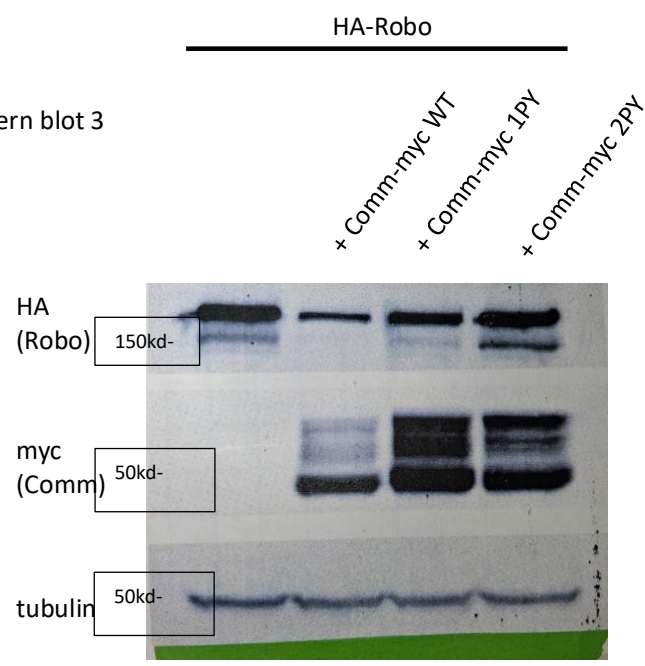

Supplement: Figure 2—source data 2. [file elife-92757-fig2-data2.zip › Fig 2 A-C source data 2/Fig 2 A-C Robo levels western blot.pdf]

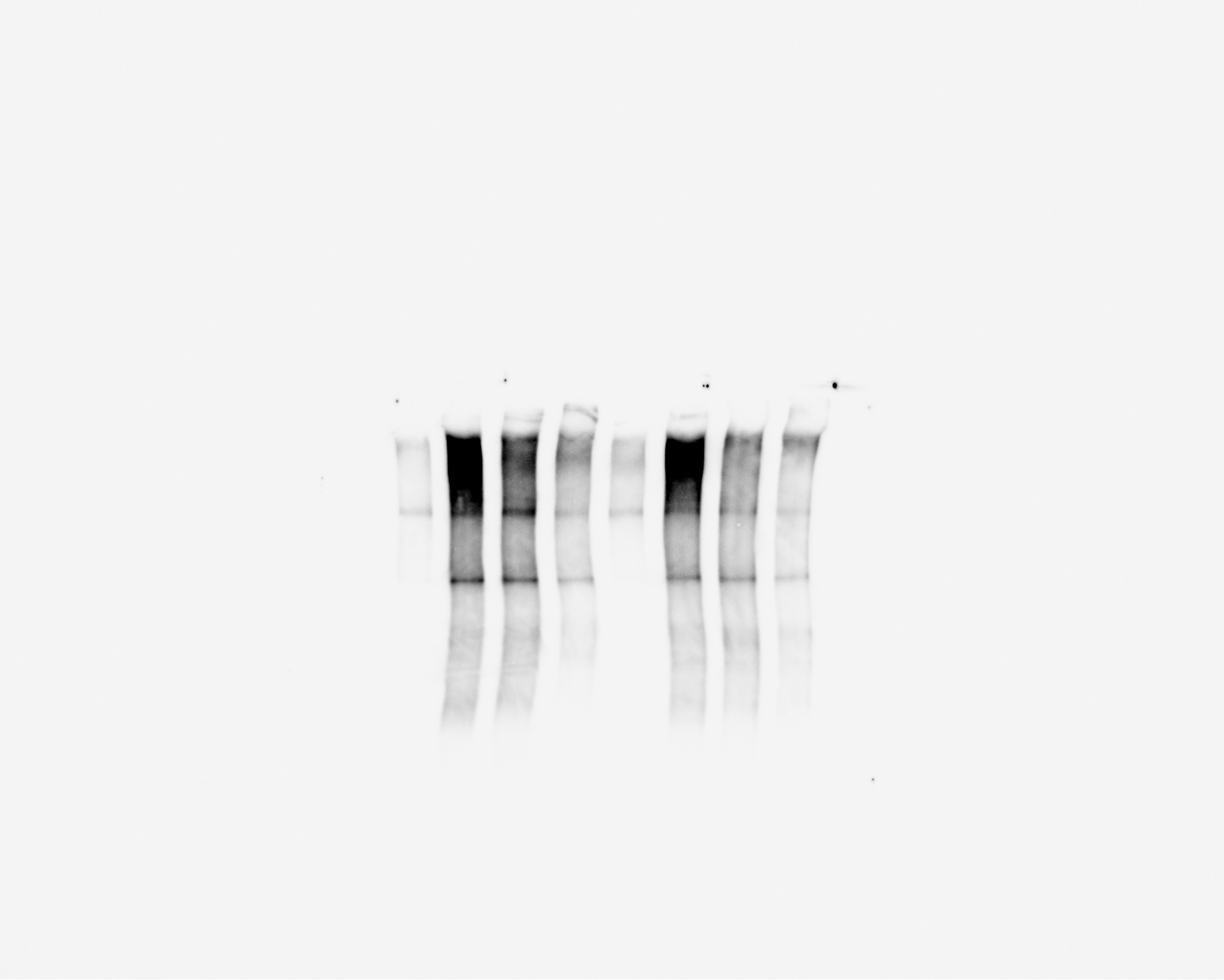

Supplement: Figure 2—source data 3. [file elife-92757-fig2-data3.zip › figure 2 D-E source data 1/10-12 blot 4/ubiquitinated robo ip.tif]

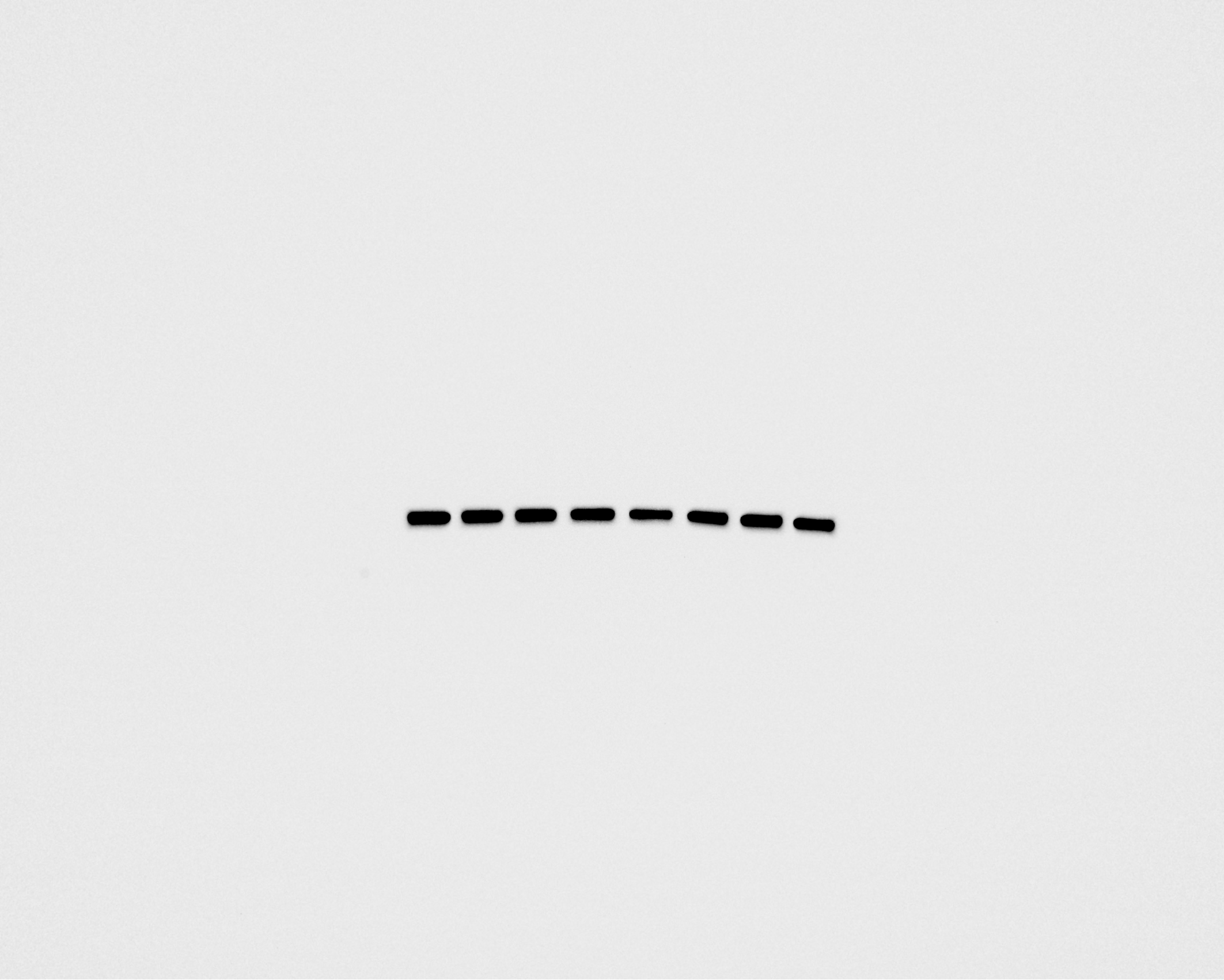

Supplement: Figure 2—source data 3. [file elife-92757-fig2-data3.zip › figure 2 D-E source data 1/10-12 blot 4/tubulin.tif]

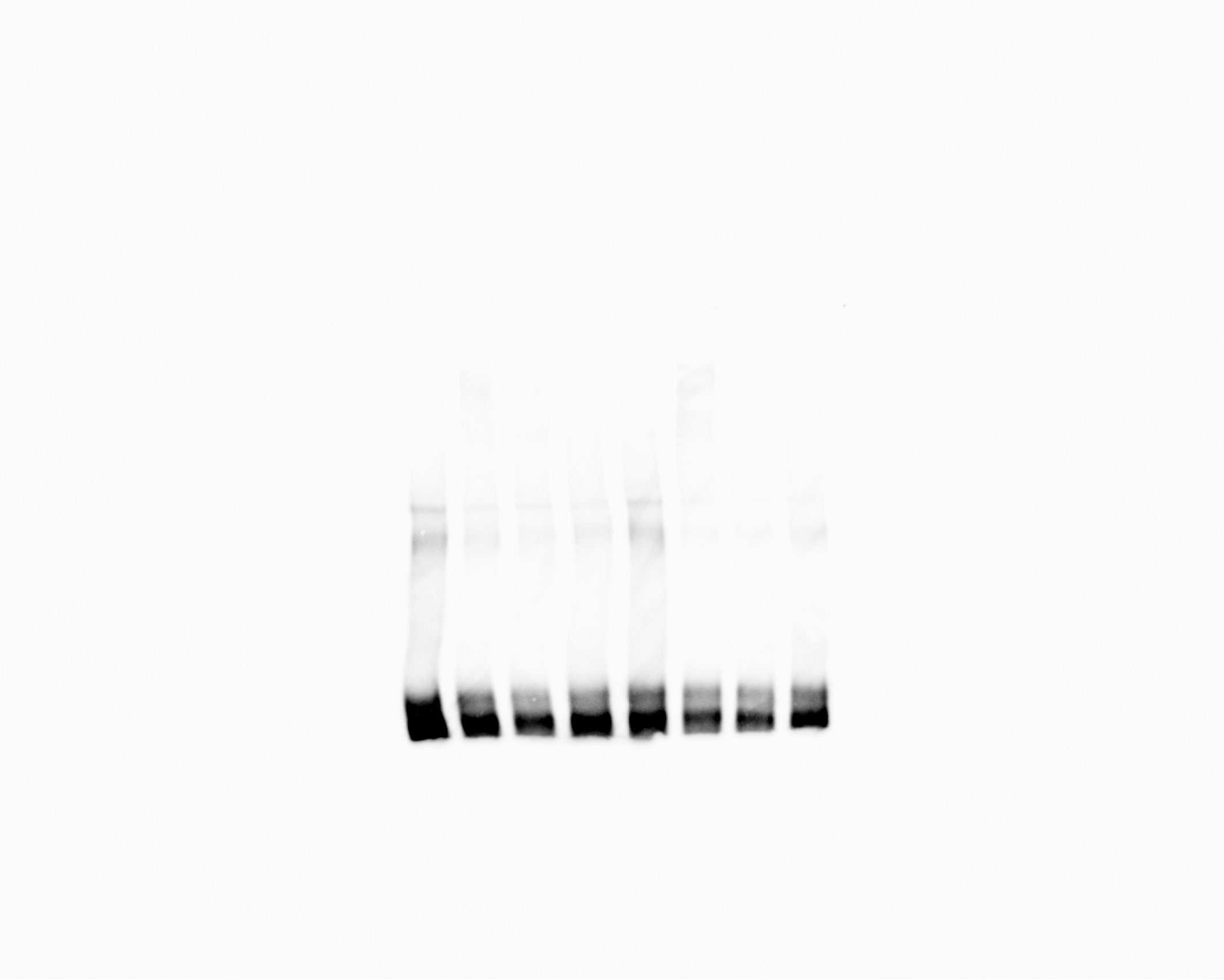

Supplement: Figure 2—source data 3. [file elife-92757-fig2-data3.zip › figure 2 D-E source data 1/10-12 blot 4/igg.tif]

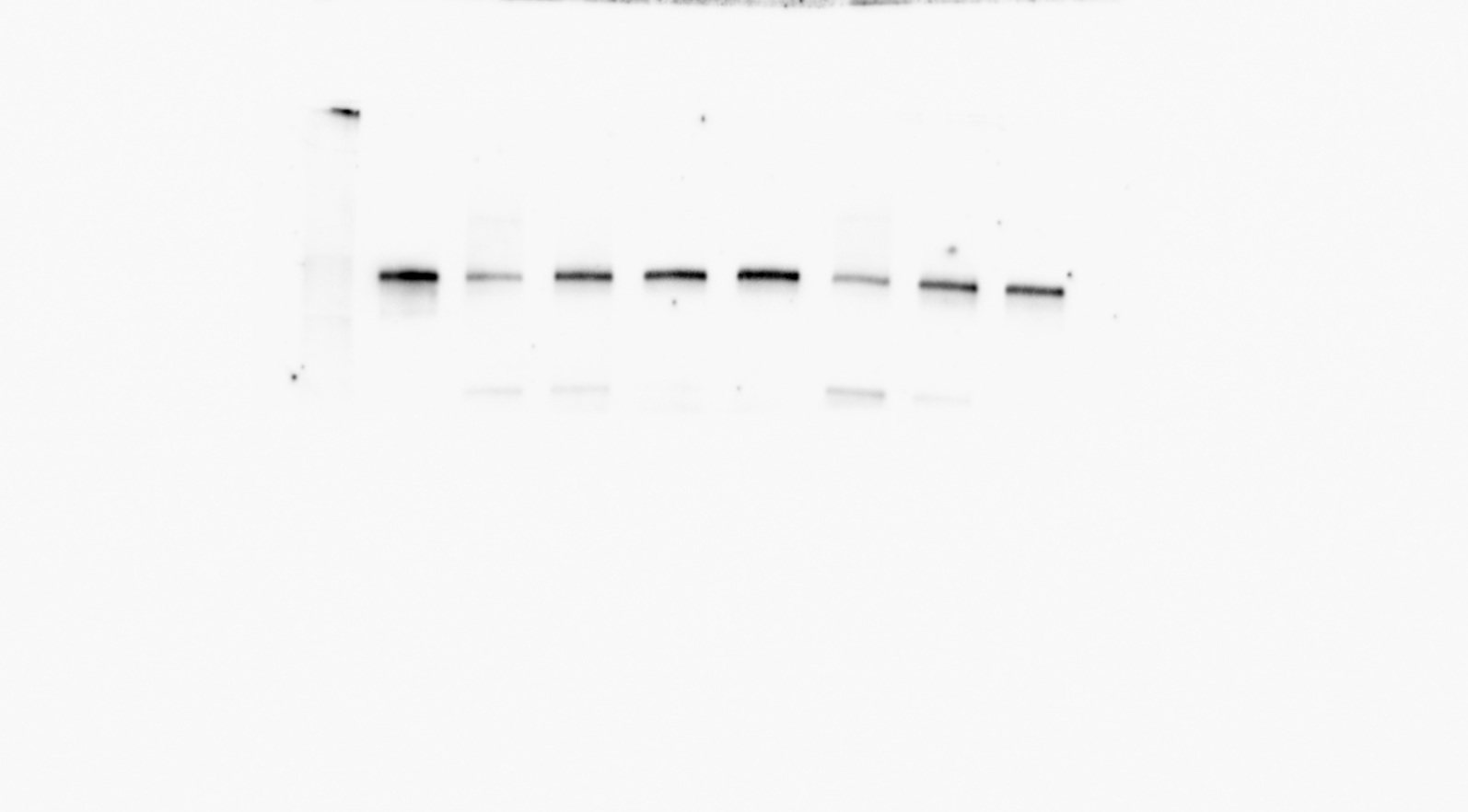

Supplement: Figure 2—source data 3. [file elife-92757-fig2-data3.zip › figure 2 D-E source data 1/10-12 blot 4/robo lysate.tif]

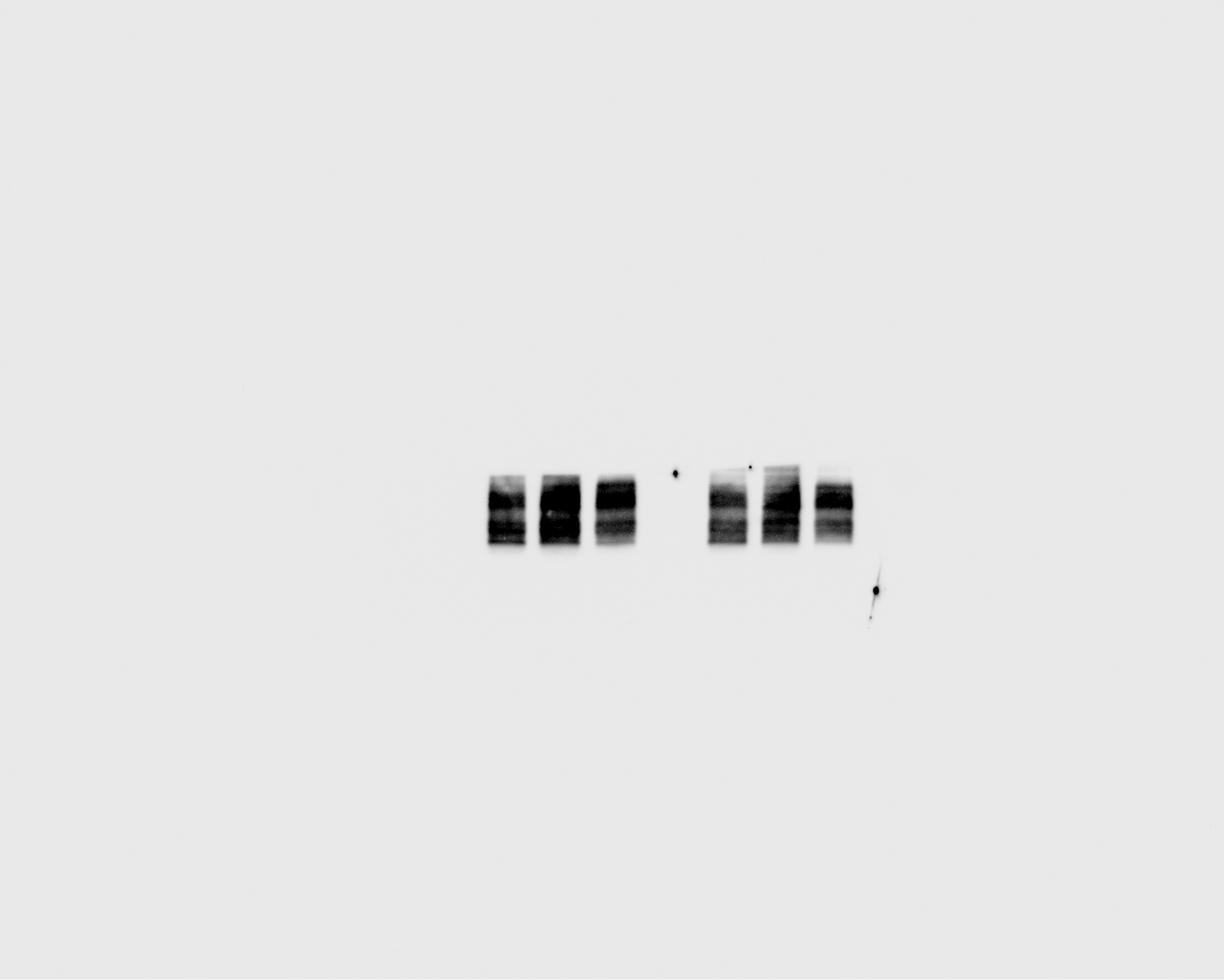

Supplement: Figure 2—source data 3. [file elife-92757-fig2-data3.zip › figure 2 D-E source data 1/10-12 blot 4/comm lysate.tif]

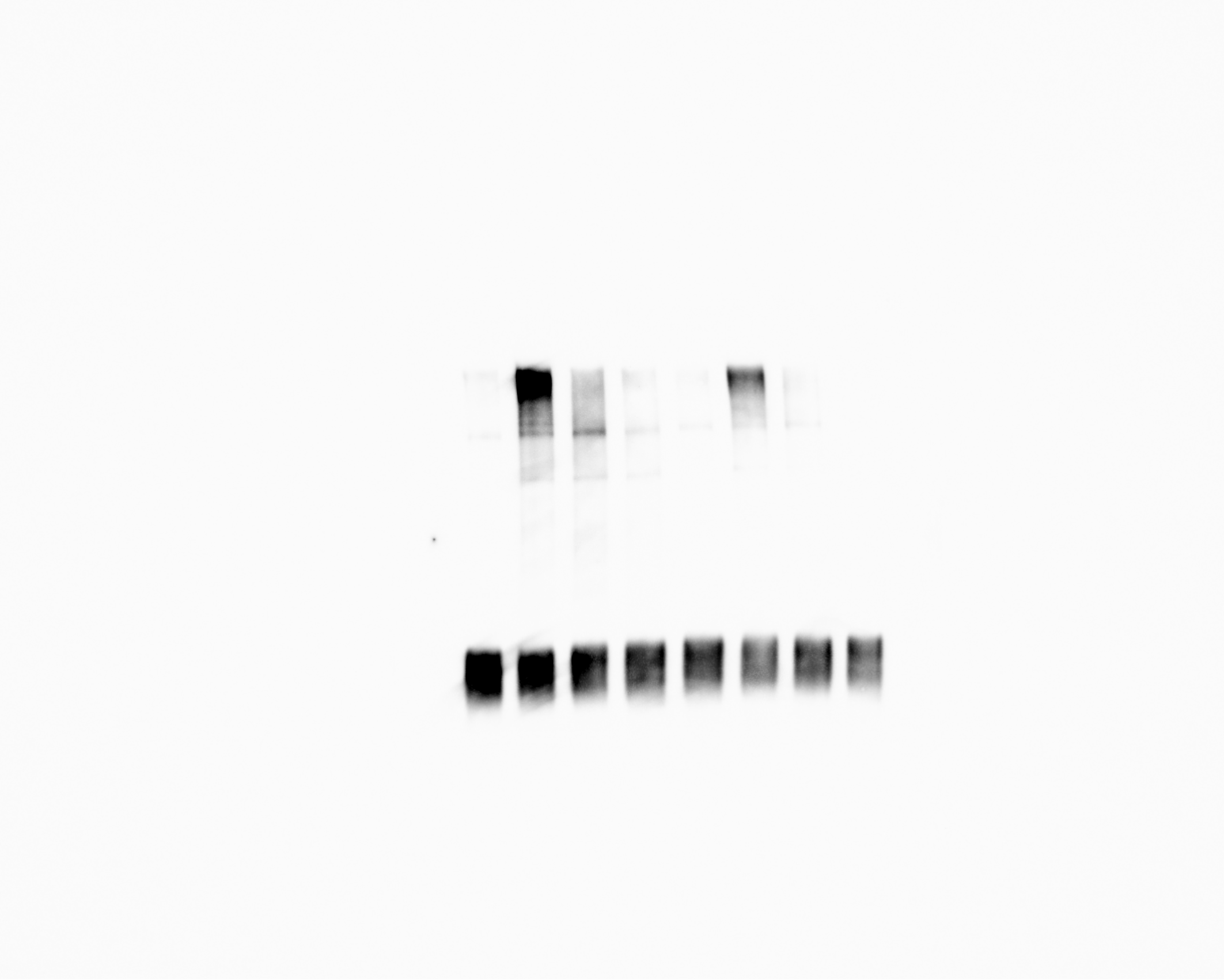

Supplement: Figure 2—source data 3. [file elife-92757-fig2-data3.zip › figure 2 D-E source data 1/10-3 blot 3/igg.tif]

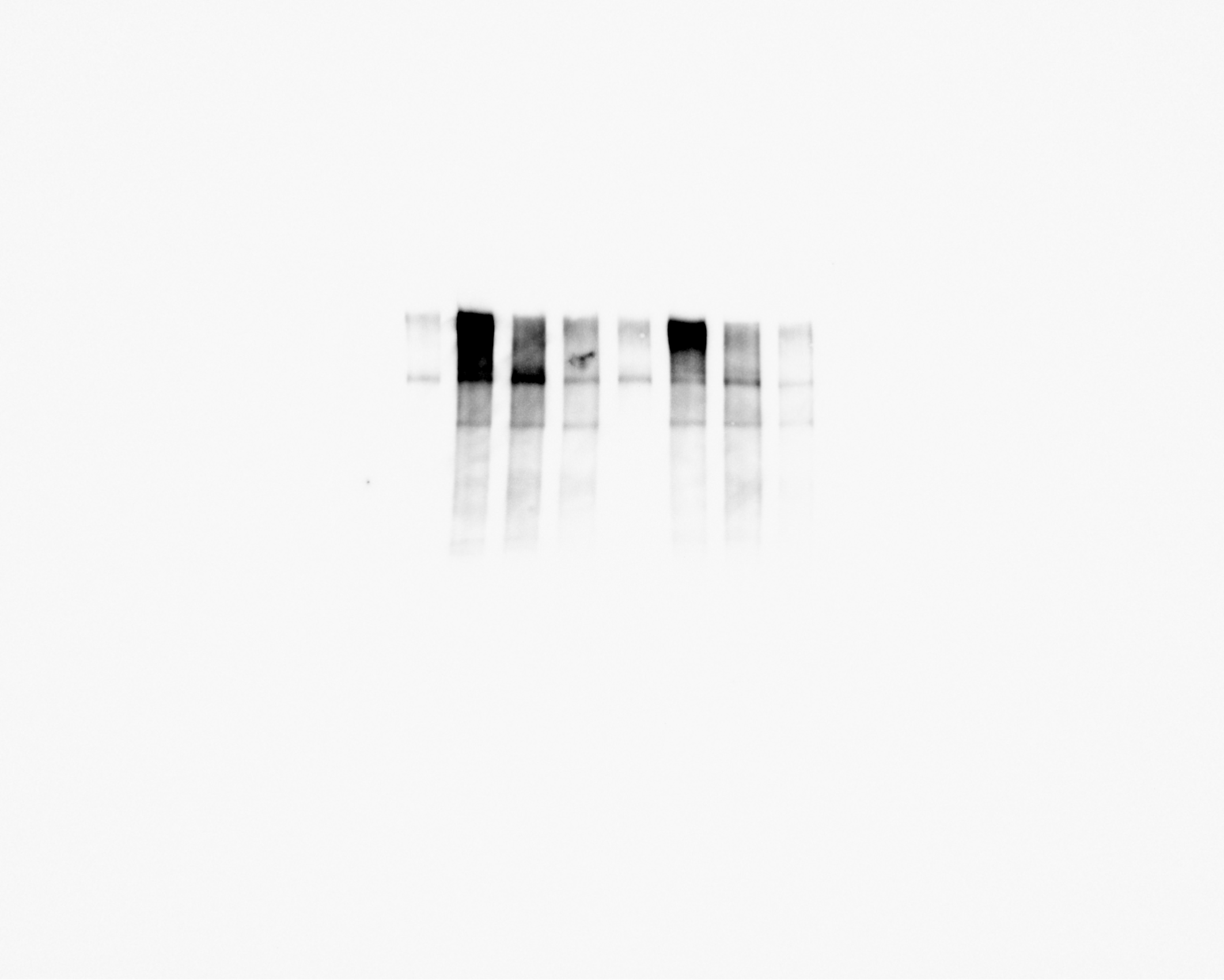

Supplement: Figure 2—source data 3. [file elife-92757-fig2-data3.zip › figure 2 D-E source data 1/10-3 blot 3/ubiquitinated robo .tif]

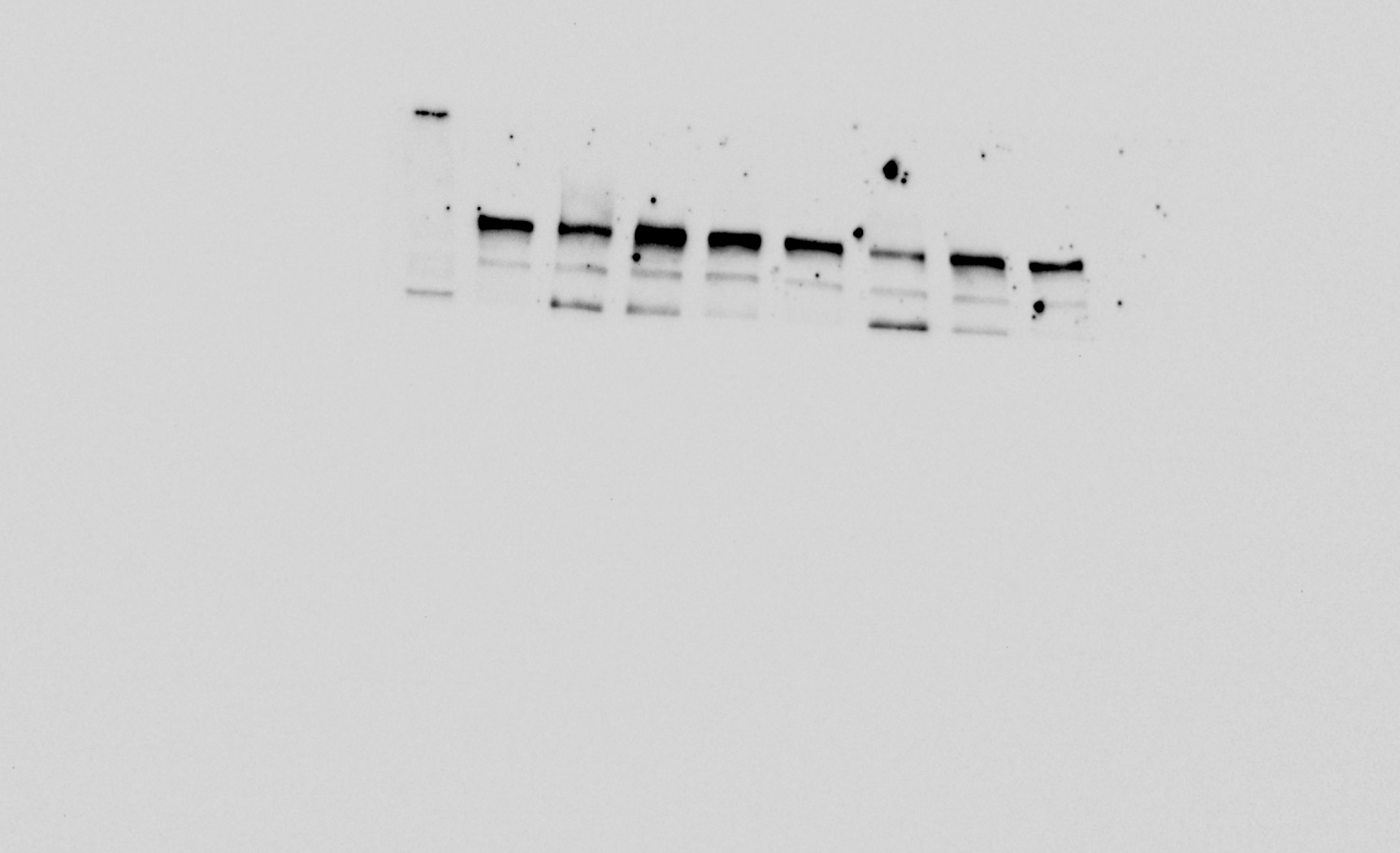

Supplement: Figure 2—source data 3. [file elife-92757-fig2-data3.zip › figure 2 D-E source data 1/10-3 blot 3/robo lysate.tif]

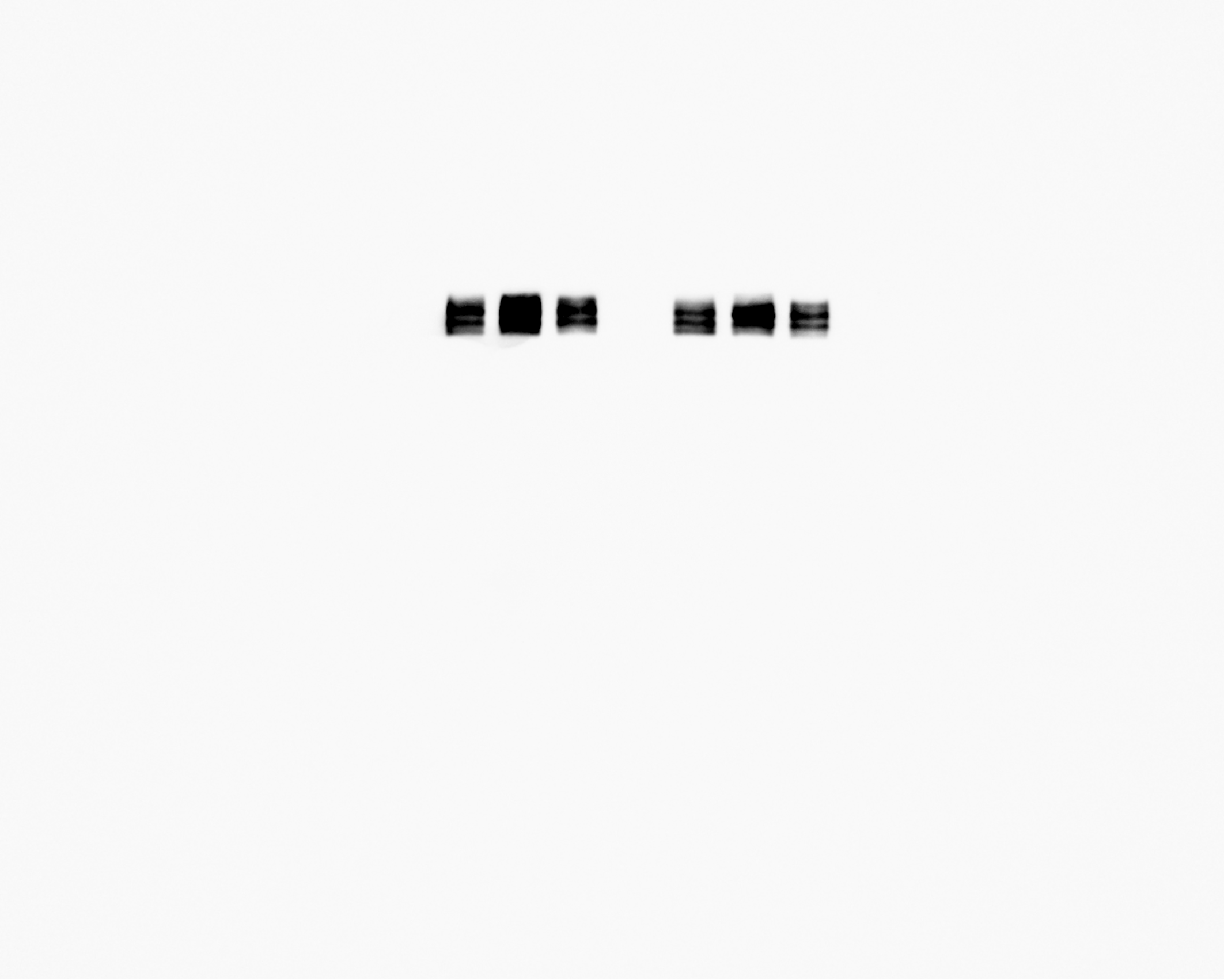

Supplement: Figure 2—source data 3. [file elife-92757-fig2-data3.zip › figure 2 D-E source data 1/10-3 blot 3/comm lysate.tif]

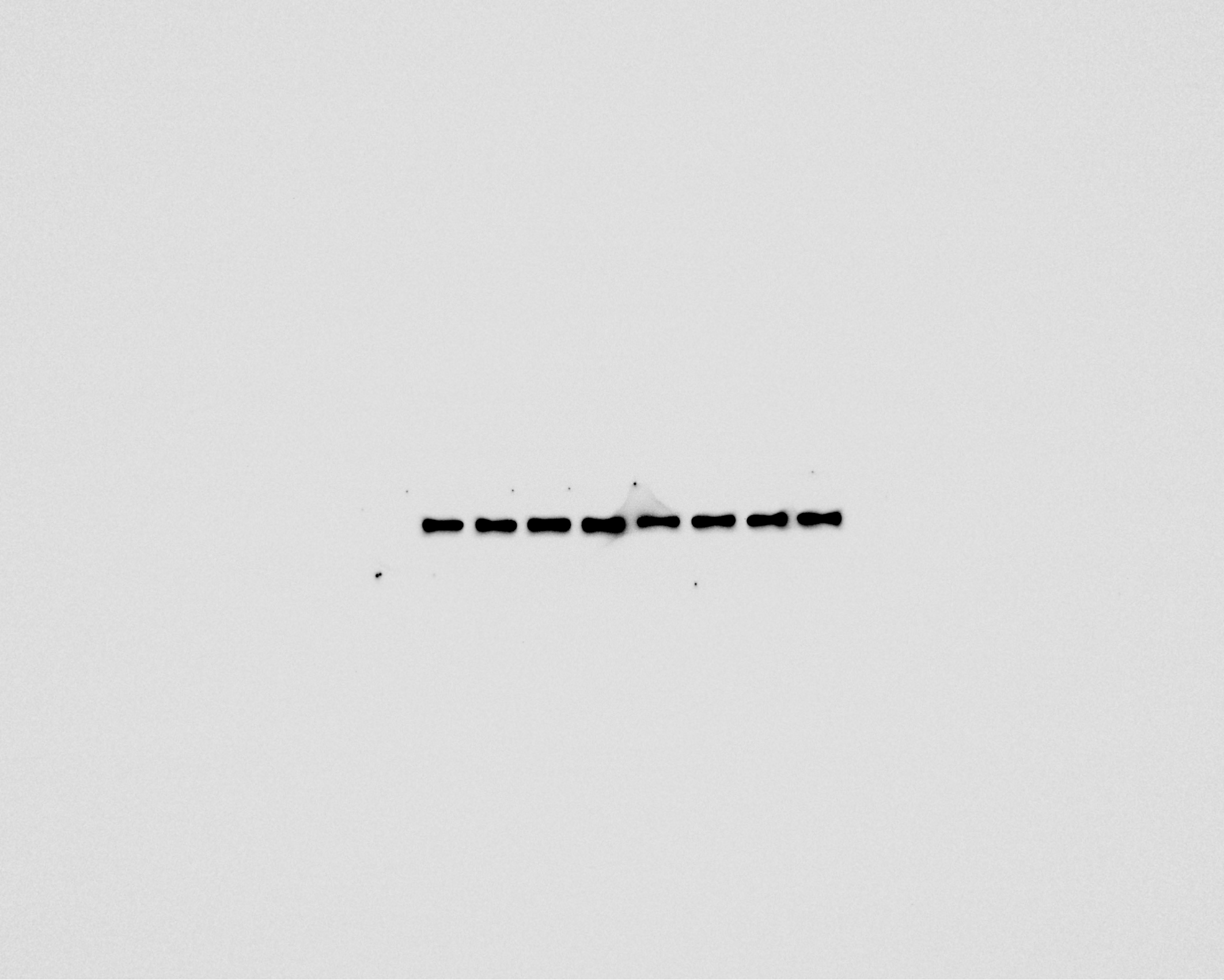

Supplement: Figure 2—source data 3. [file elife-92757-fig2-data3.zip › figure 2 D-E source data 1/10-3 blot 3/tubulin lysate.tif]

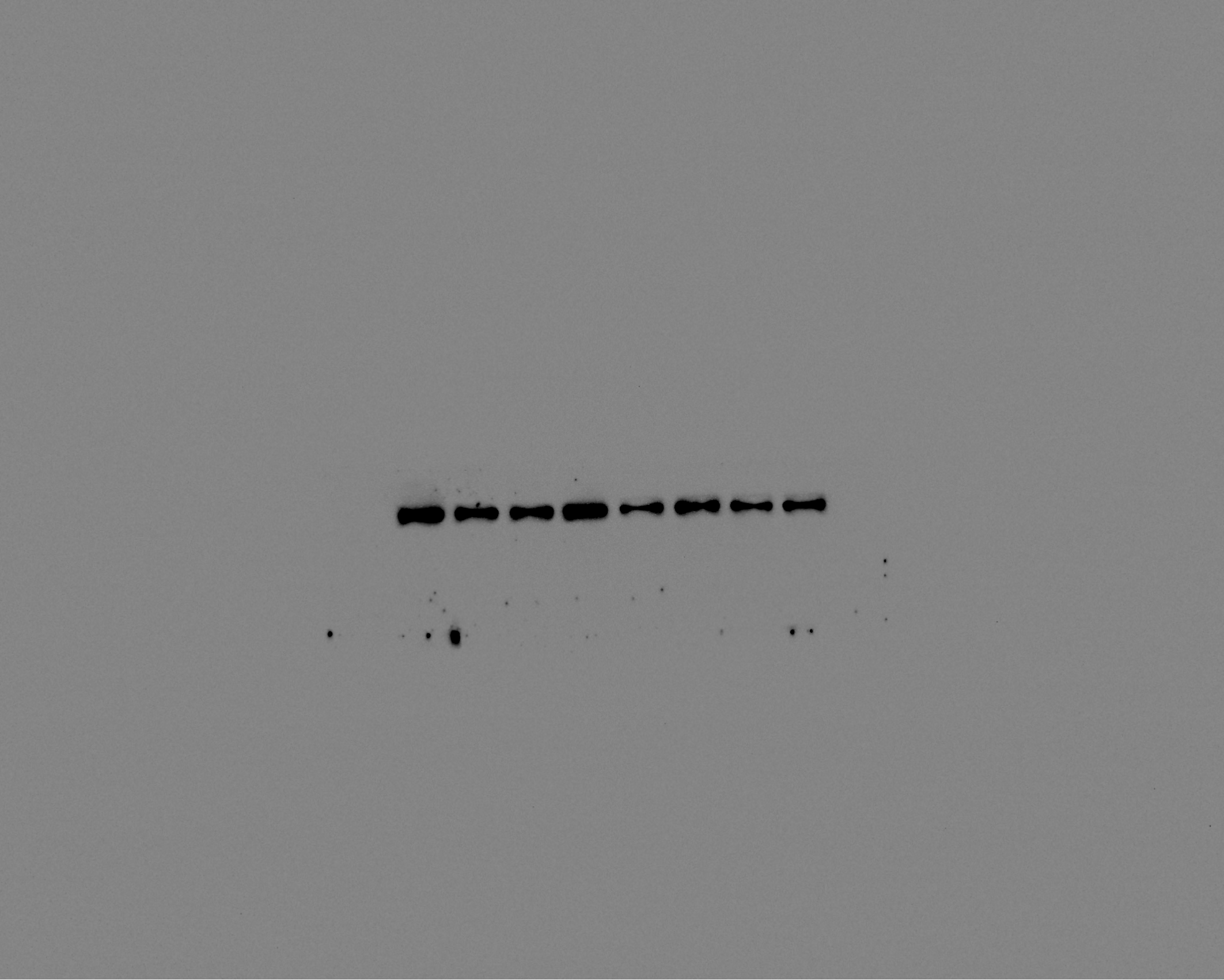

Supplement: Figure 2—source data 3. [file elife-92757-fig2-data3.zip › figure 2 D-E source data 1/8-25 blot 2/tubulin.tif]

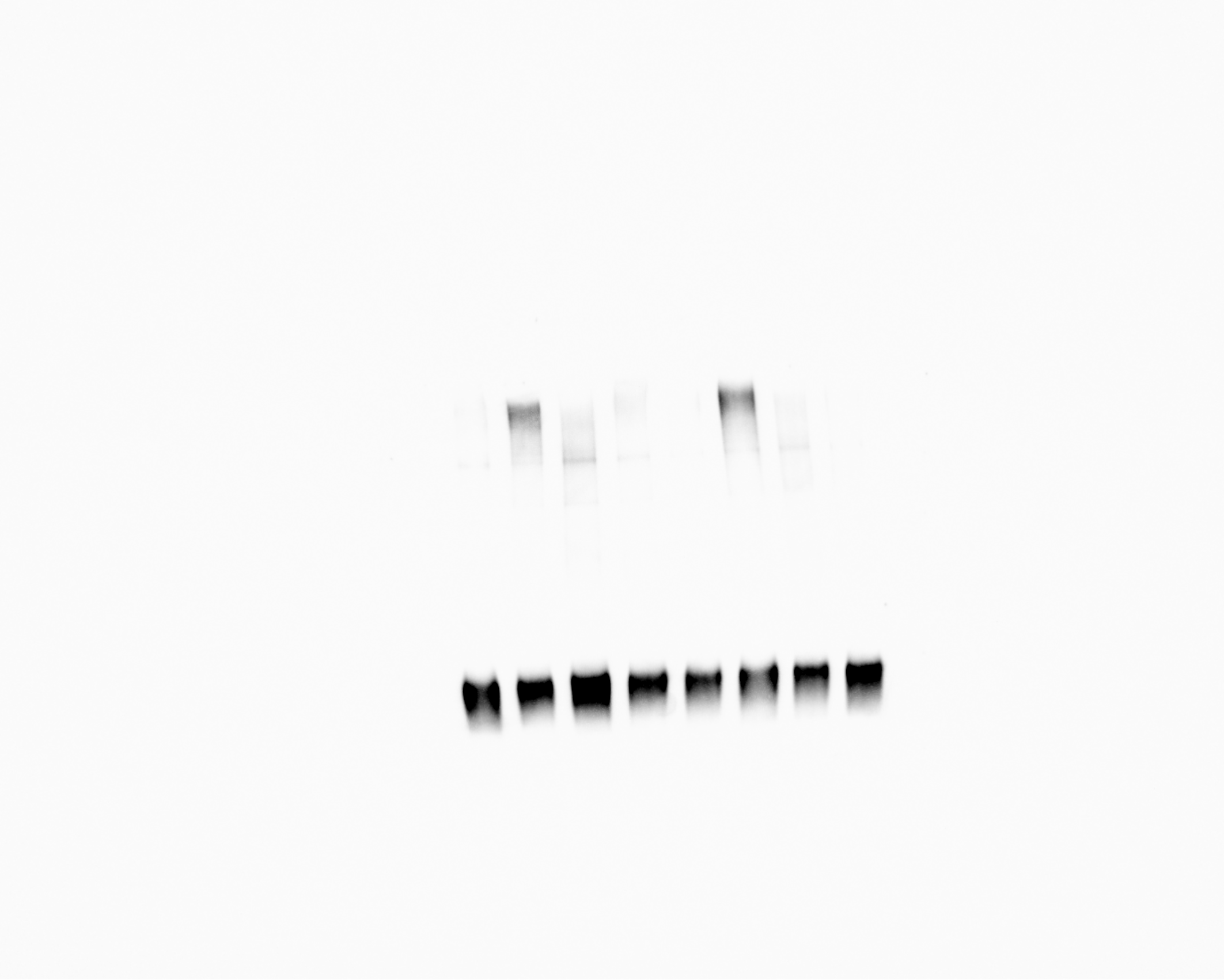

Supplement: Figure 2—source data 3. [file elife-92757-fig2-data3.zip › figure 2 D-E source data 1/8-25 blot 2/ubiquitinated robo ip and igg.tif]

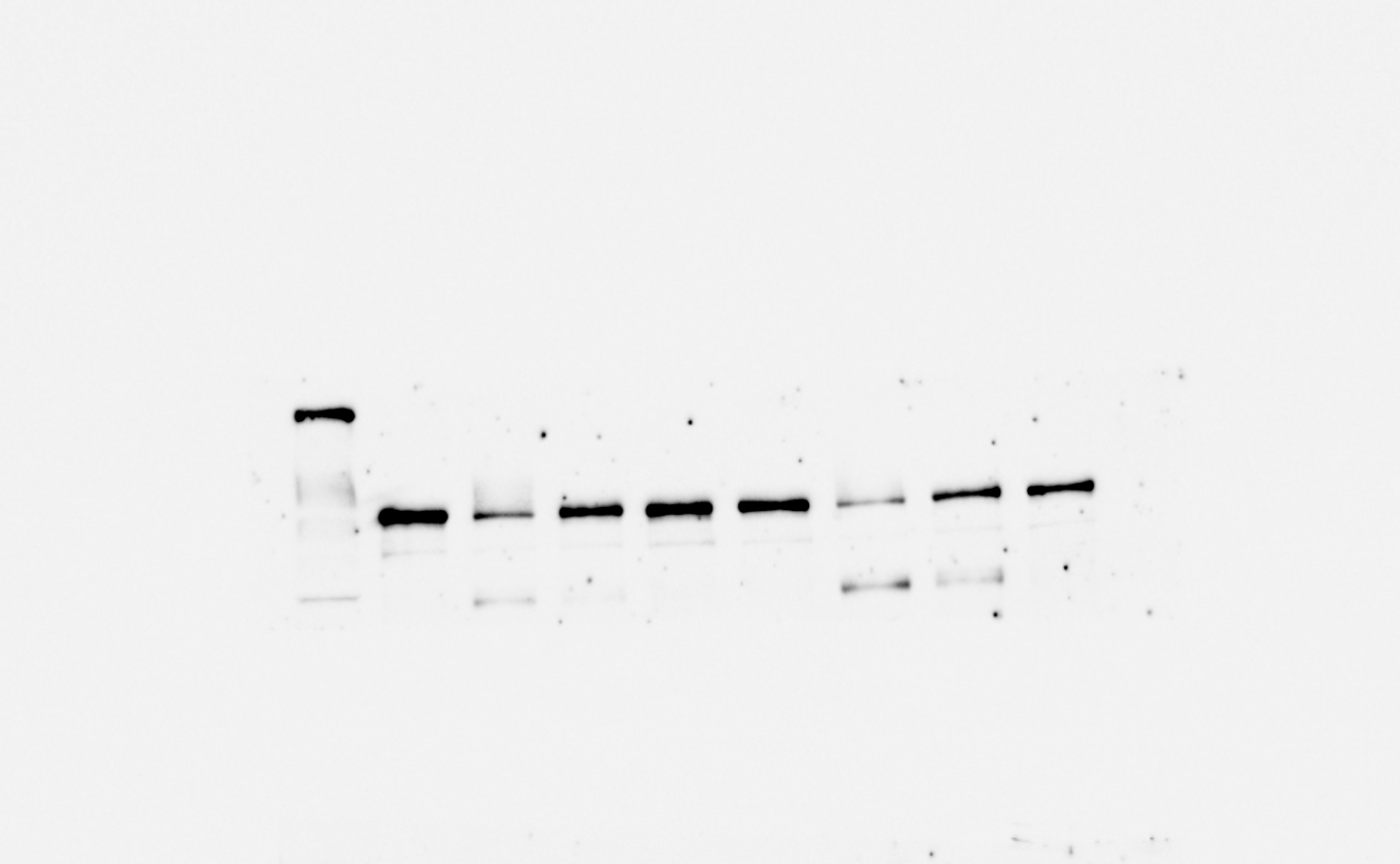

Supplement: Figure 2—source data 3. [file elife-92757-fig2-data3.zip › figure 2 D-E source data 1/8-25 blot 2/robo lysate.tif]

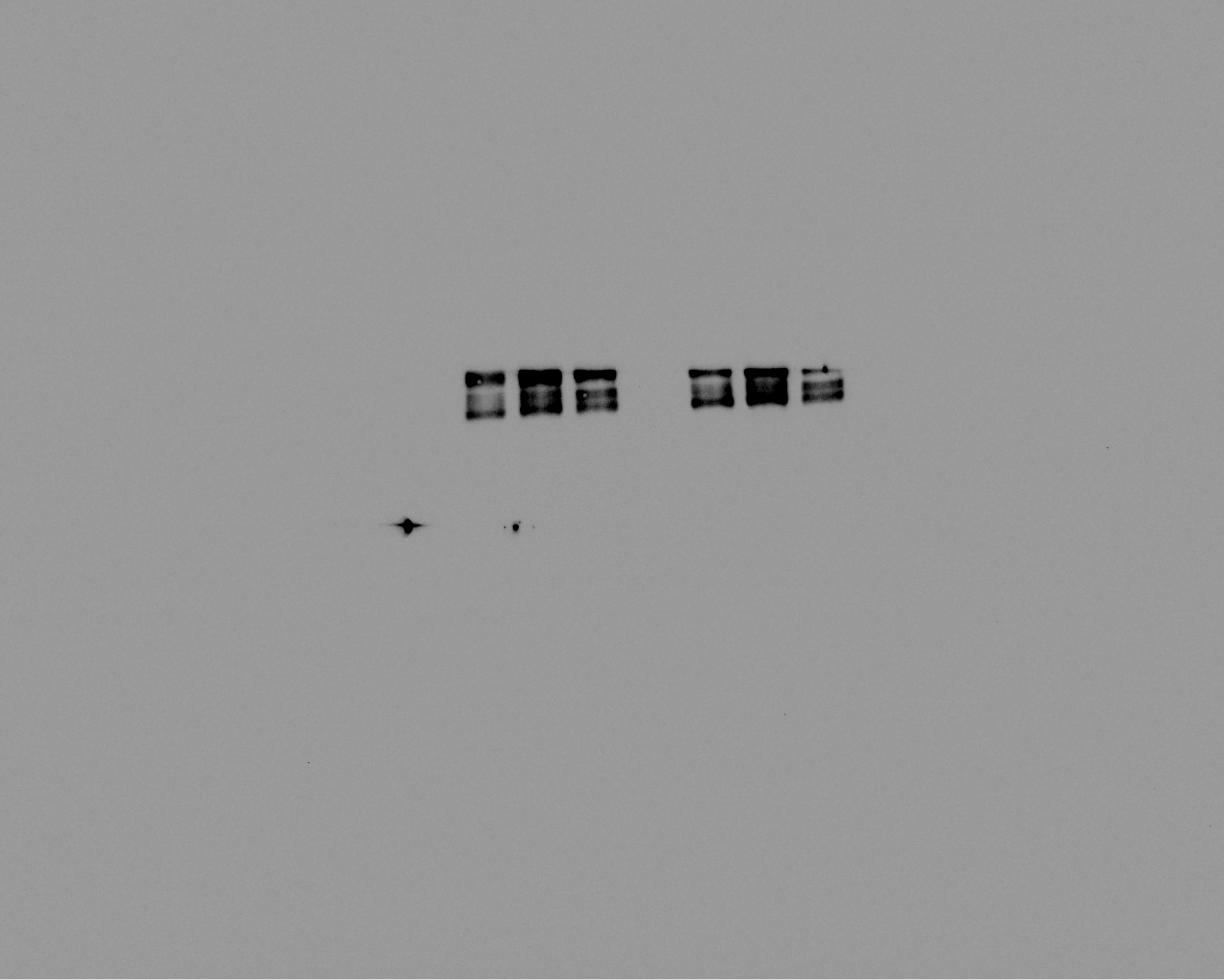

Supplement: Figure 2—source data 3. [file elife-92757-fig2-data3.zip › figure 2 D-E source data 1/8-25 blot 2/comm lysate.tif]

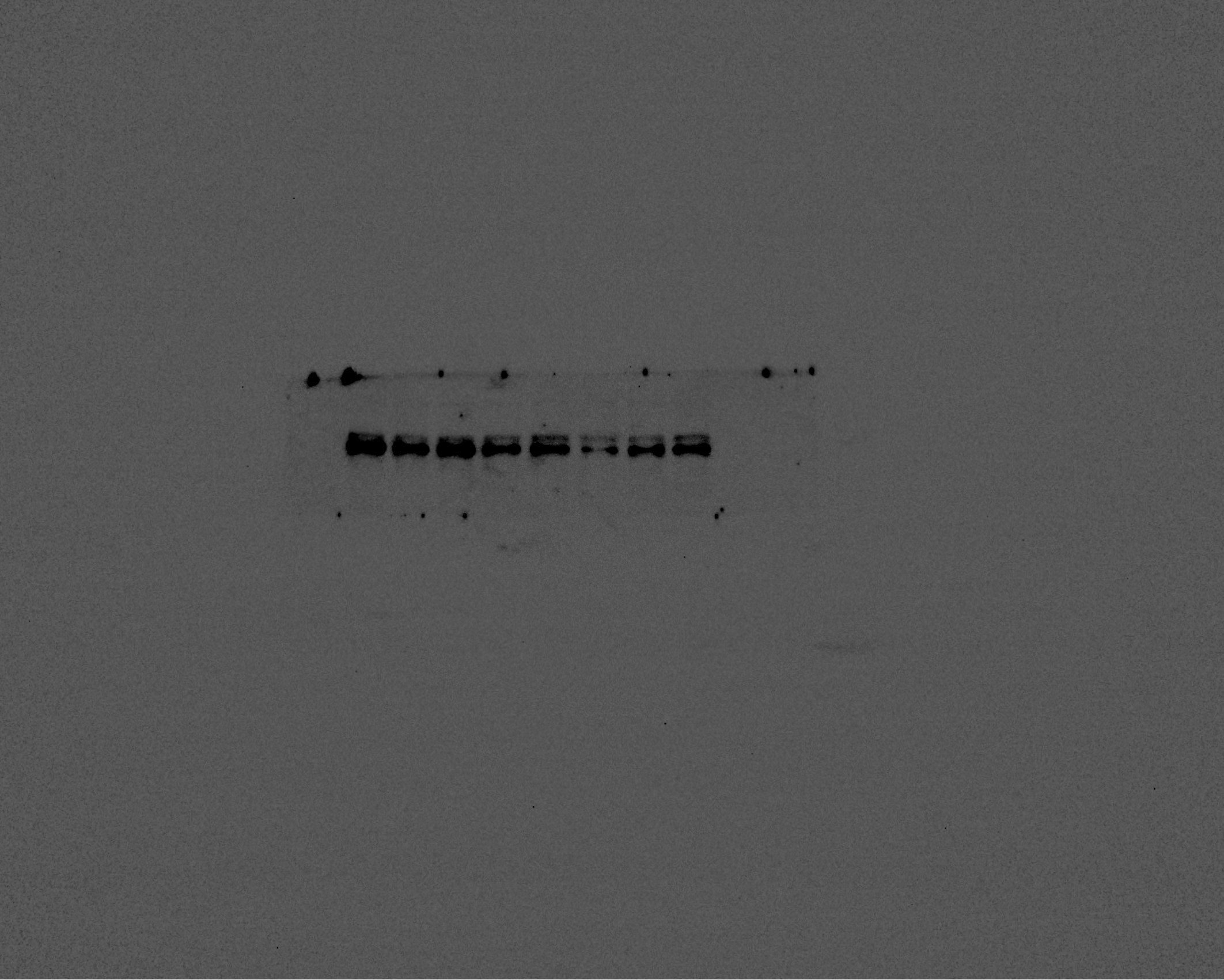

Supplement: Figure 2—source data 3. [file elife-92757-fig2-data3.zip › figure 2 D-E source data 1/8-16 blot 1/tubulin.tif]

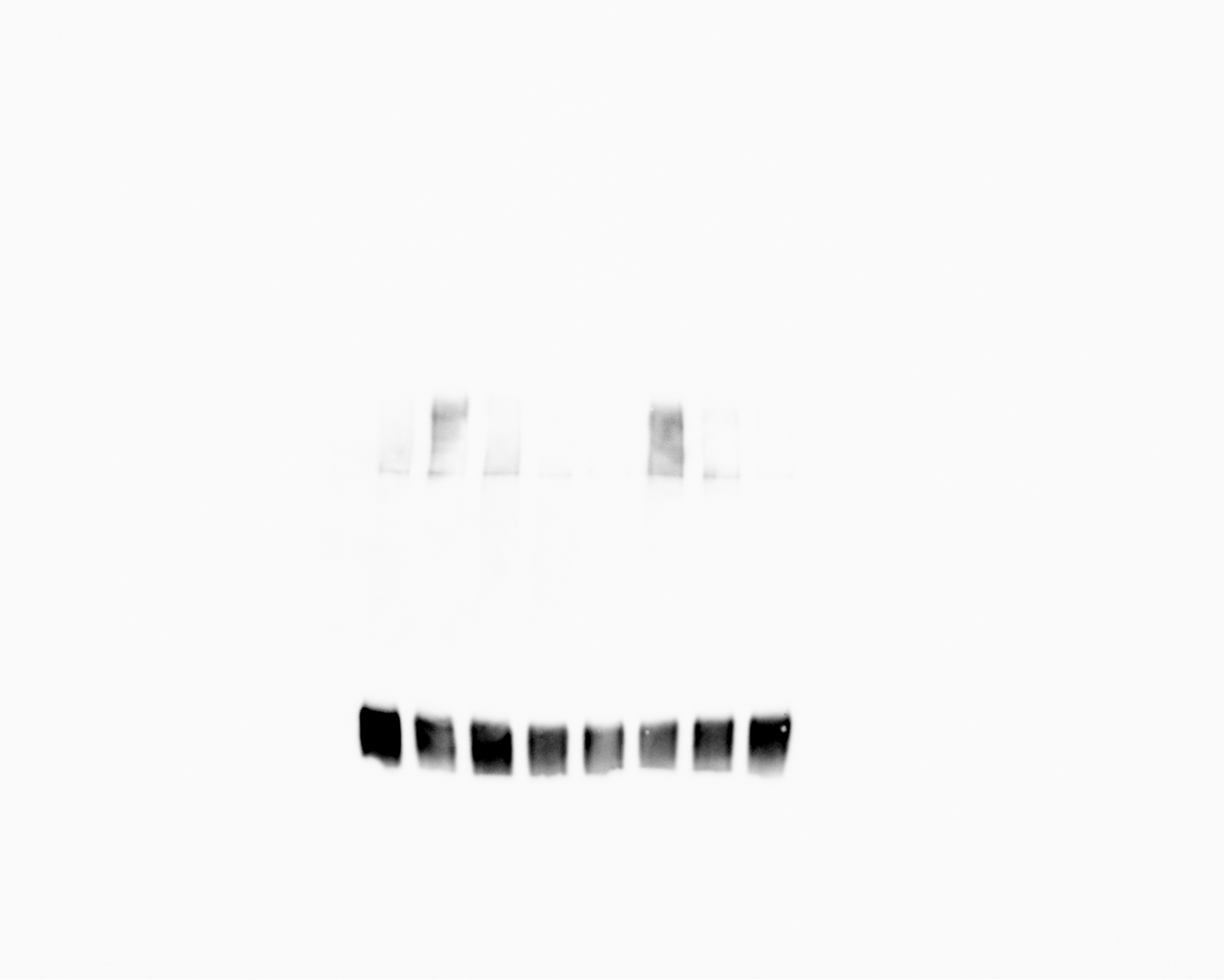

Supplement: Figure 2—source data 3. [file elife-92757-fig2-data3.zip › figure 2 D-E source data 1/8-16 blot 1/igg.tif]

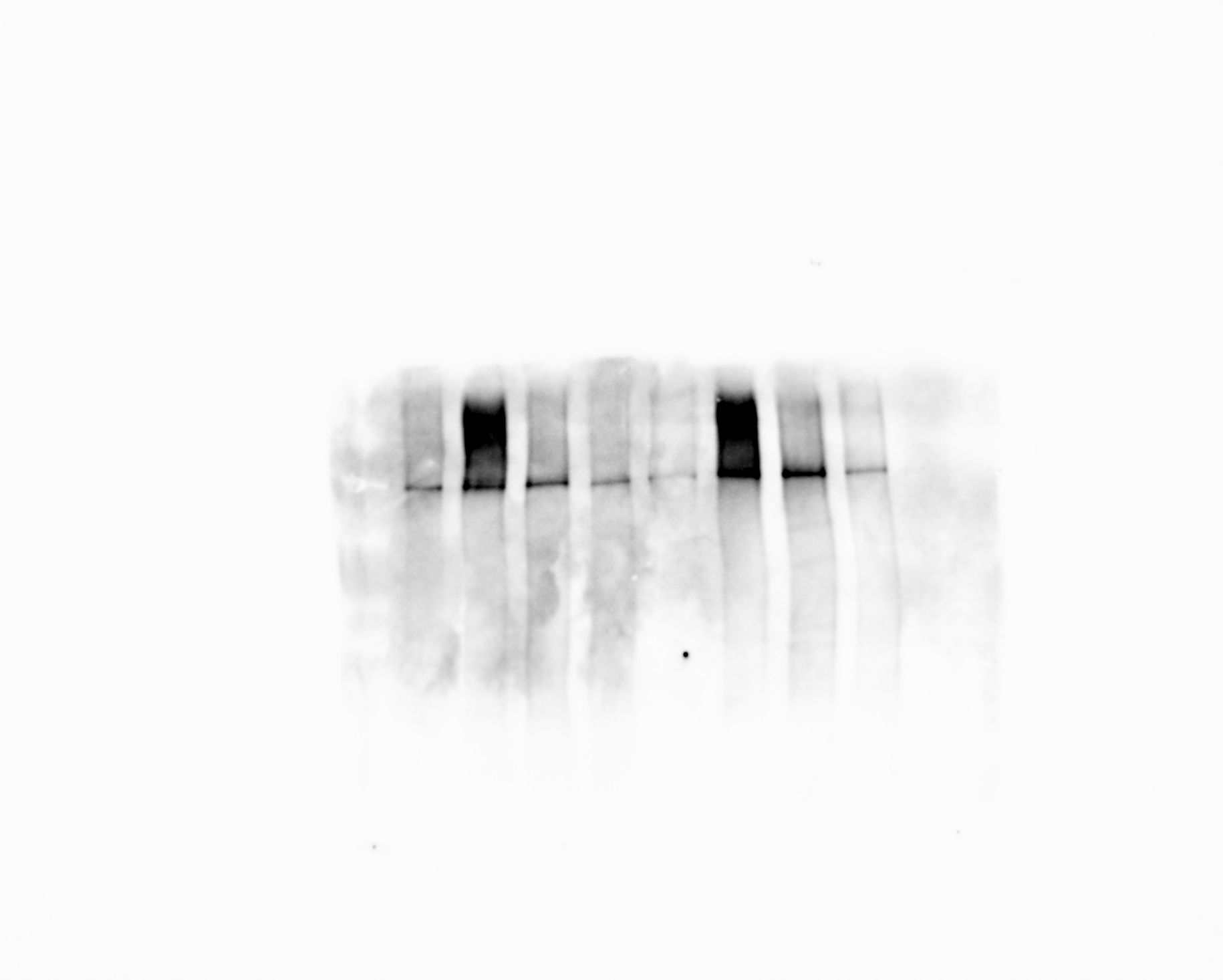

Supplement: Figure 2—source data 3. [file elife-92757-fig2-data3.zip › figure 2 D-E source data 1/8-16 blot 1/ubiquitinated robo .tif]

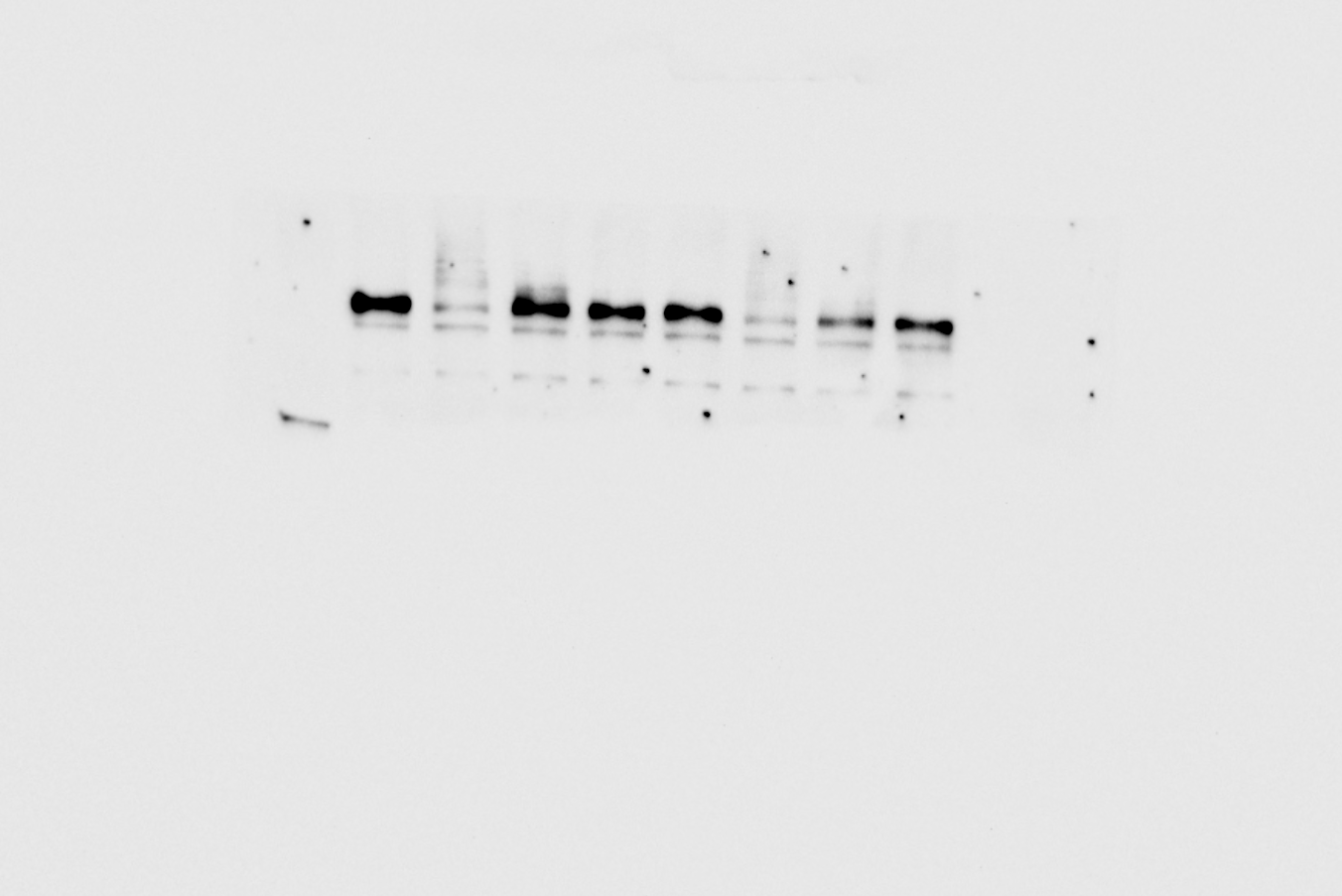

Supplement: Figure 2—source data 3. [file elife-92757-fig2-data3.zip › figure 2 D-E source data 1/8-16 blot 1/robo lysate.tif]

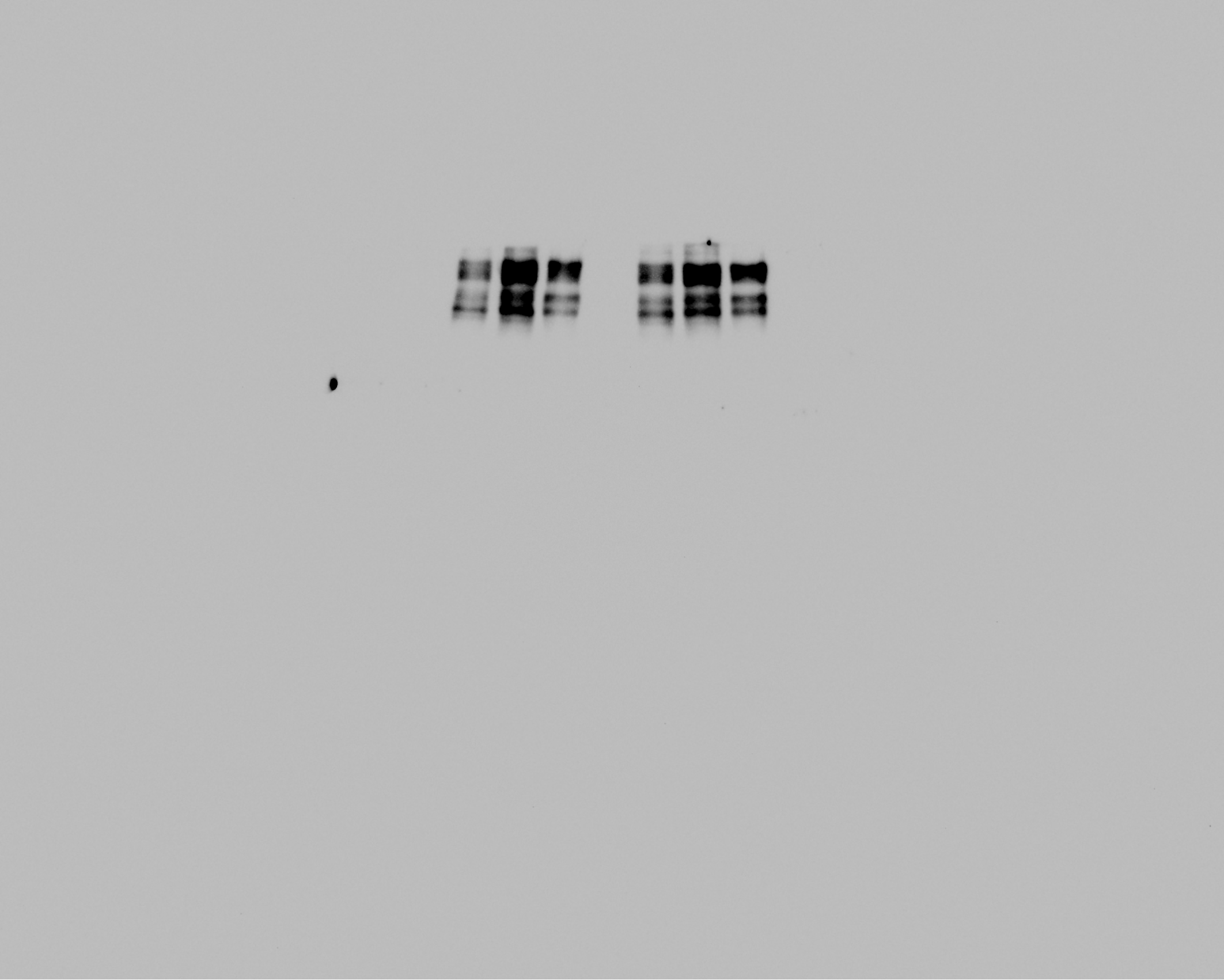

Supplement: Figure 2—source data 3. [file elife-92757-fig2-data3.zip › figure 2 D-E source data 1/8-16 blot 1/comm lysate.tif]

8-16 IP 1

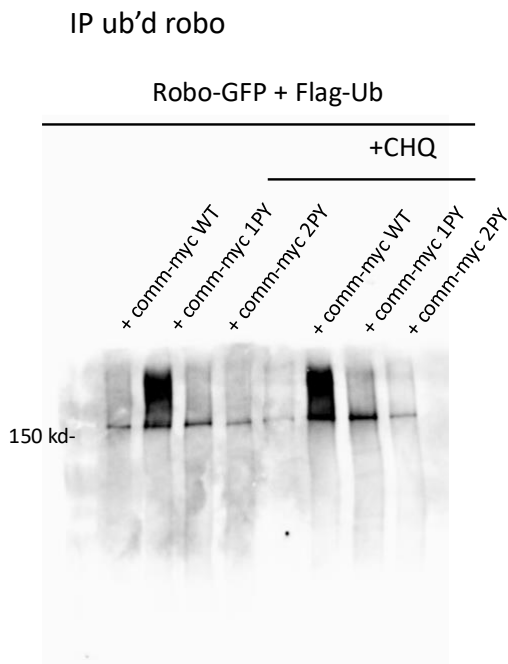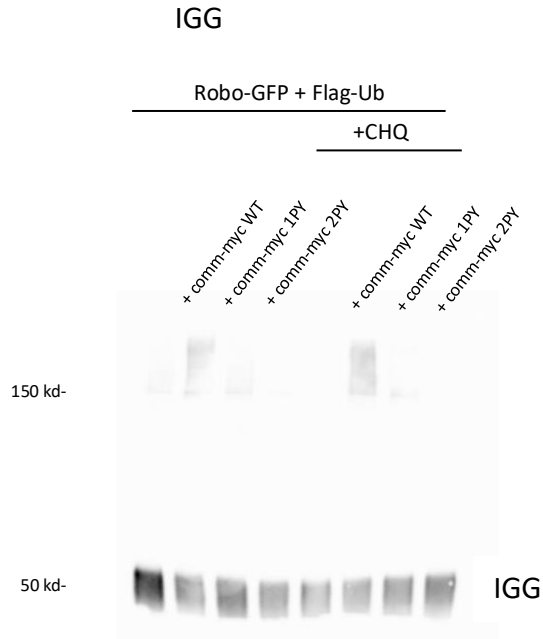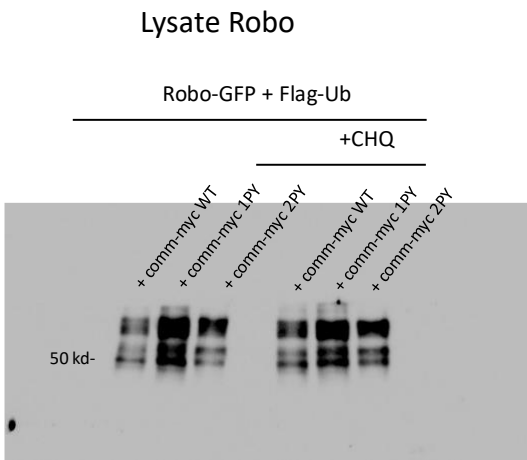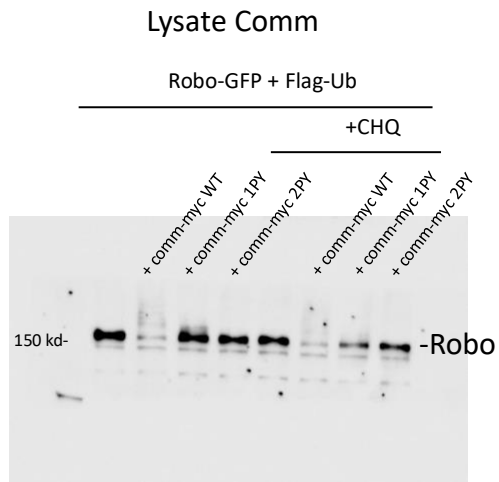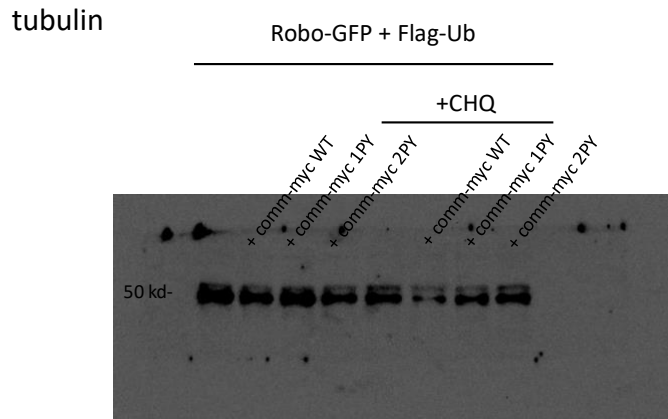

8-25 IP 2

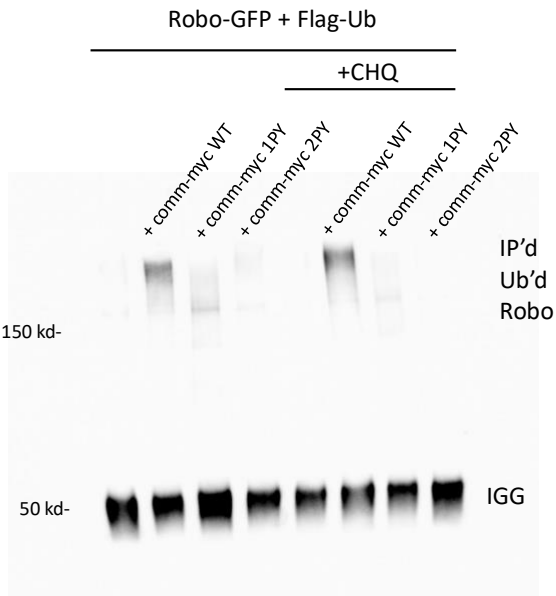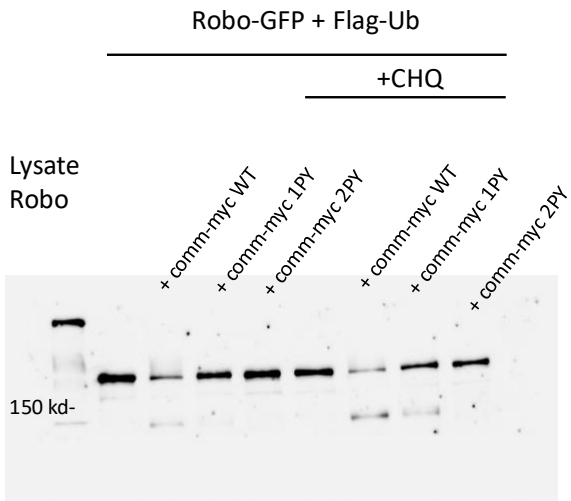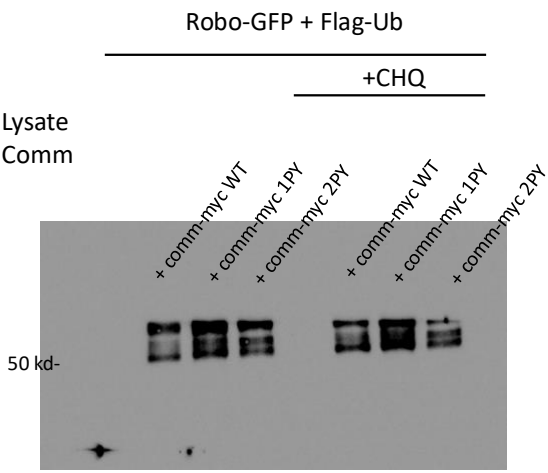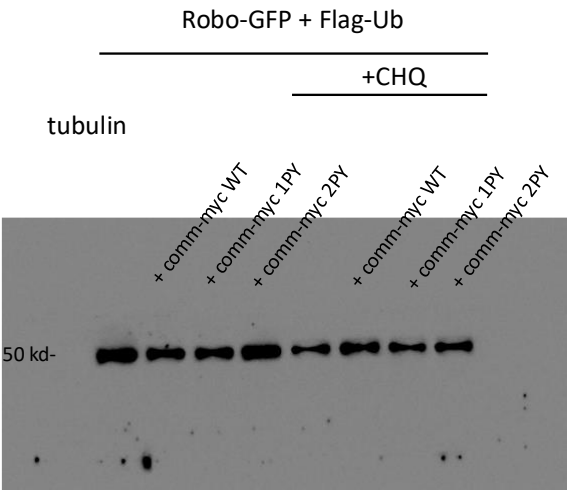

10-3 IP 3

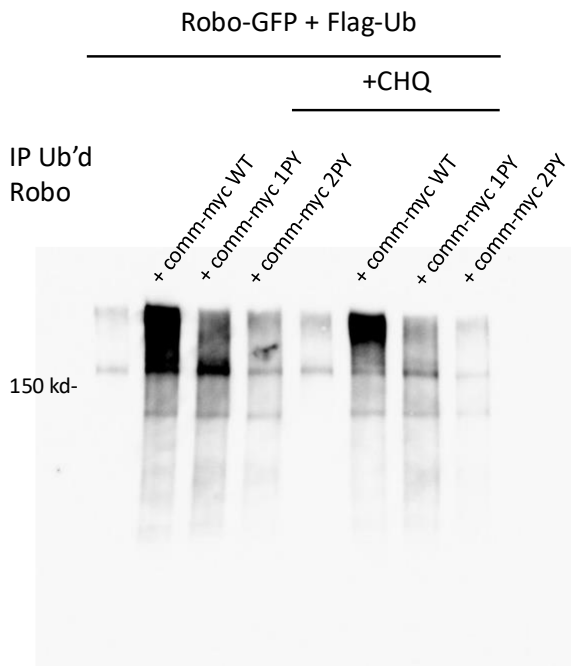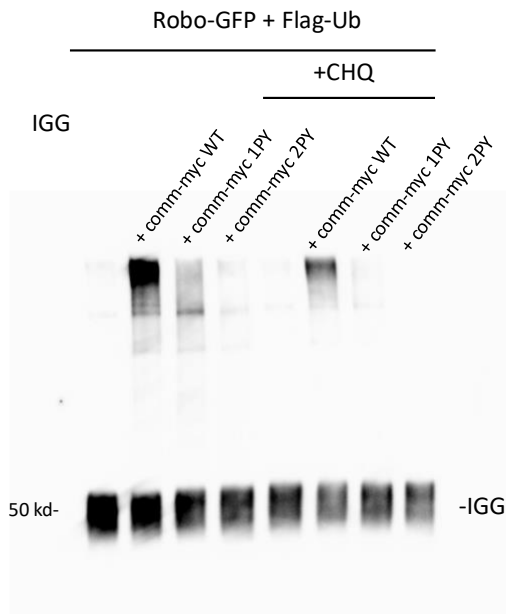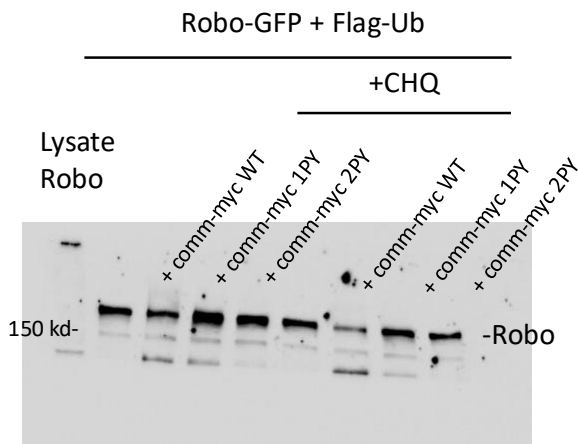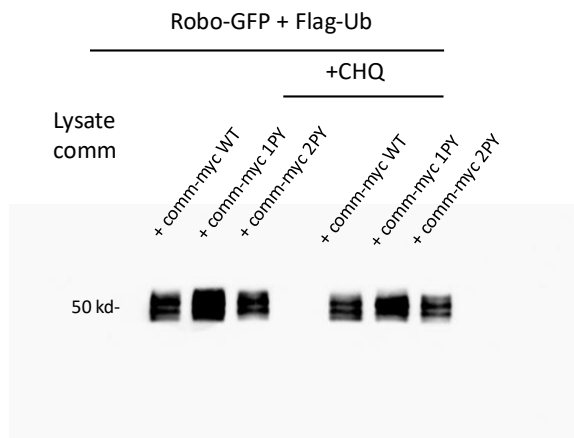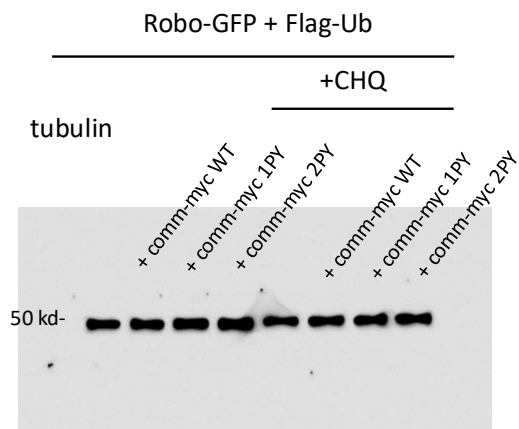

10-12 IP 4

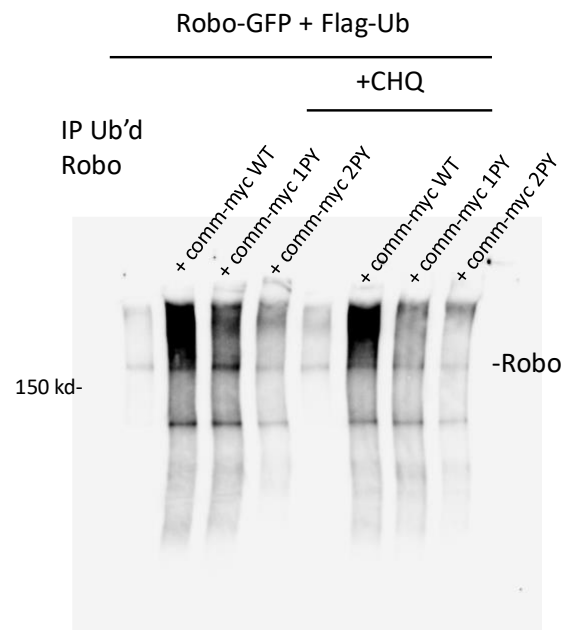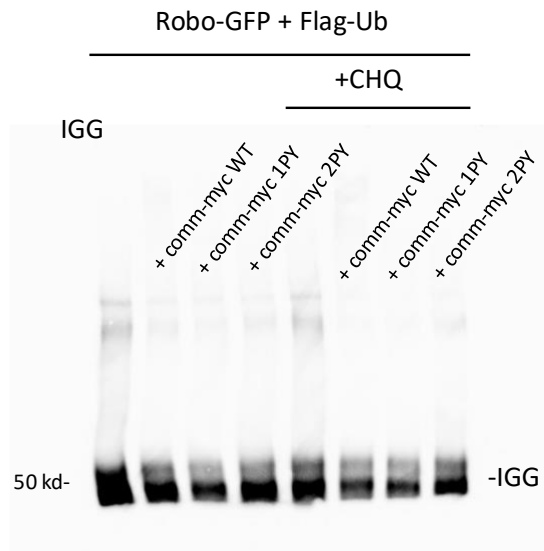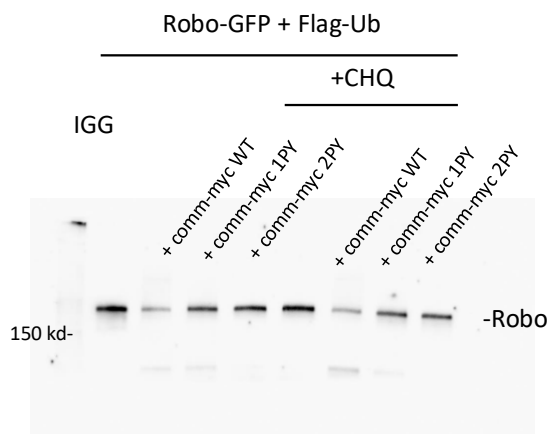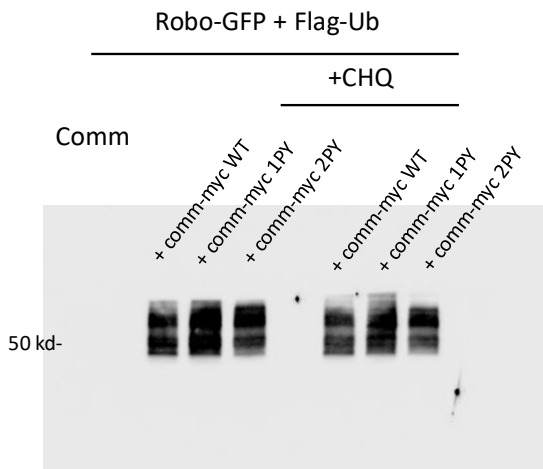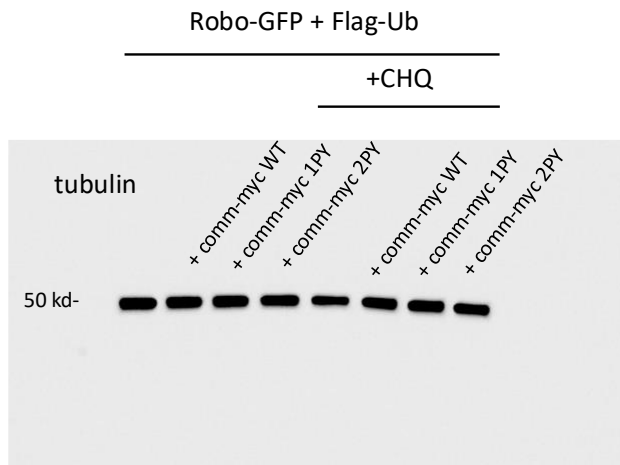

Supplement: Figure 2—source data 4. [file elife-92757-fig2-data4.zip › figure 2 D-E source data 2/IP blots.pdf]

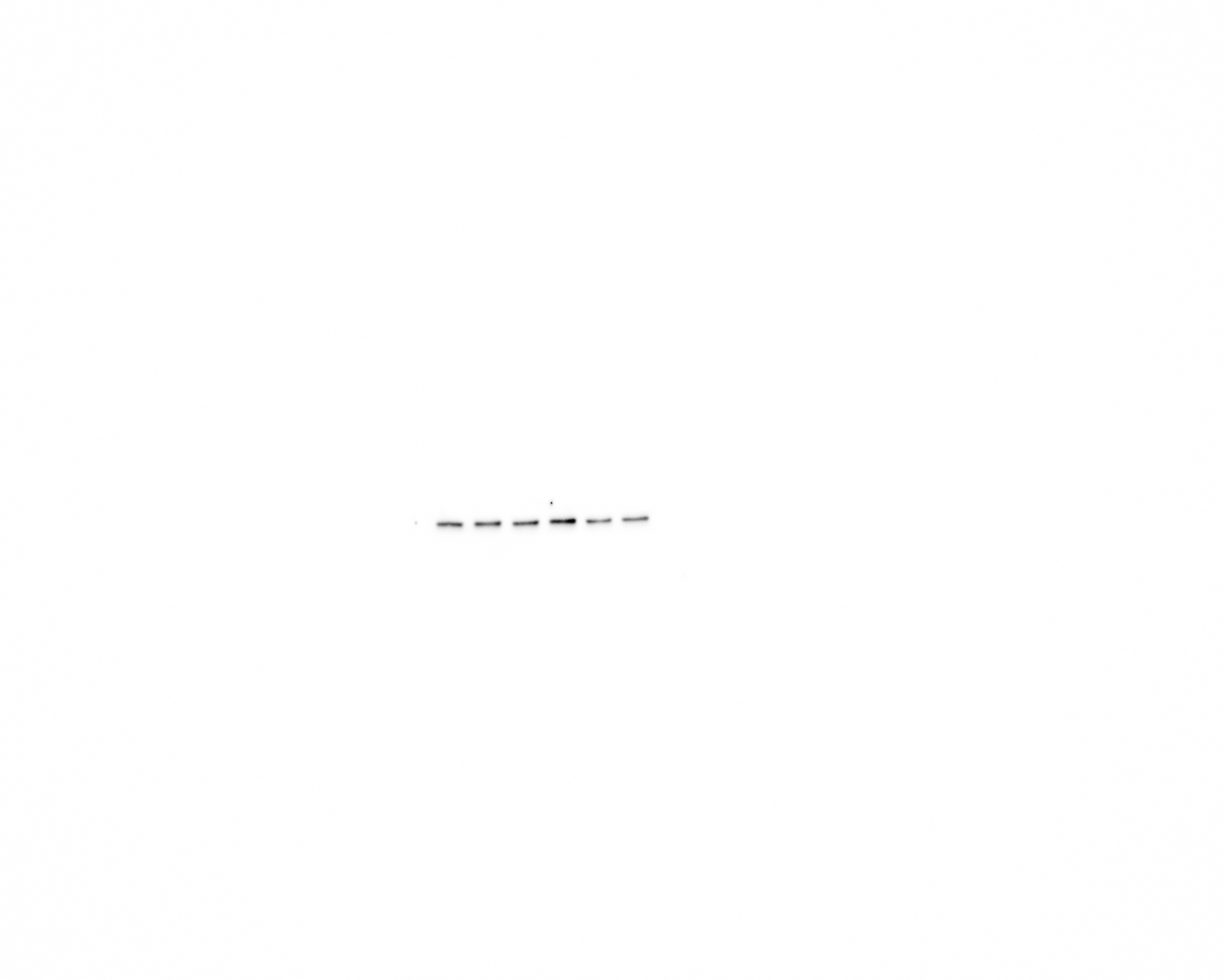

Supplement: Figure 7—source data 1. [file elife-92757-fig7-data1.zip › Fig 7 A-C source data 1/3-9 IP 2/tubulin.tif]

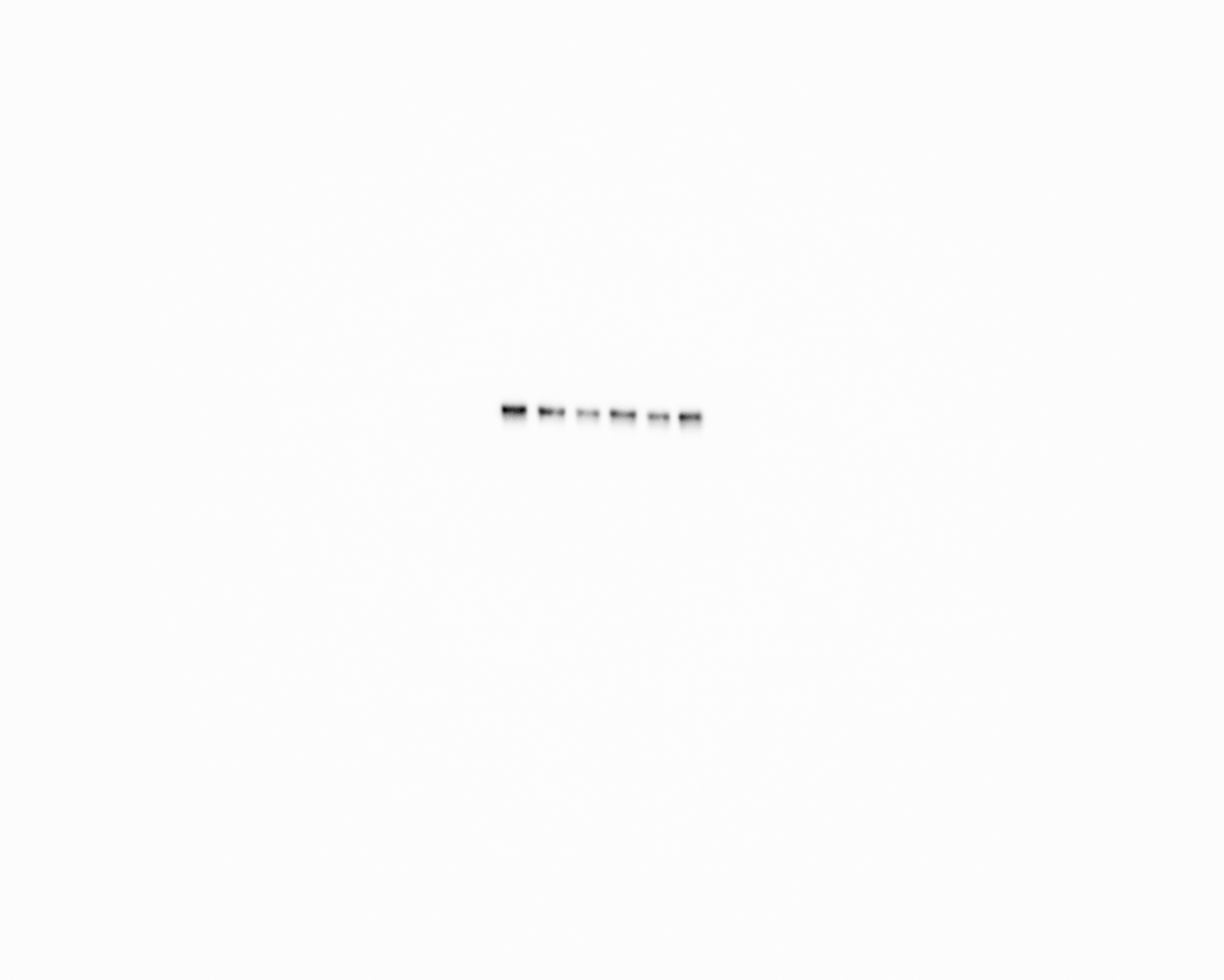

Supplement: Figure 7—source data 1. [file elife-92757-fig7-data1.zip › Fig 7 A-C source data 1/3-9 IP 2/IGG.tif]

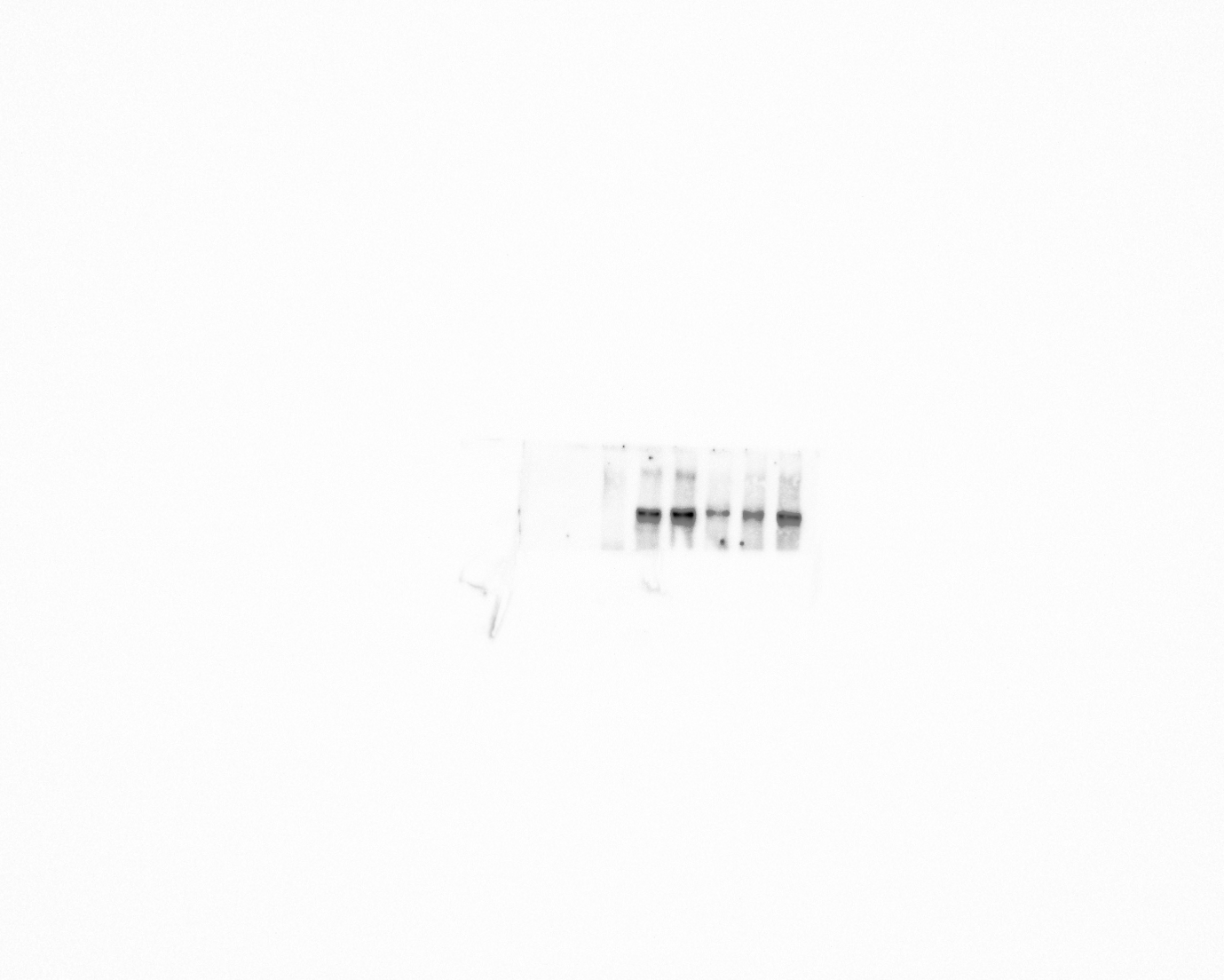

Supplement: Figure 7—source data 1. [file elife-92757-fig7-data1.zip › Fig 7 A-C source data 1/3-9 IP 2/ip robo.tif]

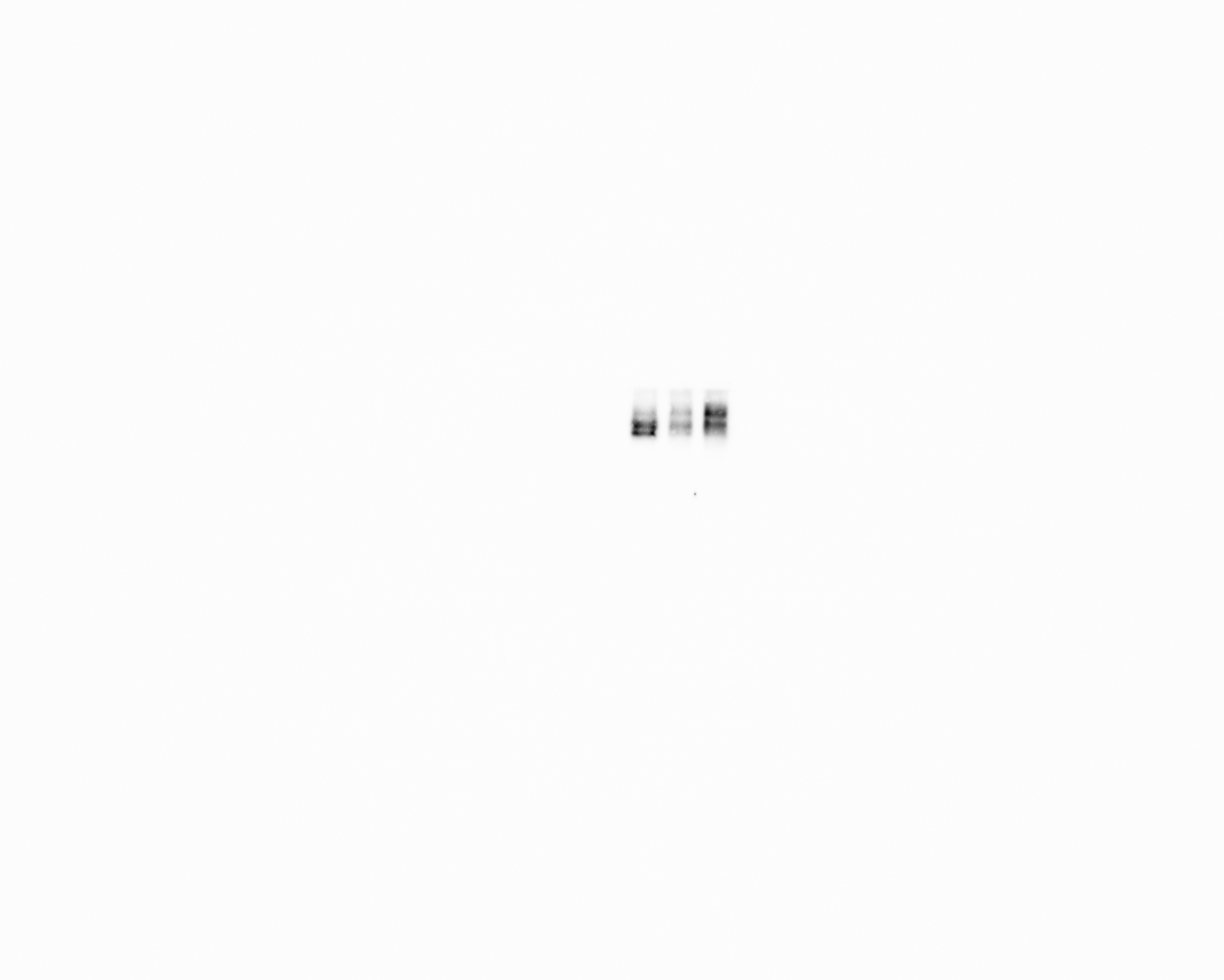

Supplement: Figure 7—source data 1. [file elife-92757-fig7-data1.zip › Fig 7 A-C source data 1/3-9 IP 2/ip comm.tif]

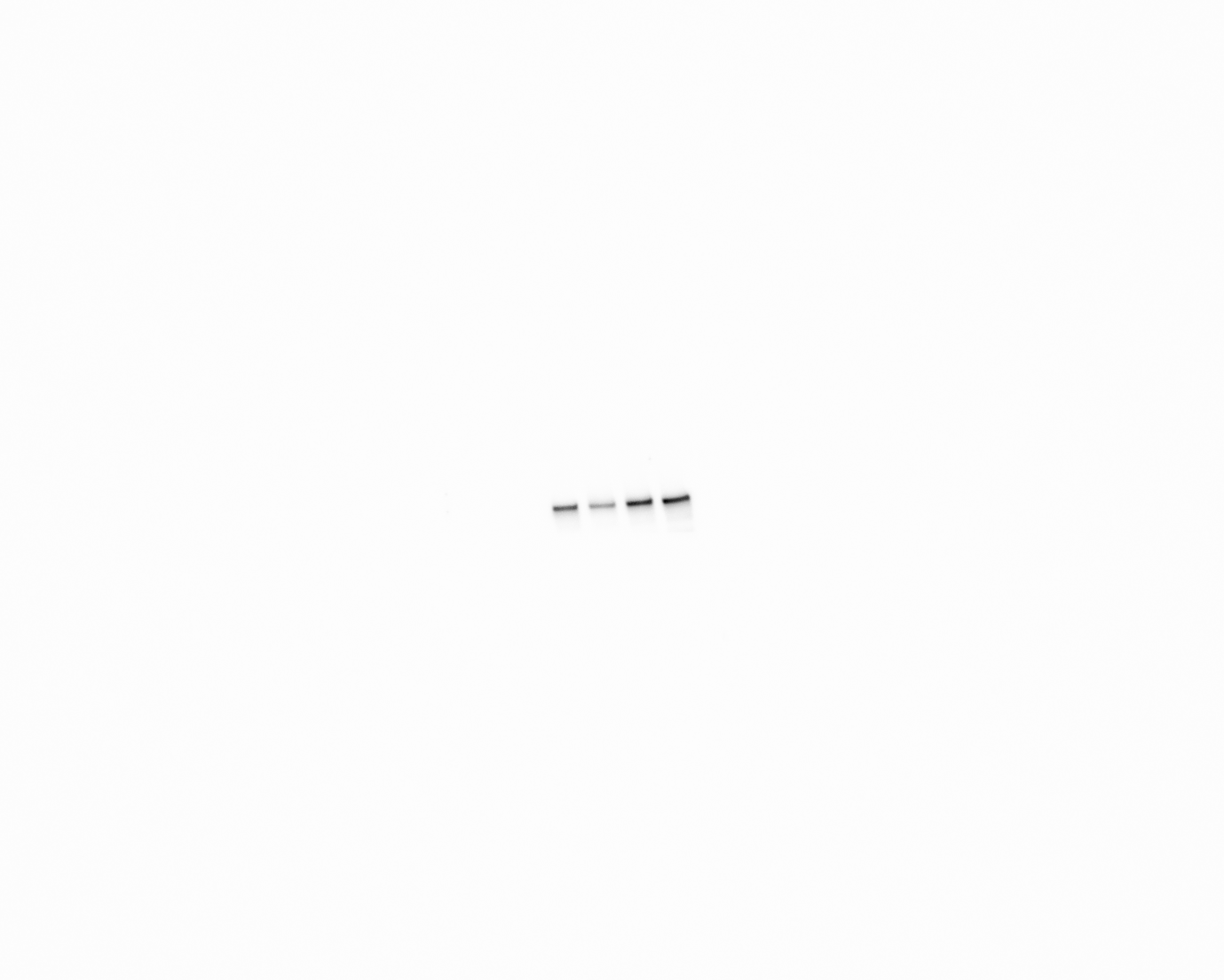

Supplement: Figure 7—source data 1. [file elife-92757-fig7-data1.zip › Fig 7 A-C source data 1/3-9 IP 2/lysate nedd4.tif]

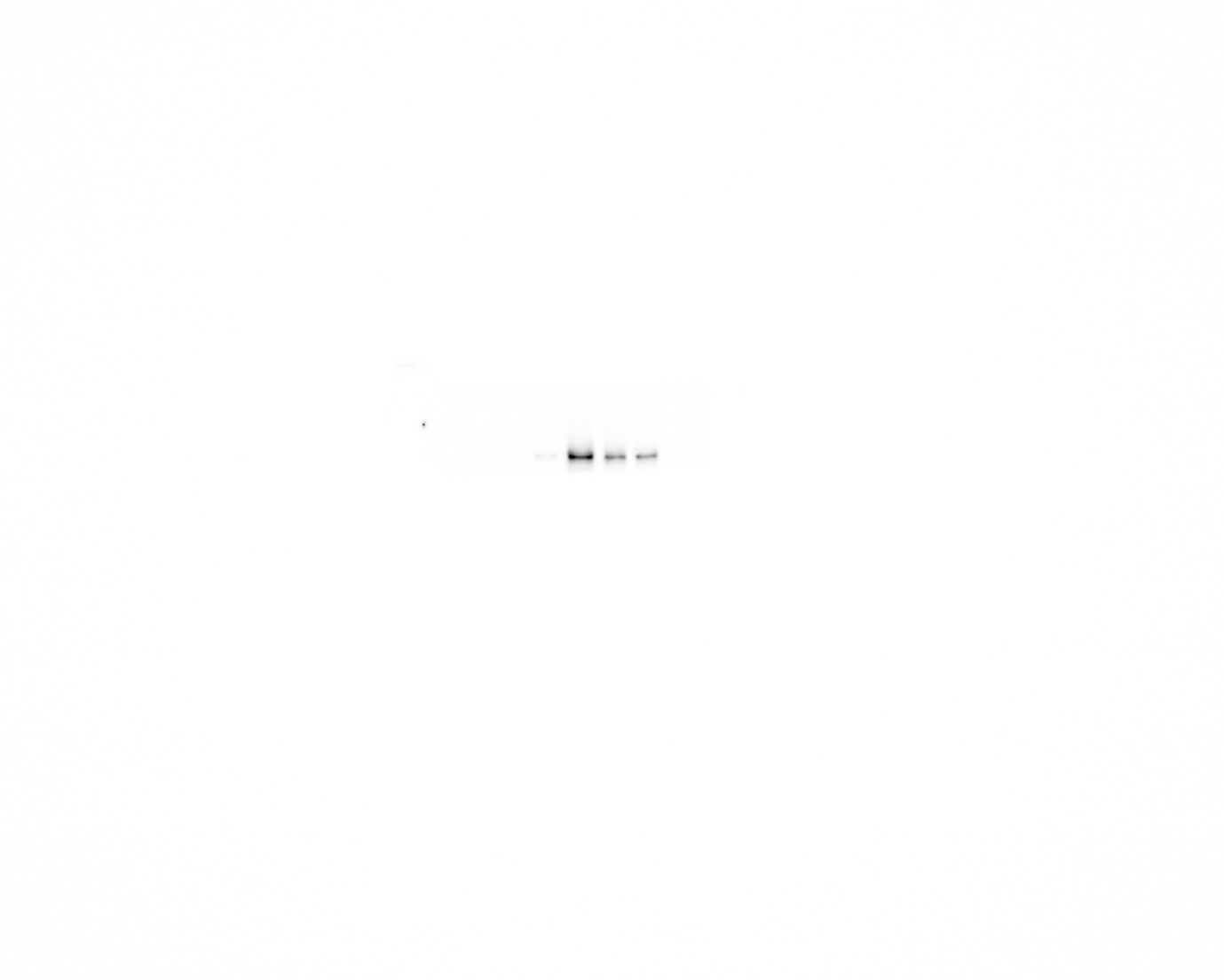

Supplement: Figure 7—source data 1. [file elife-92757-fig7-data1.zip › Fig 7 A-C source data 1/3-9 IP 2/ip nedd4.tif]

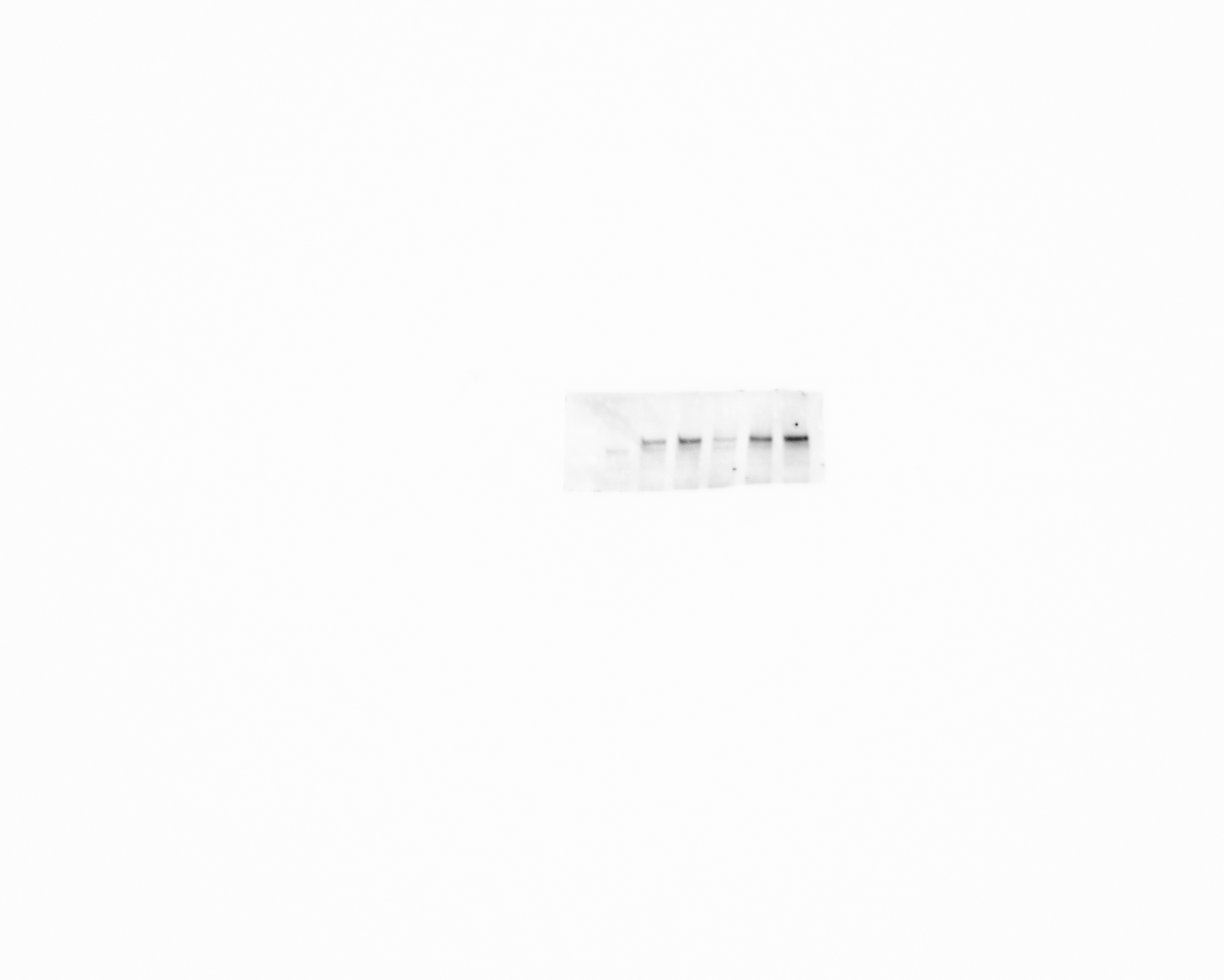

Supplement: Figure 7—source data 1. [file elife-92757-fig7-data1.zip › Fig 7 A-C source data 1/3-9 IP 2/robo lysate.tif]

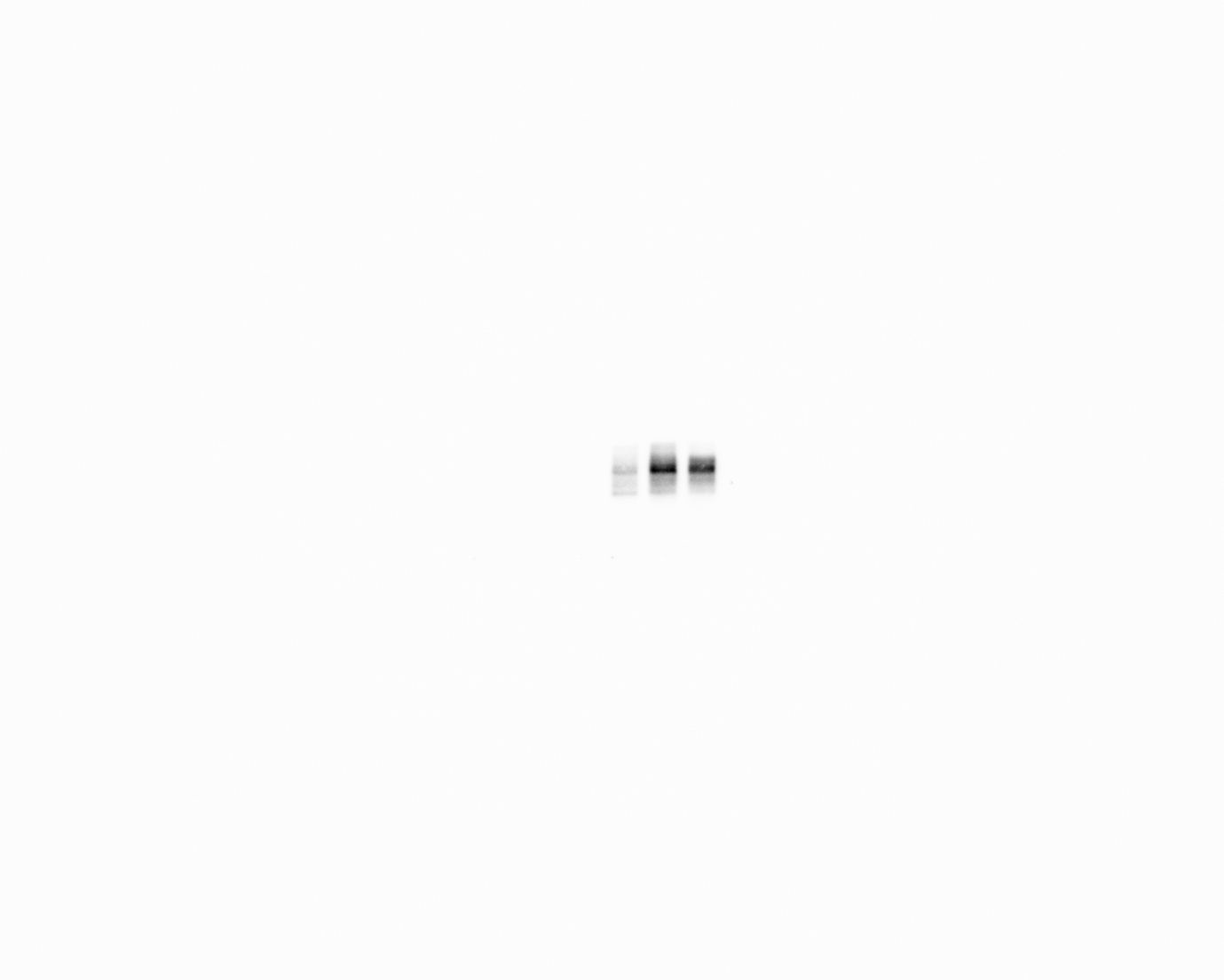

Supplement: Figure 7—source data 1. [file elife-92757-fig7-data1.zip › Fig 7 A-C source data 1/3-9 IP 2/lysate comm.tif]

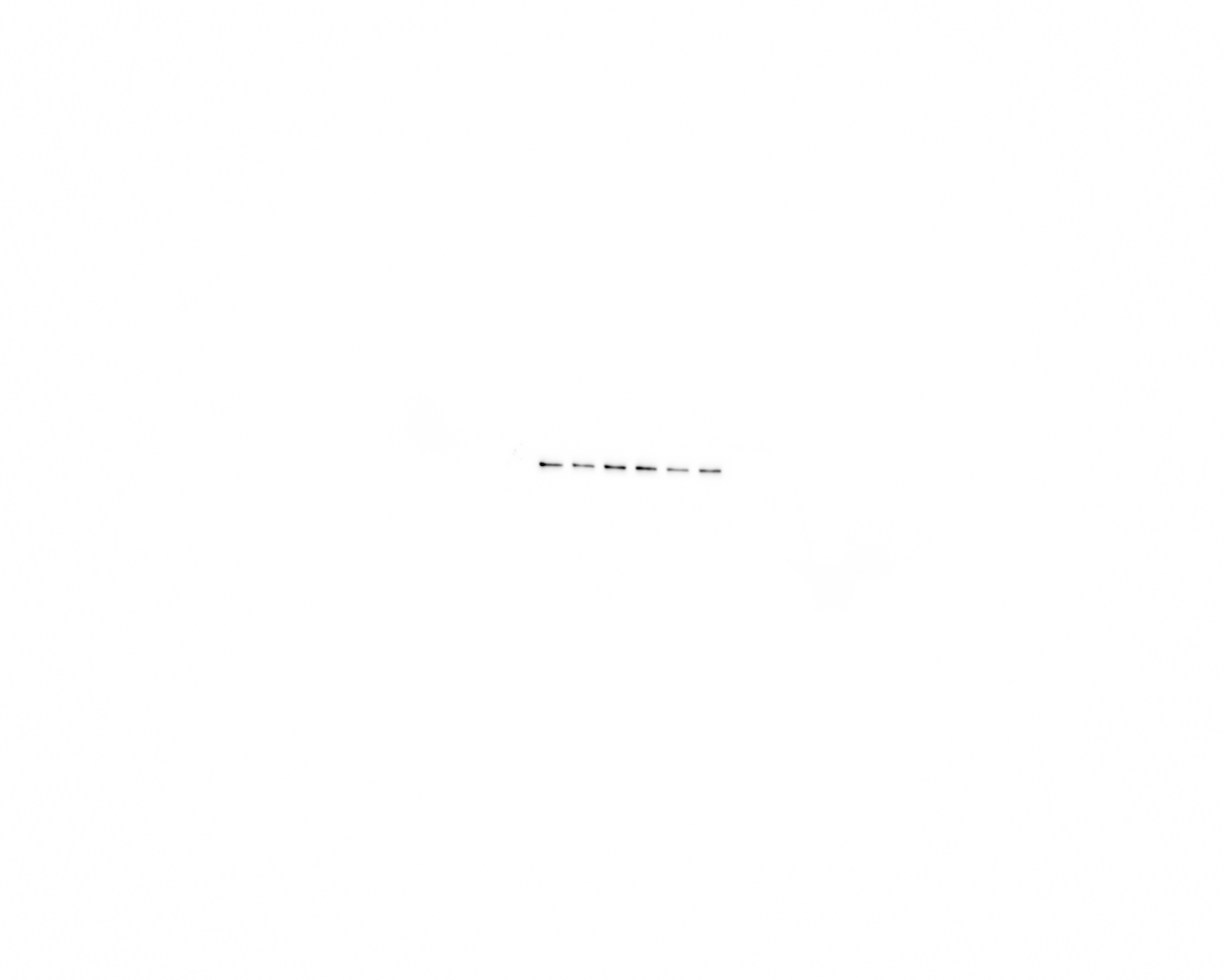

Supplement: Figure 7—source data 1. [file elife-92757-fig7-data1.zip › Fig 7 A-C source data 1/6-8 IP 3/tubulin.tif]

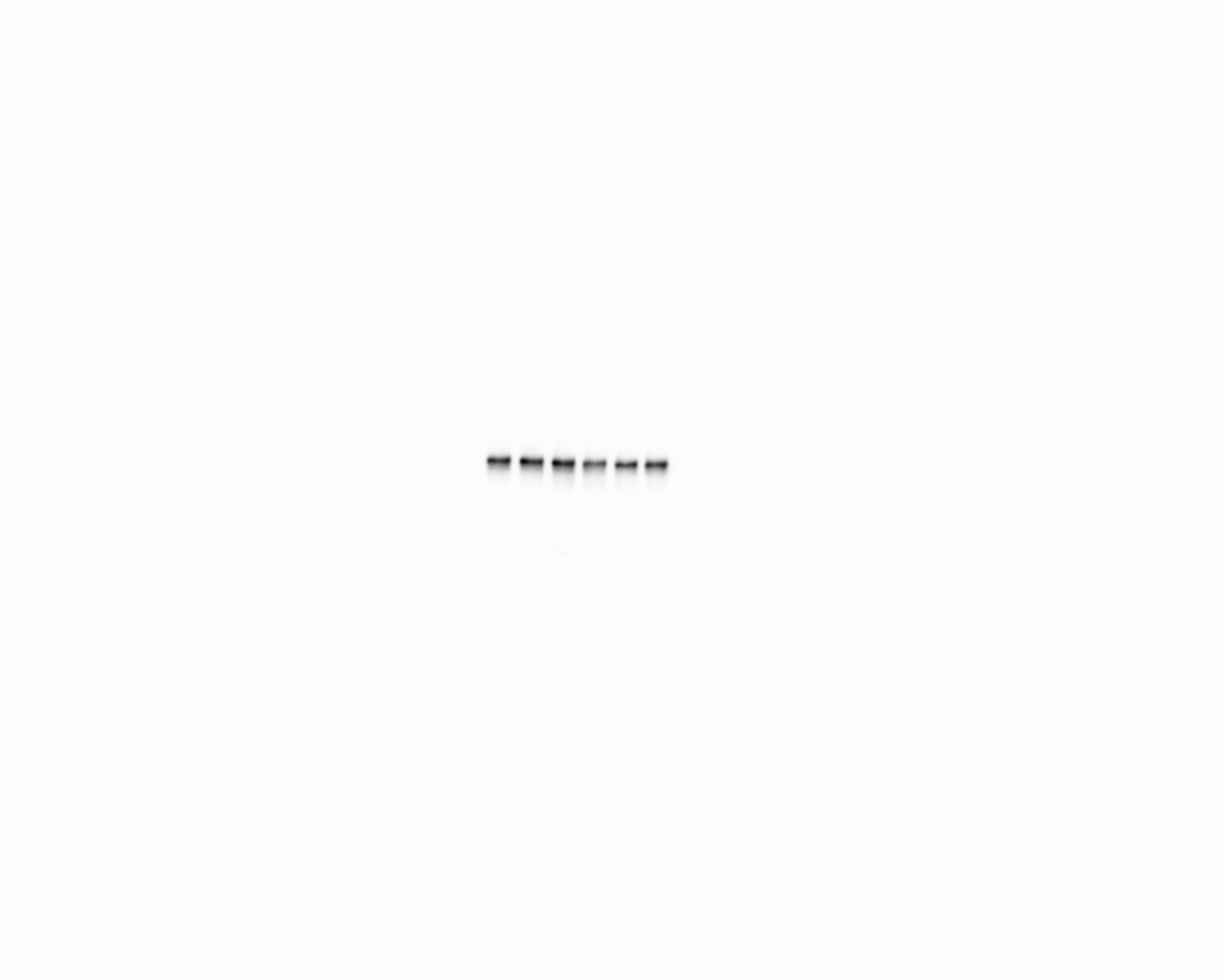

Supplement: Figure 7—source data 1. [file elife-92757-fig7-data1.zip › Fig 7 A-C source data 1/6-8 IP 3/igg.tif]

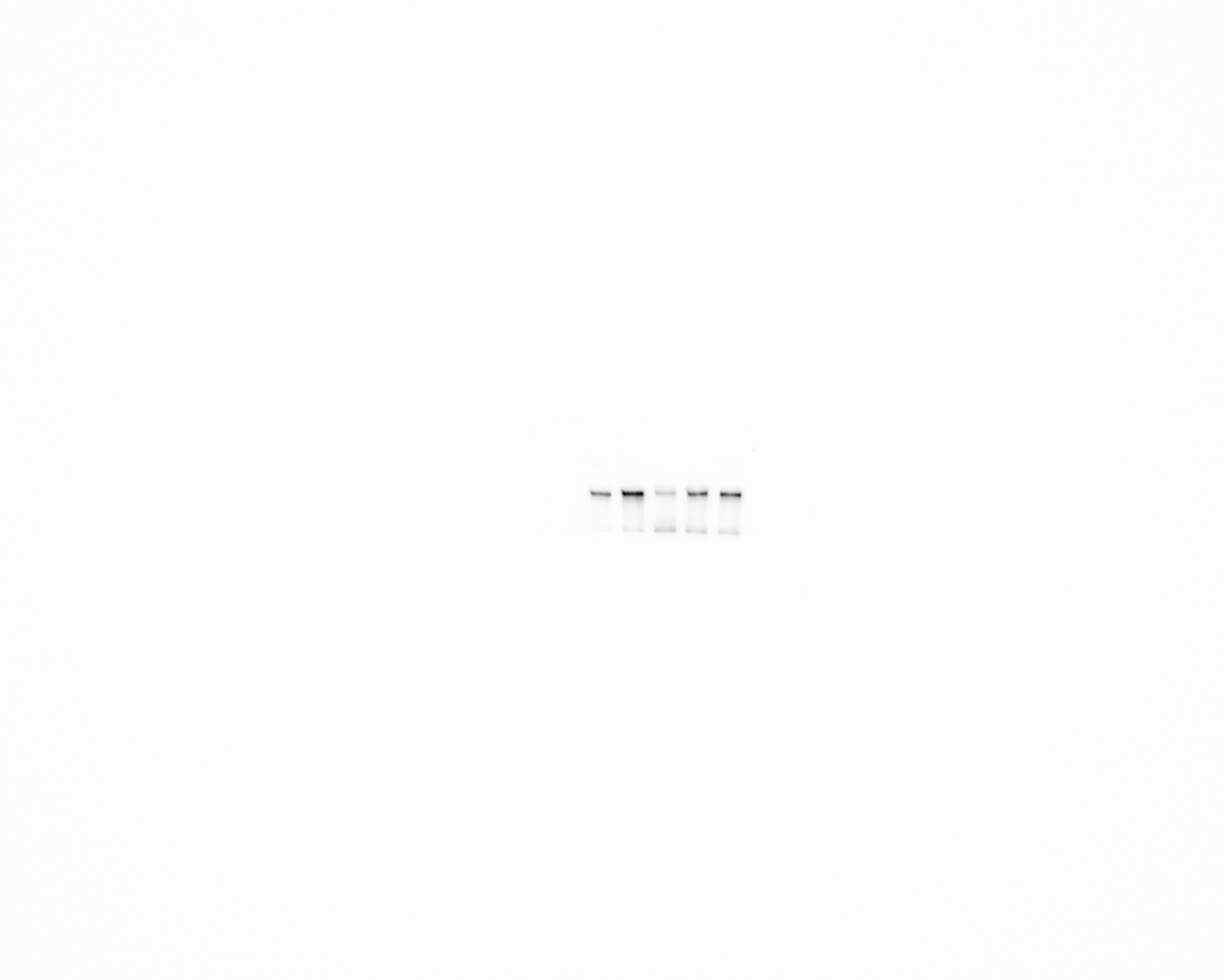

Supplement: Figure 7—source data 1. [file elife-92757-fig7-data1.zip › Fig 7 A-C source data 1/6-8 IP 3/IP robo.tif]

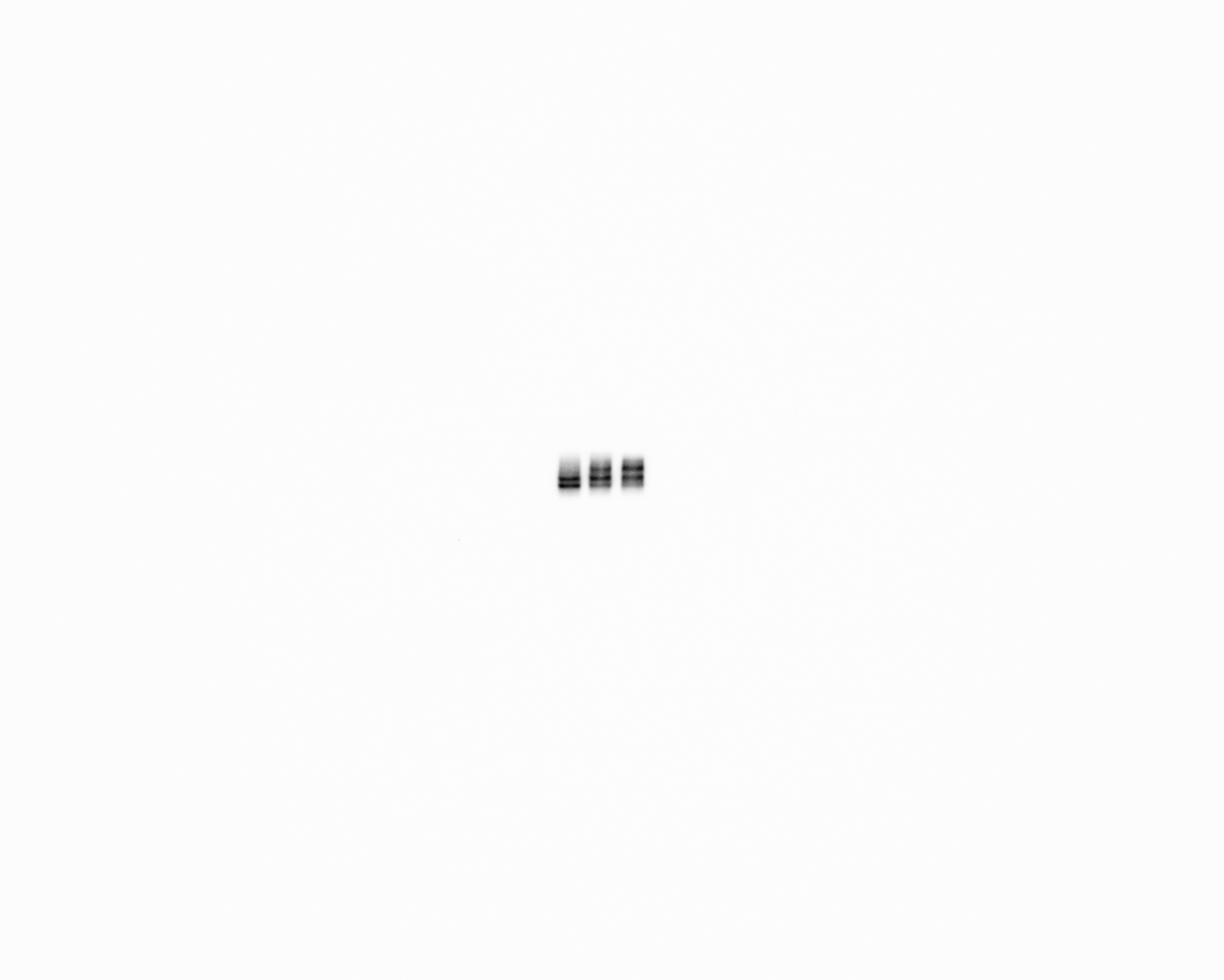

Supplement: Figure 7—source data 1. [file elife-92757-fig7-data1.zip › Fig 7 A-C source data 1/6-8 IP 3/IP comm.tif]

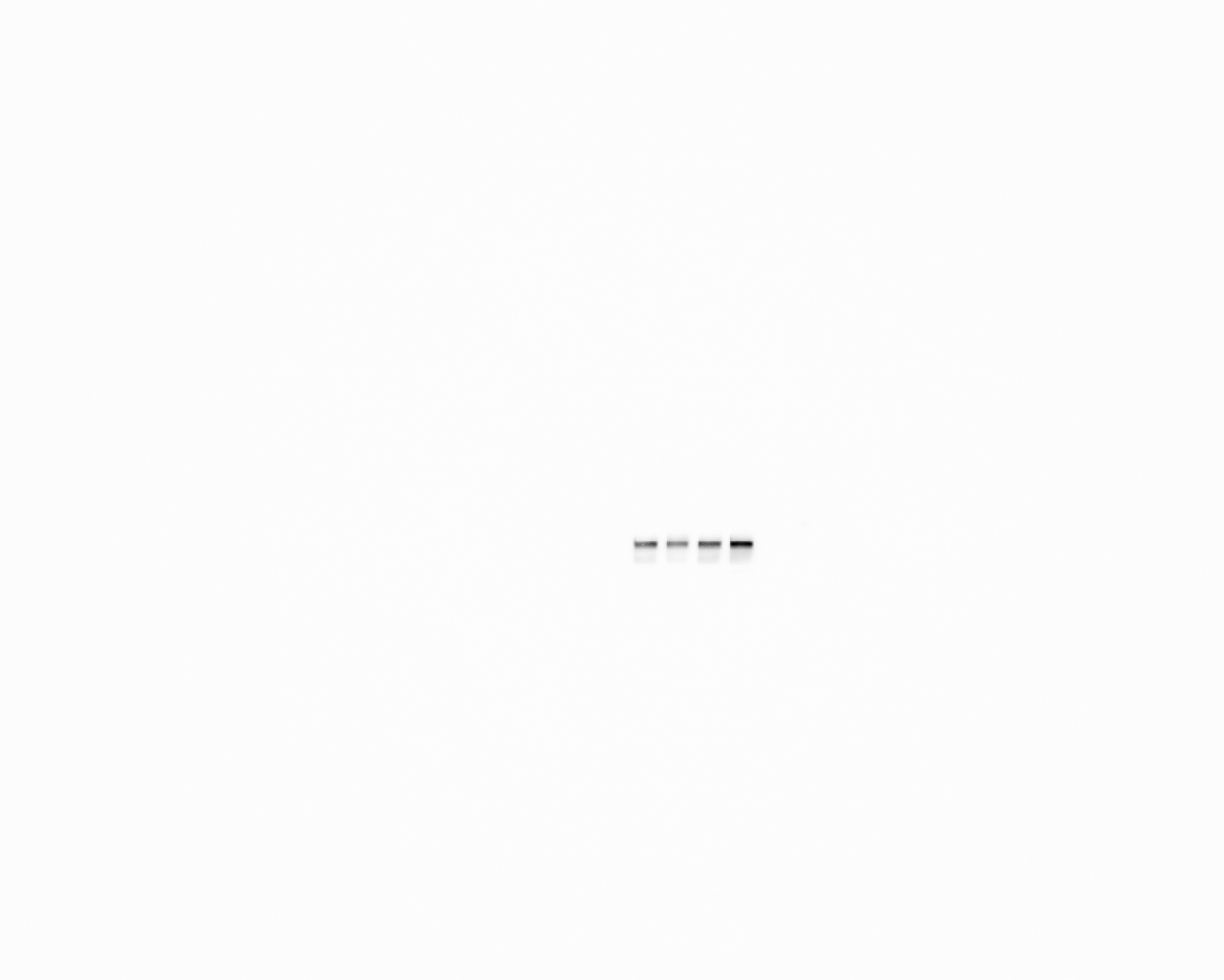

Supplement: Figure 7—source data 1. [file elife-92757-fig7-data1.zip › Fig 7 A-C source data 1/6-8 IP 3/lysate nedd4.tif]

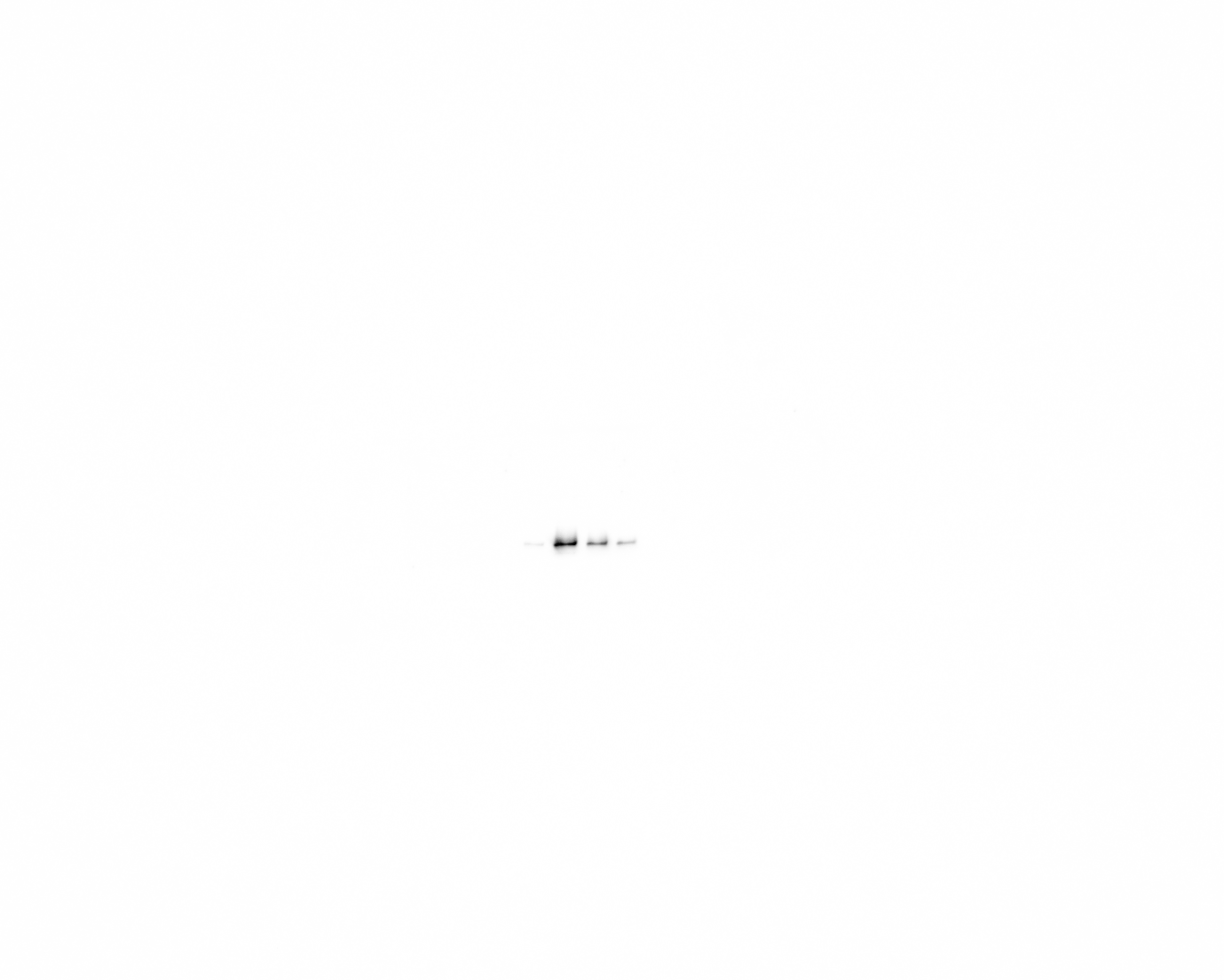

Supplement: Figure 7—source data 1. [file elife-92757-fig7-data1.zip › Fig 7 A-C source data 1/6-8 IP 3/ip nedd4.tif]

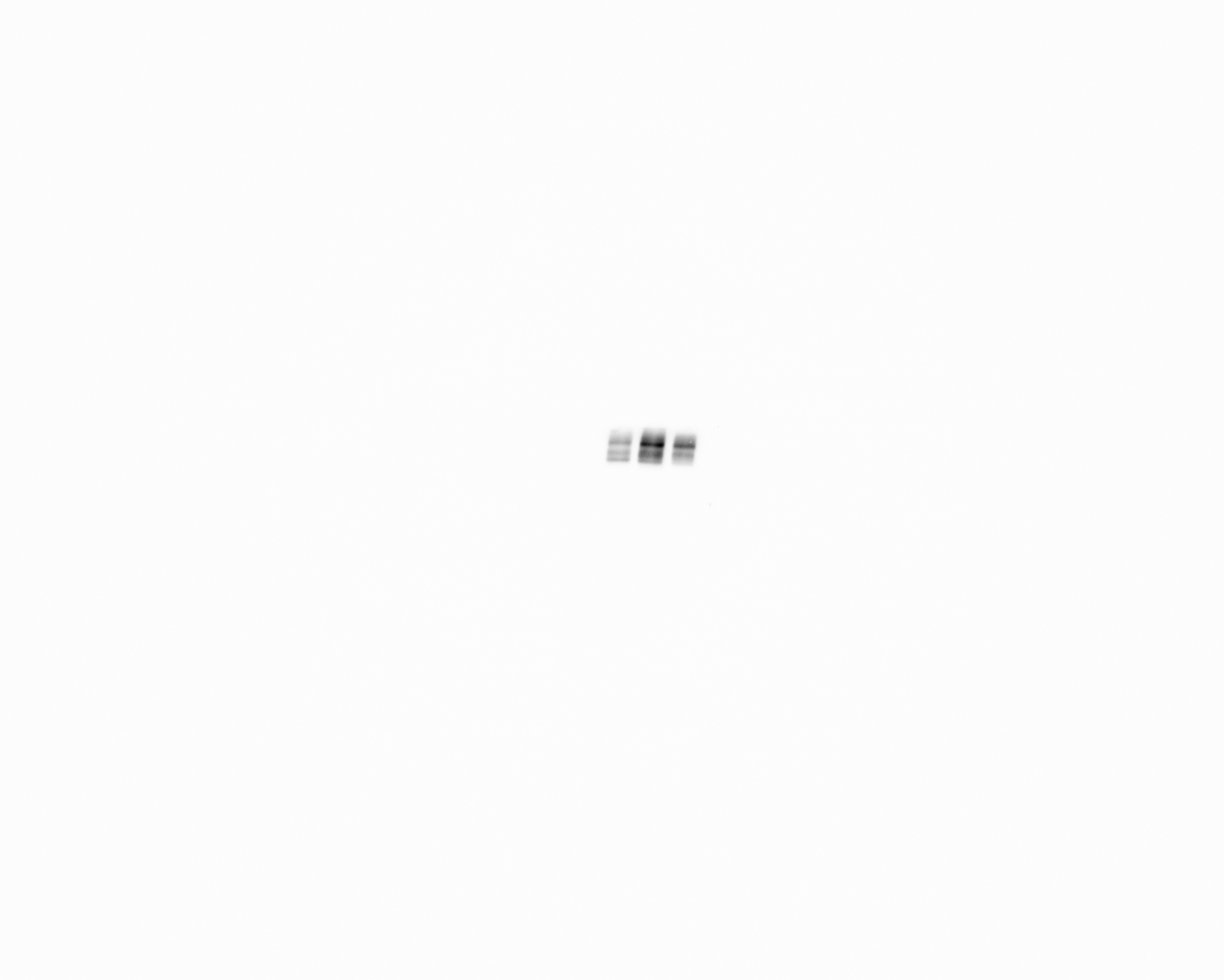

Supplement: Figure 7—source data 1. [file elife-92757-fig7-data1.zip › Fig 7 A-C source data 1/6-8 IP 3/lysate comm.tif]

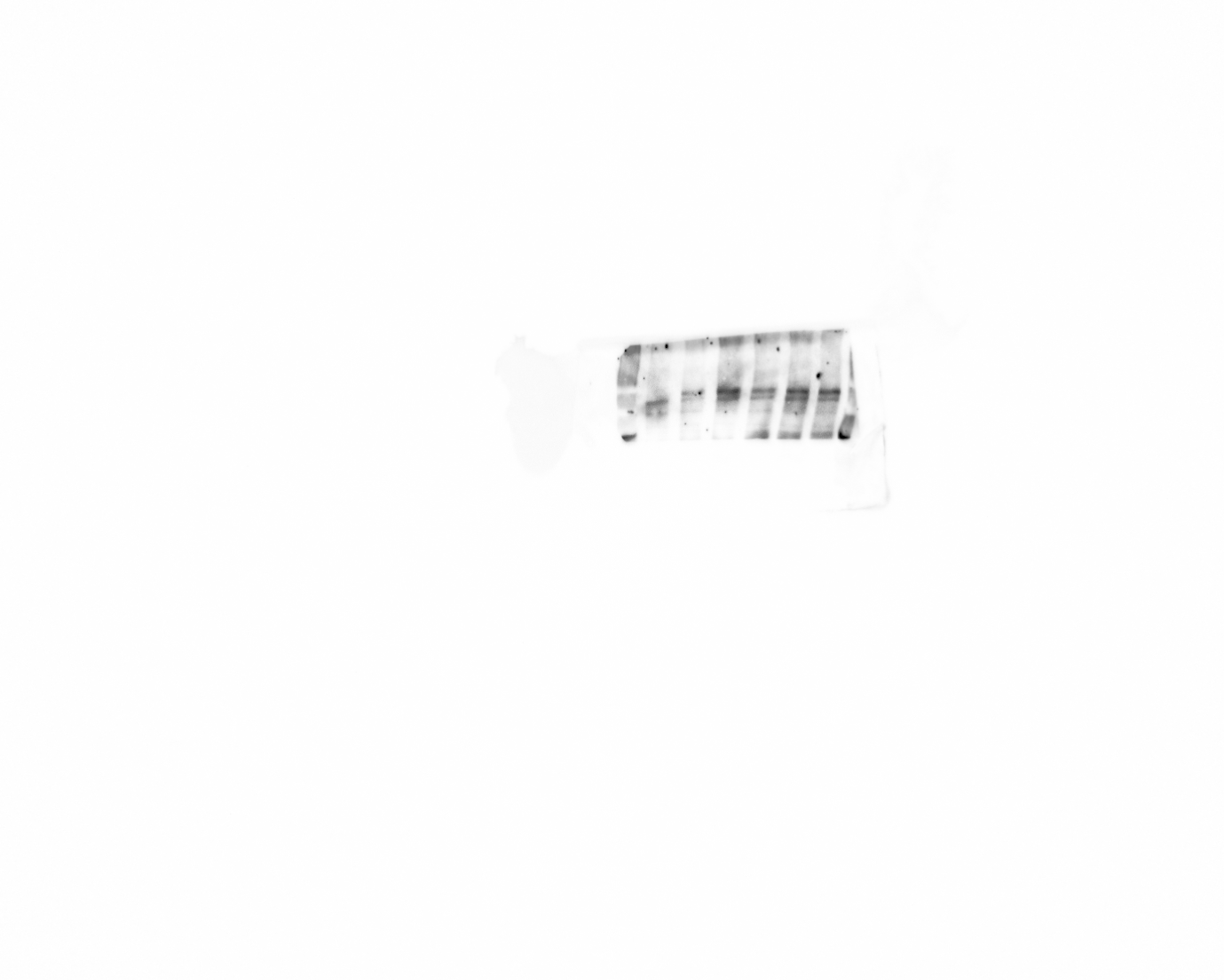

Supplement: Figure 7—source data 1. [file elife-92757-fig7-data1.zip › Fig 7 A-C source data 1/6-8 IP 3/lysate robo.tif]

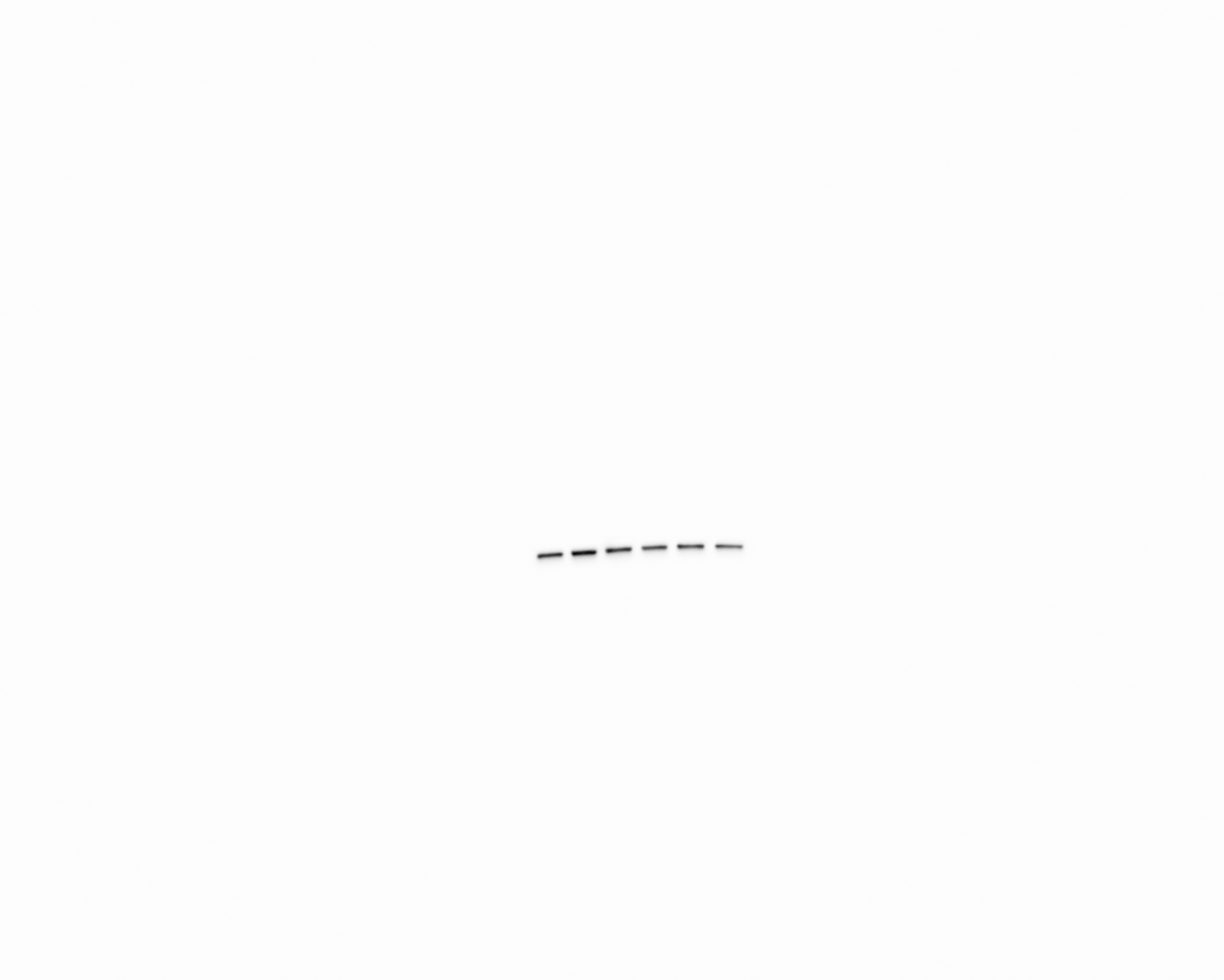

Supplement: Figure 7—source data 1. [file elife-92757-fig7-data1.zip › Fig 7 A-C source data 1/10-20 IP 1/tubulin.tif]

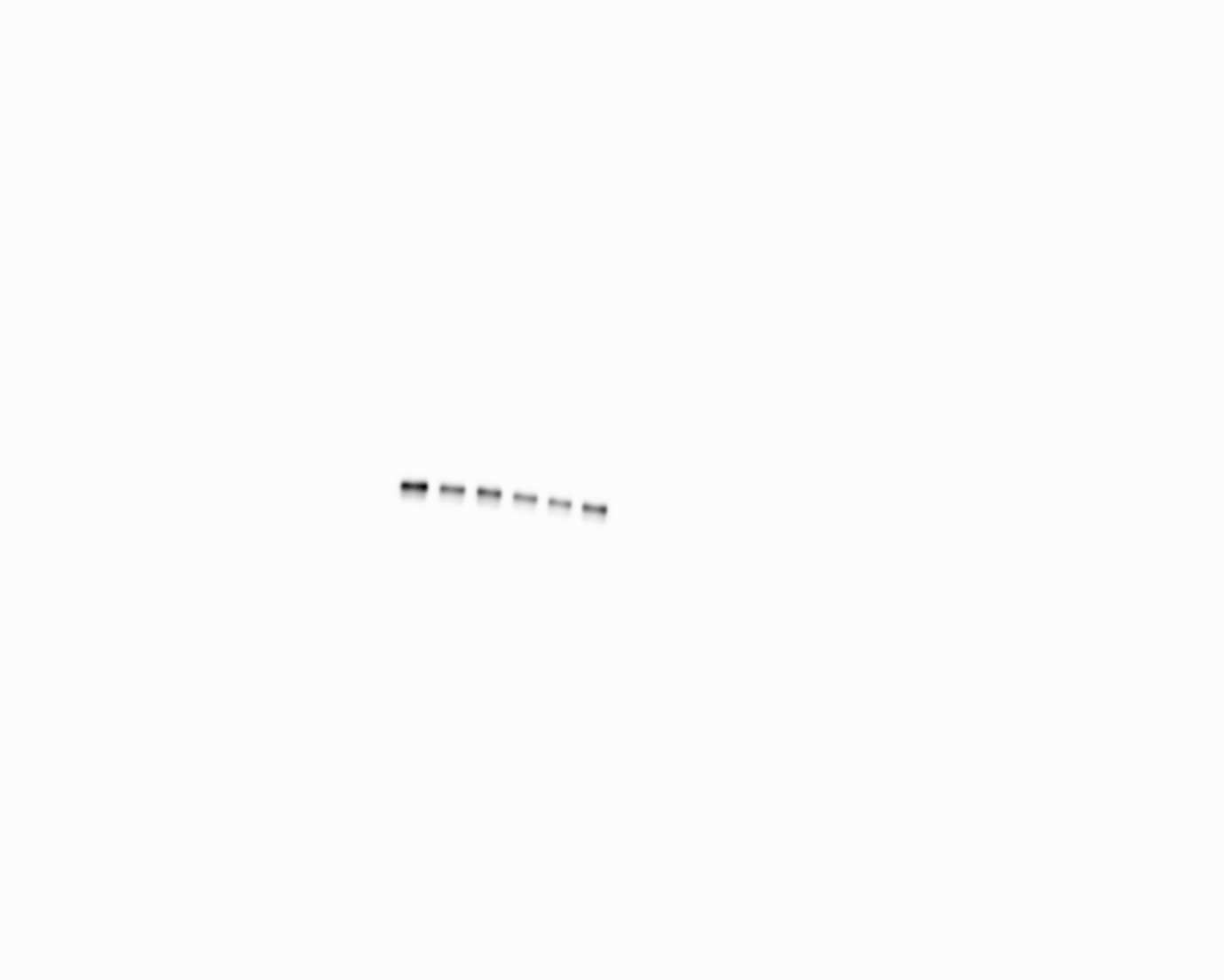

Supplement: Figure 7—source data 1. [file elife-92757-fig7-data1.zip › Fig 7 A-C source data 1/10-20 IP 1/IGG.tif]

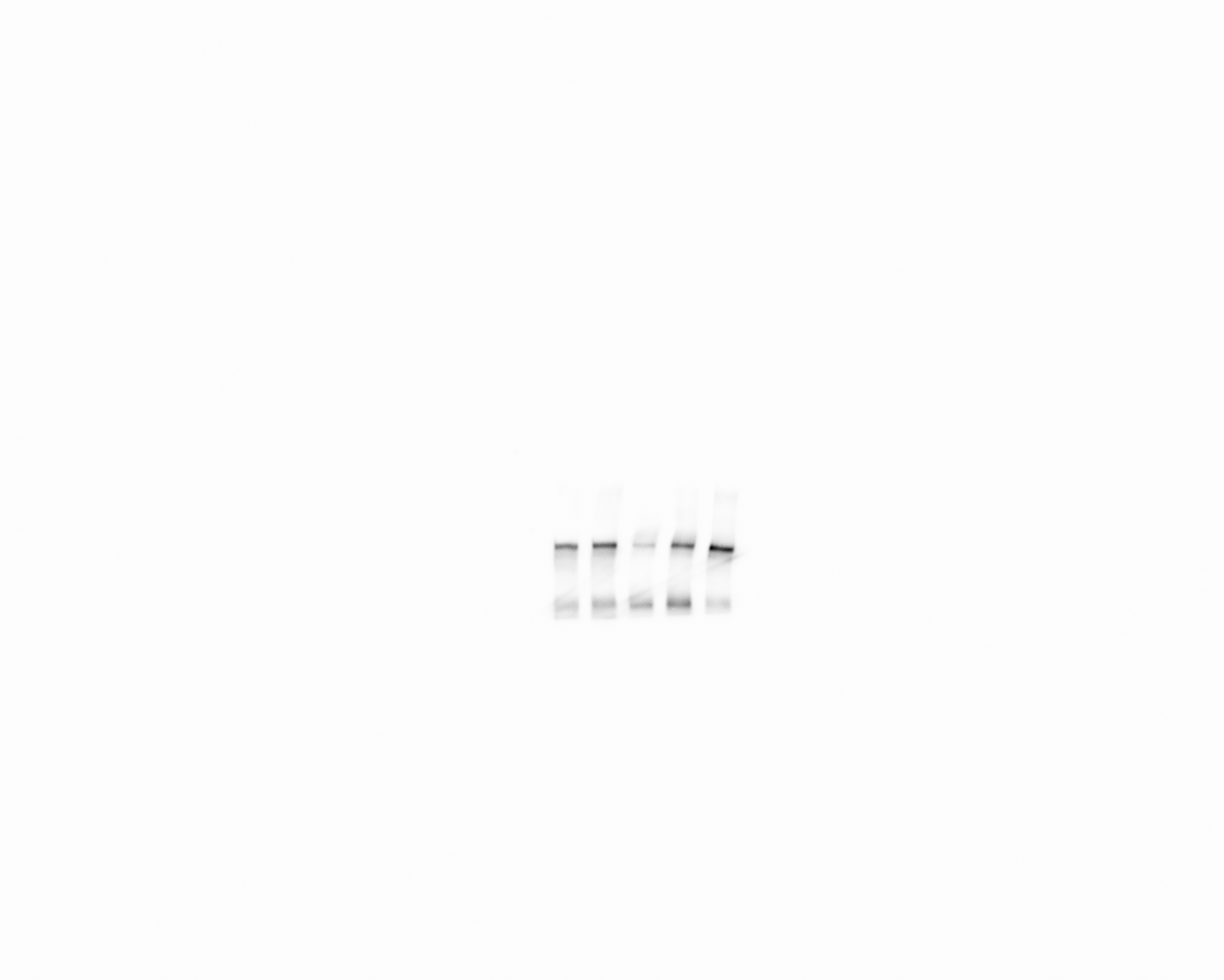

Supplement: Figure 7—source data 1. [file elife-92757-fig7-data1.zip › Fig 7 A-C source data 1/10-20 IP 1/IP robo.tif]

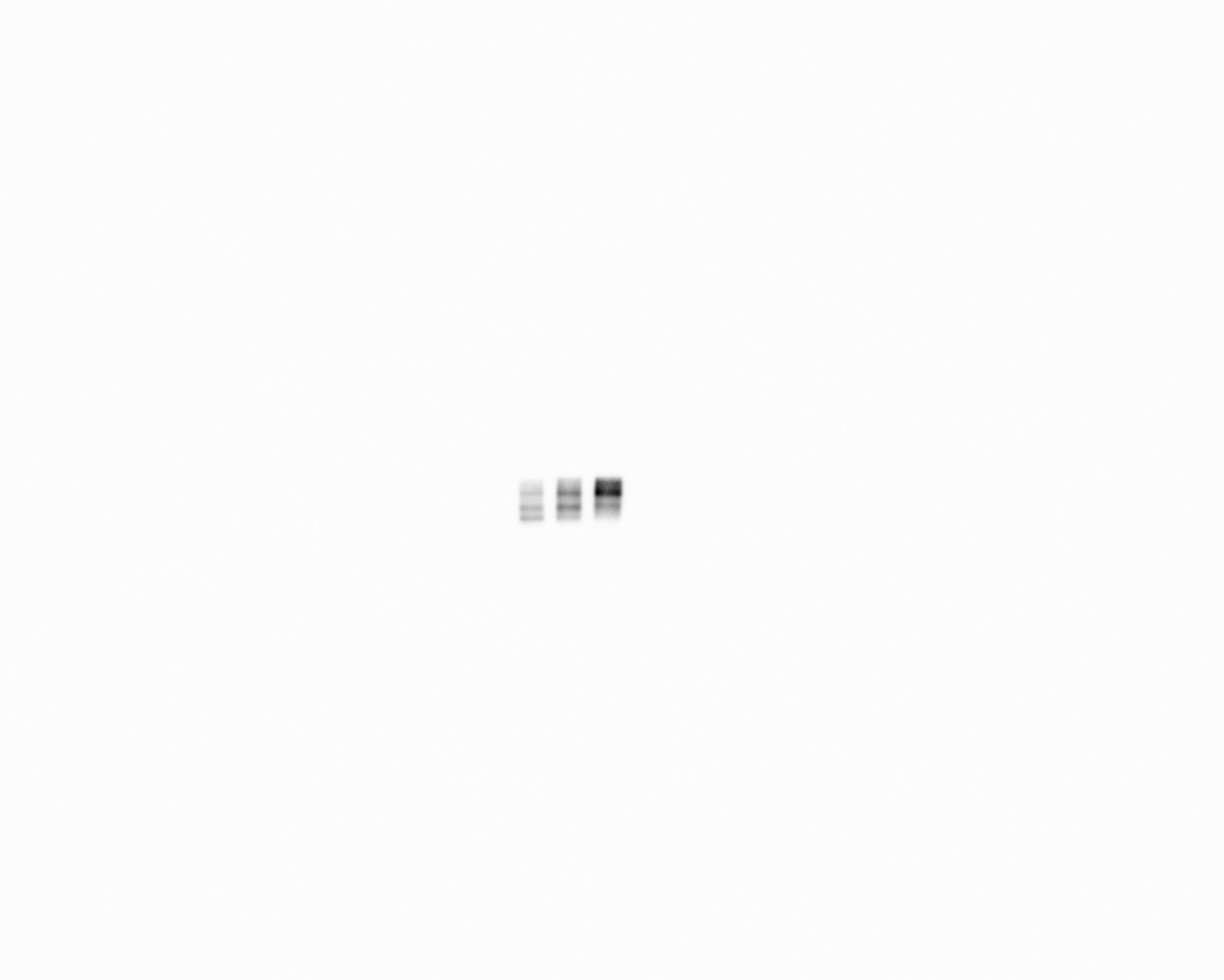

Supplement: Figure 7—source data 1. [file elife-92757-fig7-data1.zip › Fig 7 A-C source data 1/10-20 IP 1/IP comm.tif]

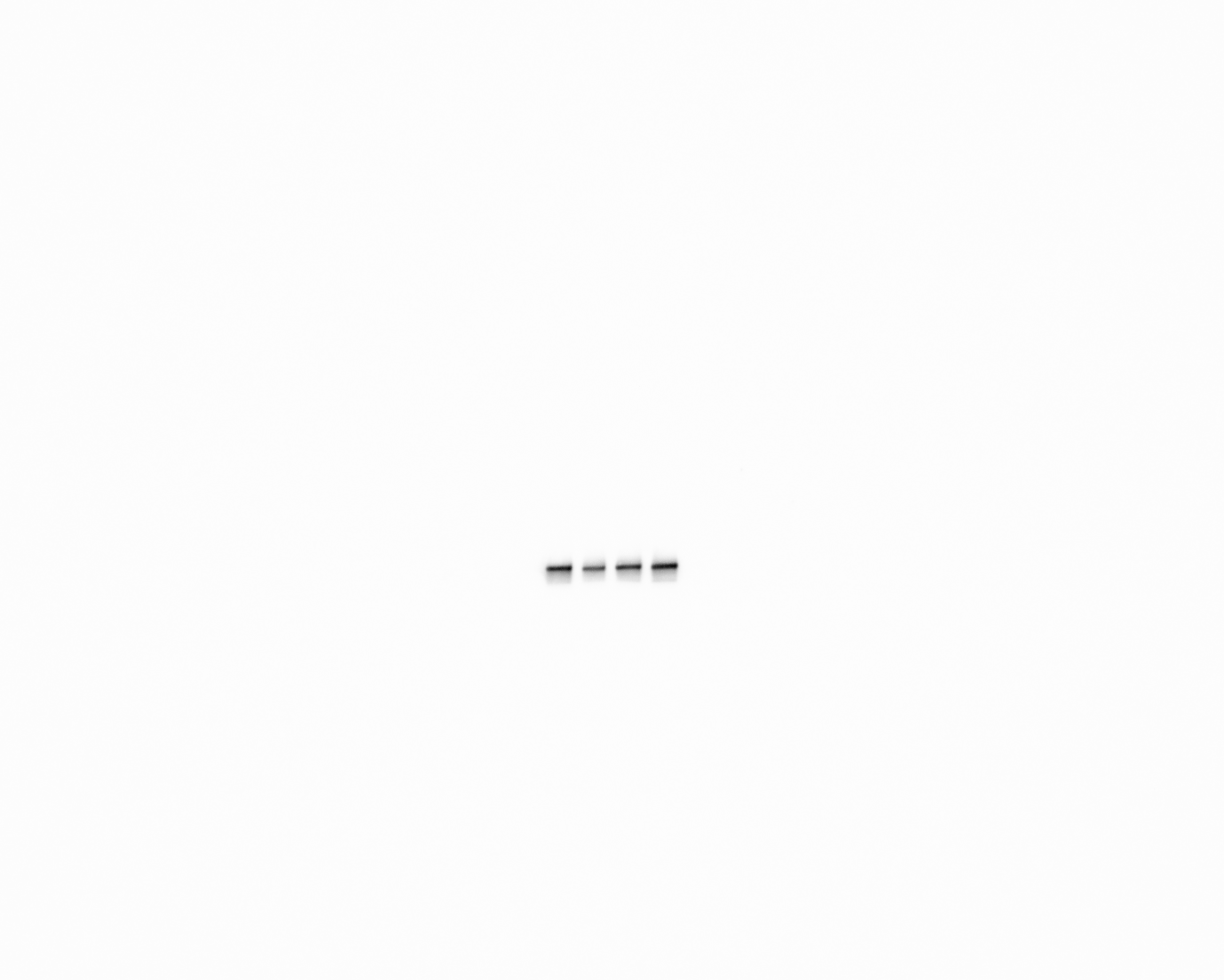

Supplement: Figure 7—source data 1. [file elife-92757-fig7-data1.zip › Fig 7 A-C source data 1/10-20 IP 1/lysate nedd4.tif]

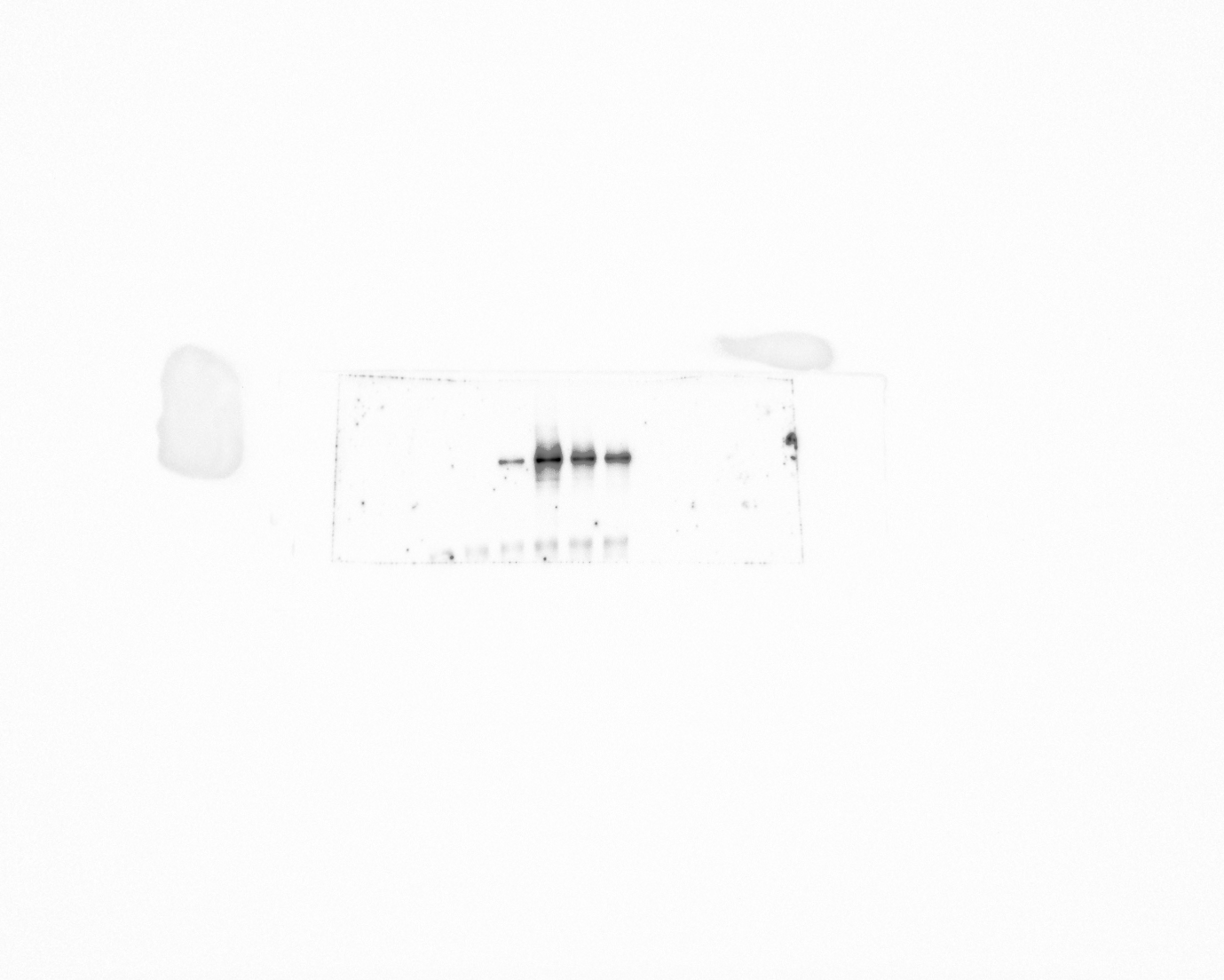

Supplement: Figure 7—source data 1. [file elife-92757-fig7-data1.zip › Fig 7 A-C source data 1/10-20 IP 1/IP nedd4.tif]

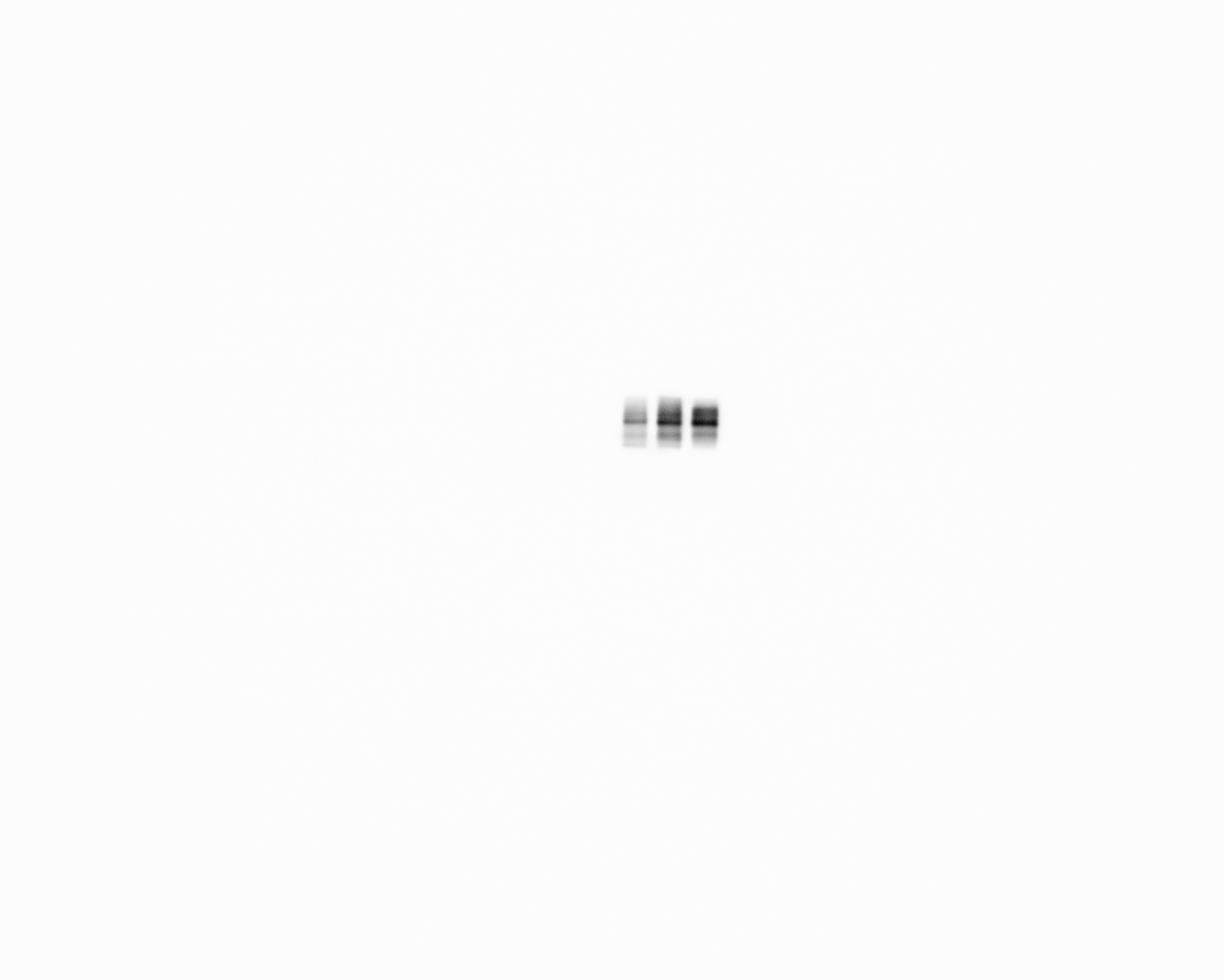

Supplement: Figure 7—source data 1. [file elife-92757-fig7-data1.zip › Fig 7 A-C source data 1/10-20 IP 1/lysate comm.tif]

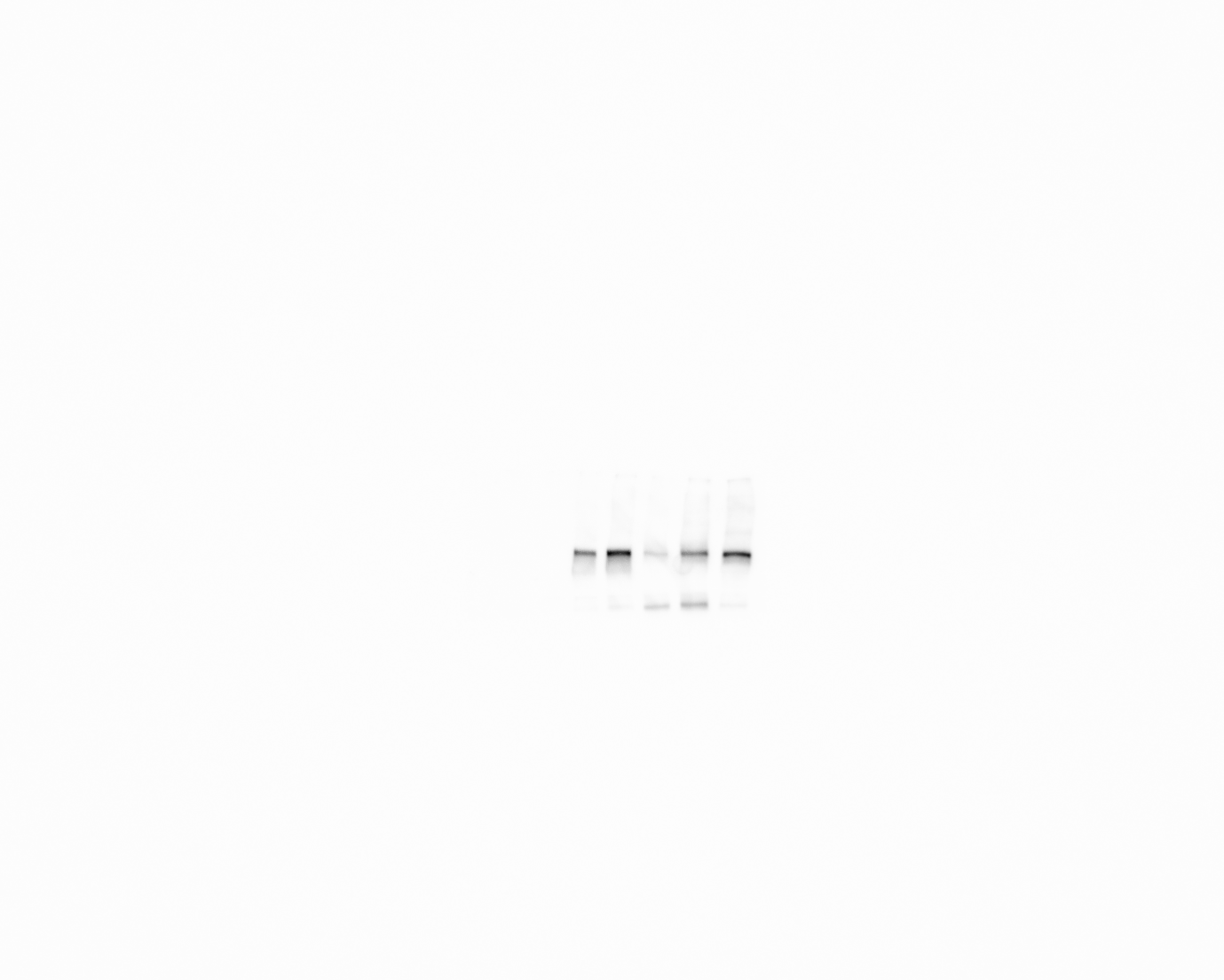

Supplement: Figure 7—source data 1. [file elife-92757-fig7-data1.zip › Fig 7 A-C source data 1/10-20 IP 1/lysate robo.tif]

Oct 20 IP 1

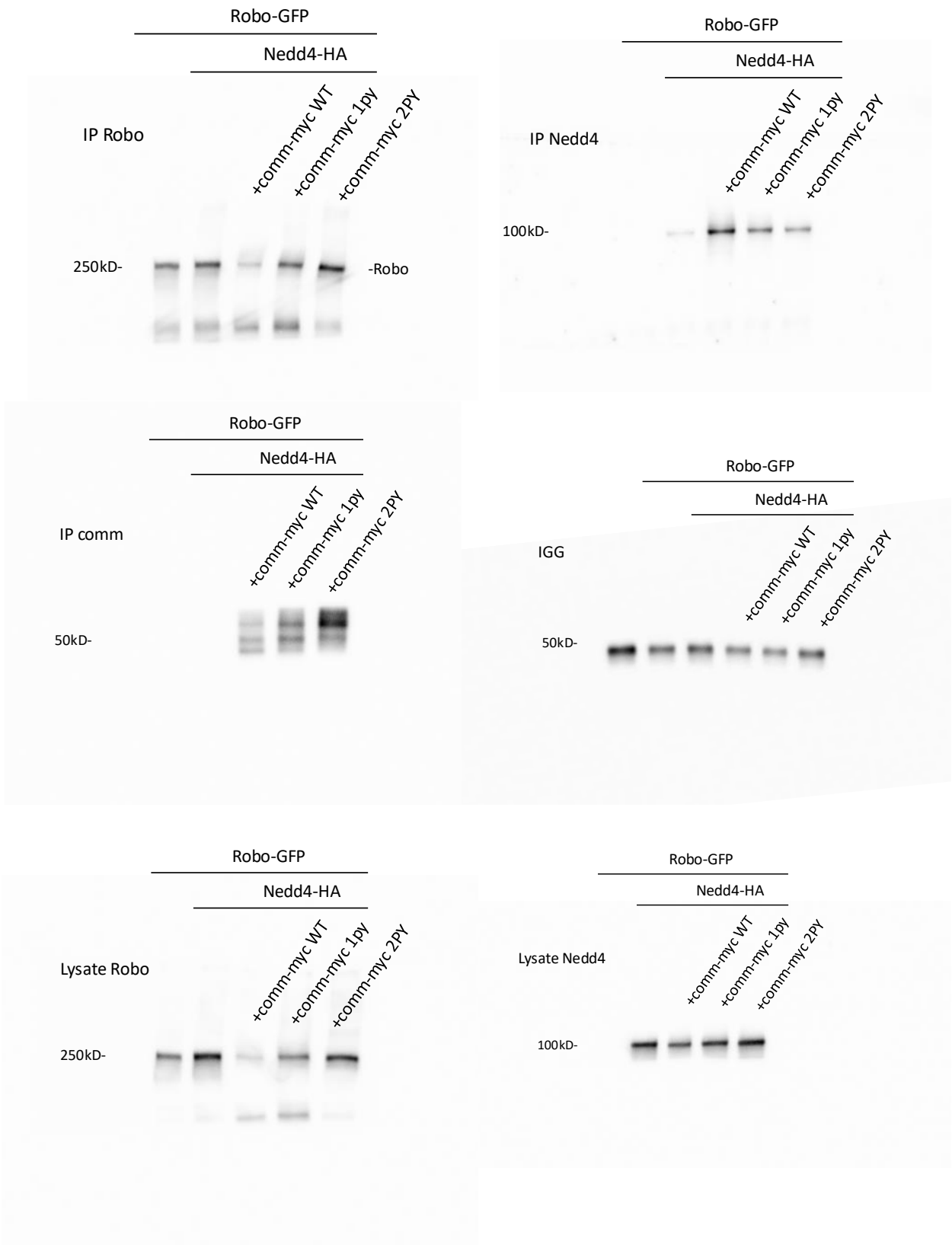

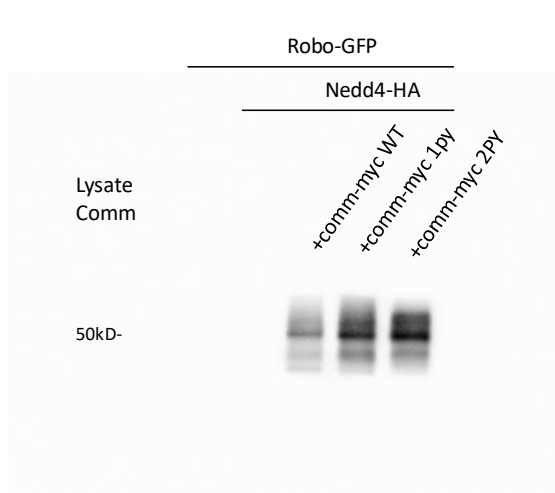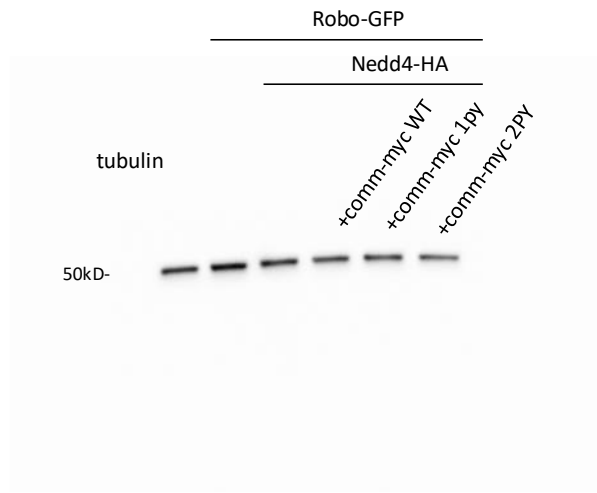

March 9 IP 2

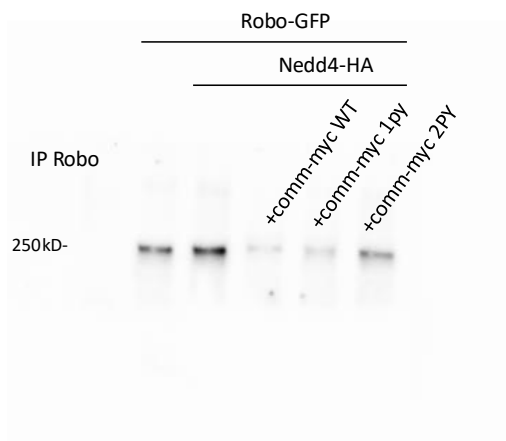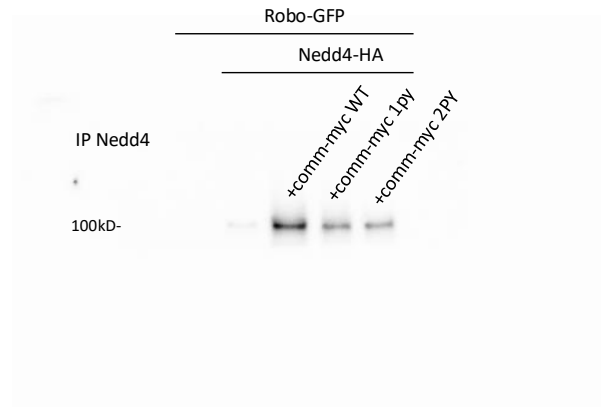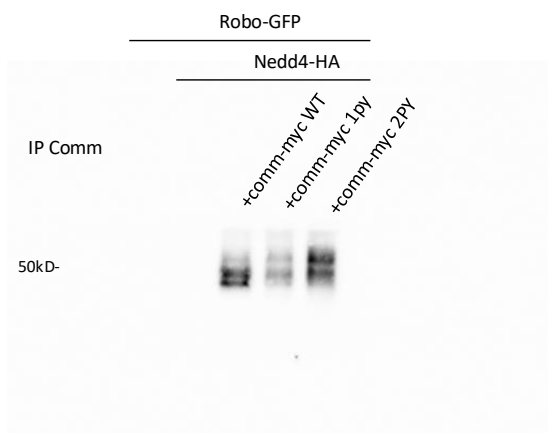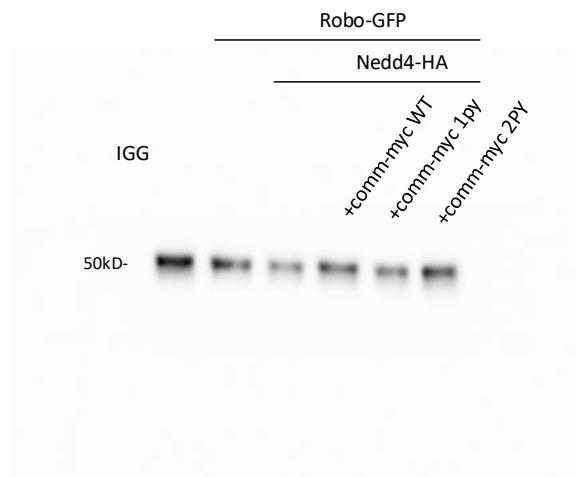

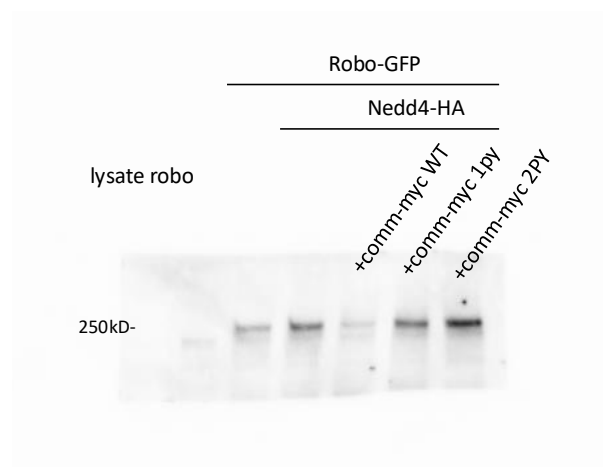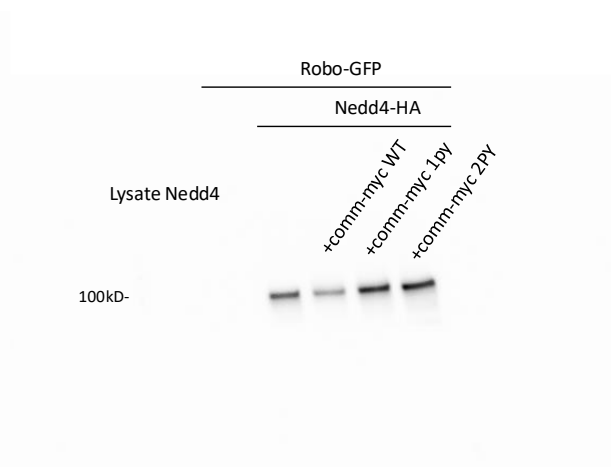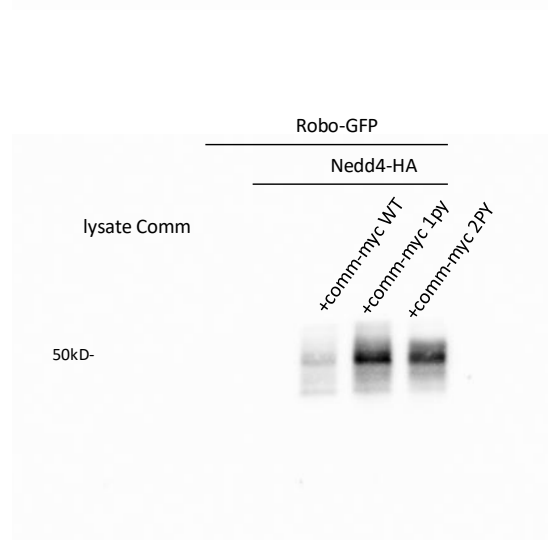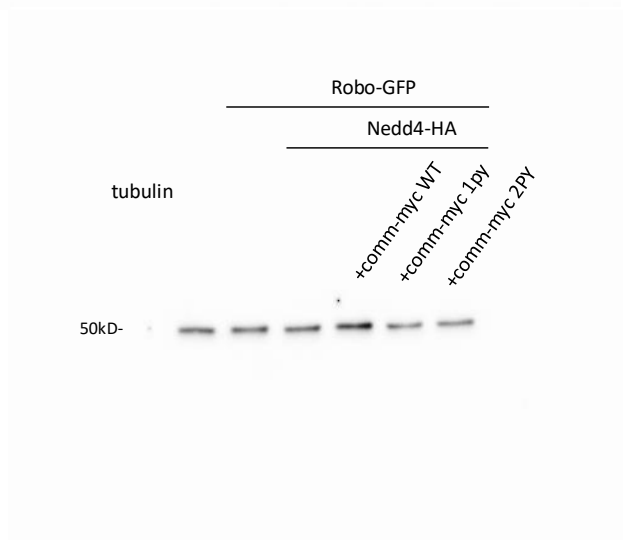

Jun 8 IP 3

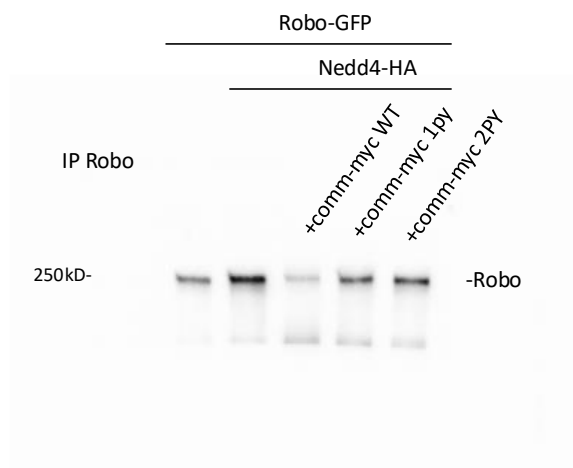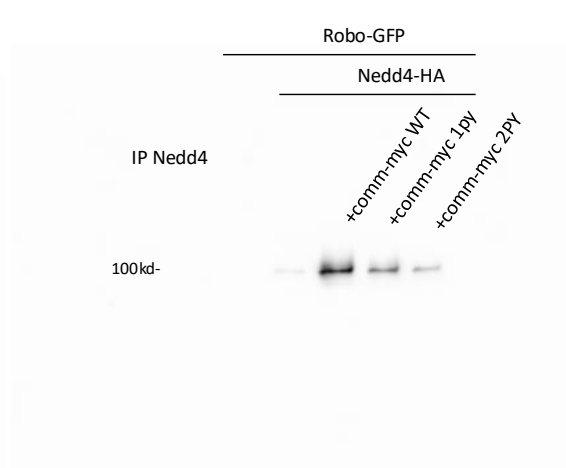

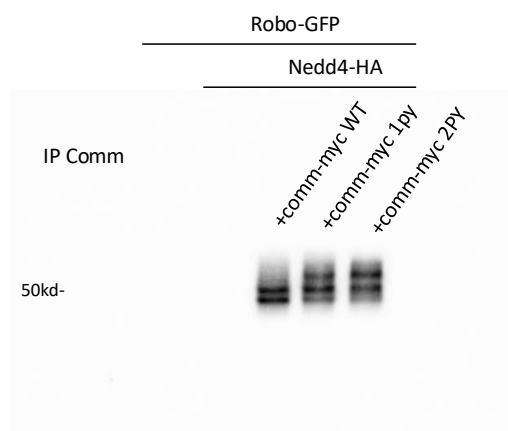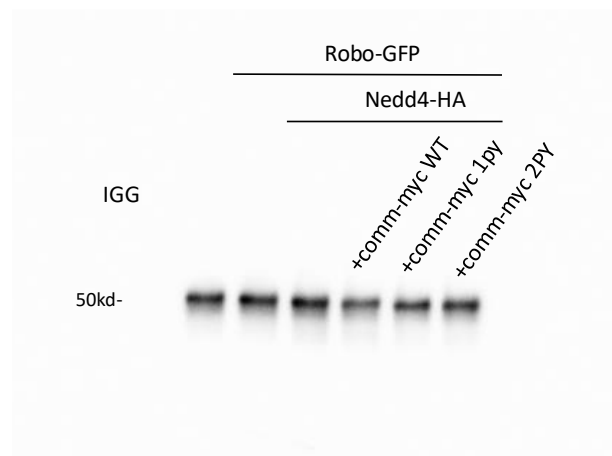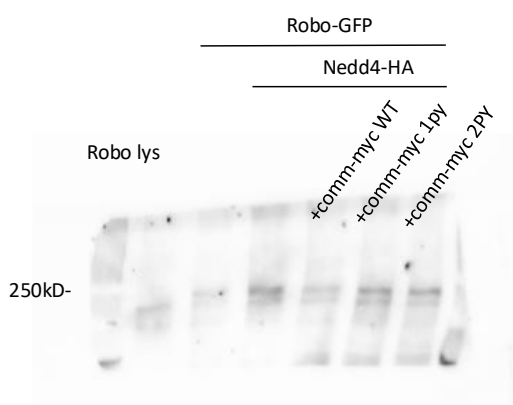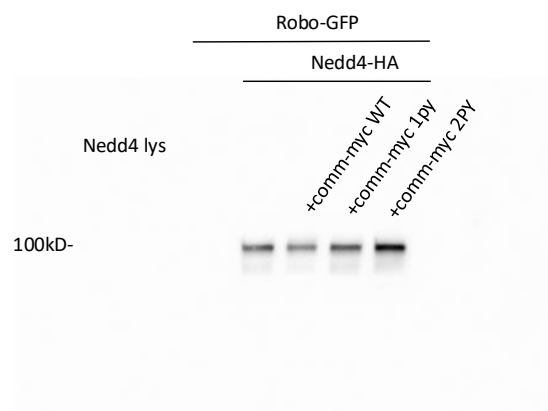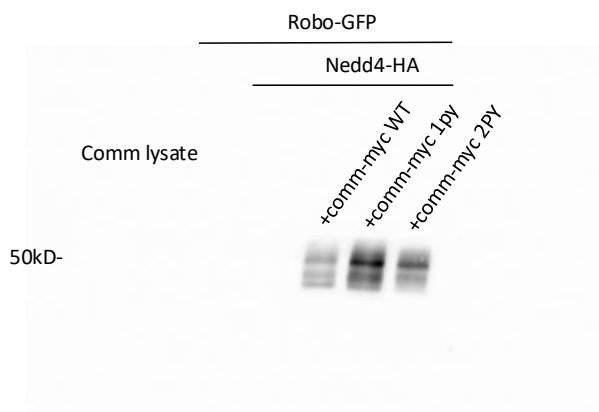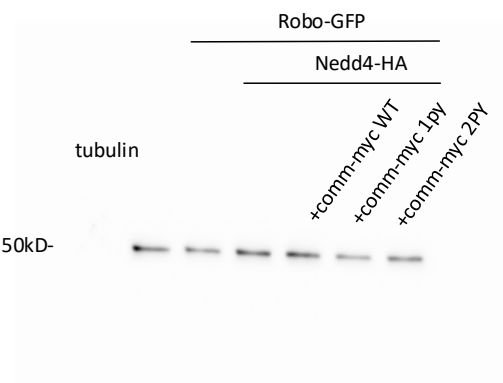

Supplement: Figure 7—source data 2. [file elife-92757-fig7-data2.zip › fig 7 A-C source data 2/Fig 7 A-C labelled blots.pdf]

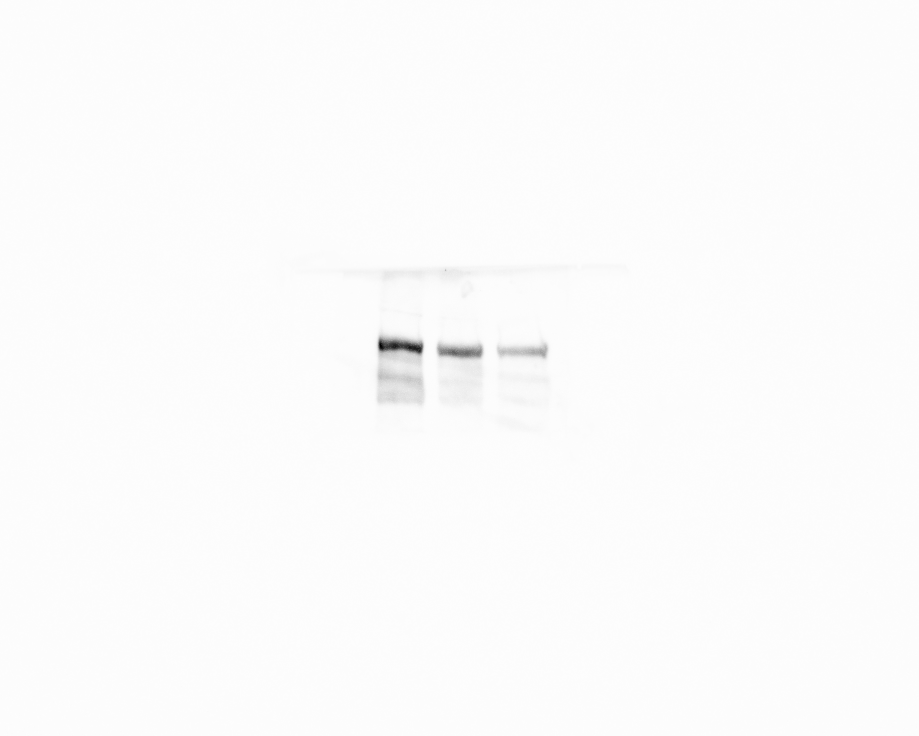

Supplement: Figure 7—source data 3. [file elife-92757-fig7-data3.zip › Figure 7 D-F source data 1/4-15 blot 1/Robo raw.tif]

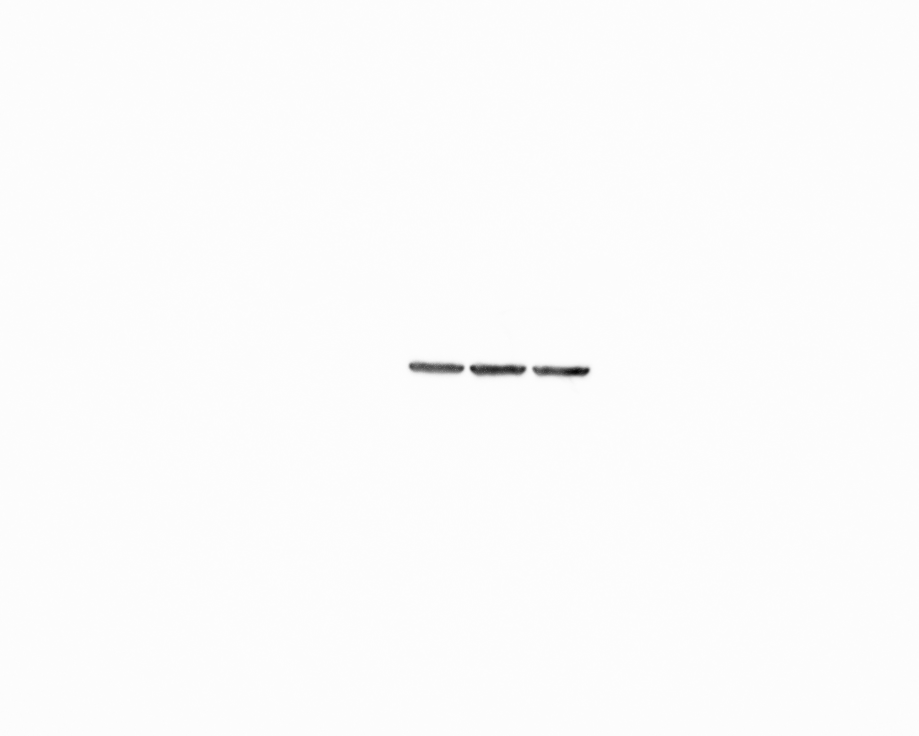

Supplement: Figure 7—source data 3. [file elife-92757-fig7-data3.zip › Figure 7 D-F source data 1/4-15 blot 1/tubulin raw.tif]

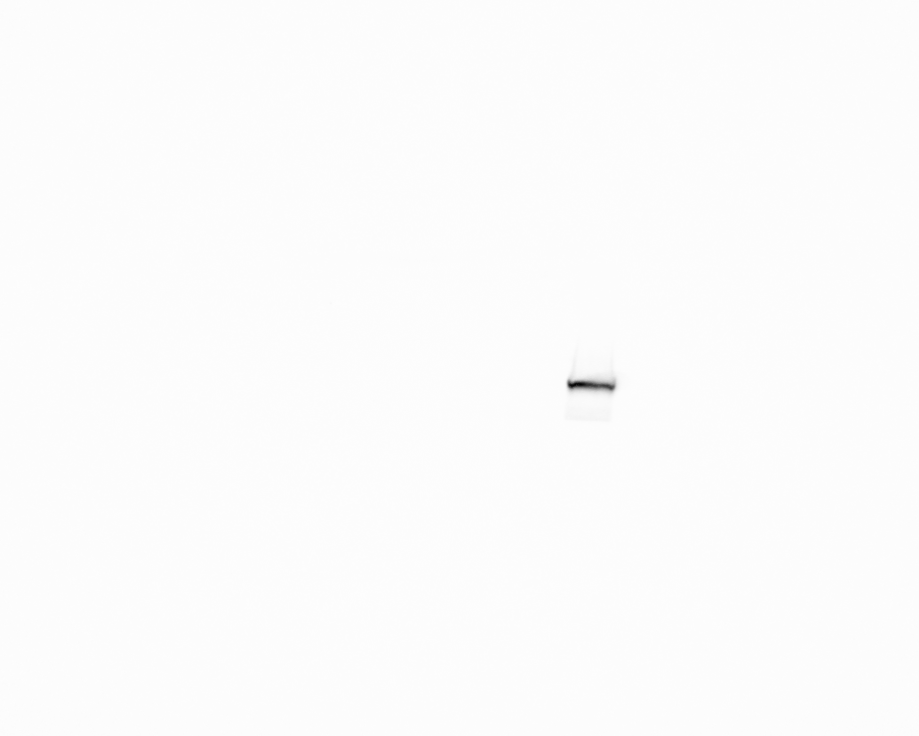

Supplement: Figure 7—source data 3. [file elife-92757-fig7-data3.zip › Figure 7 D-F source data 1/4-15 blot 1/Nedd4 raw.tif]

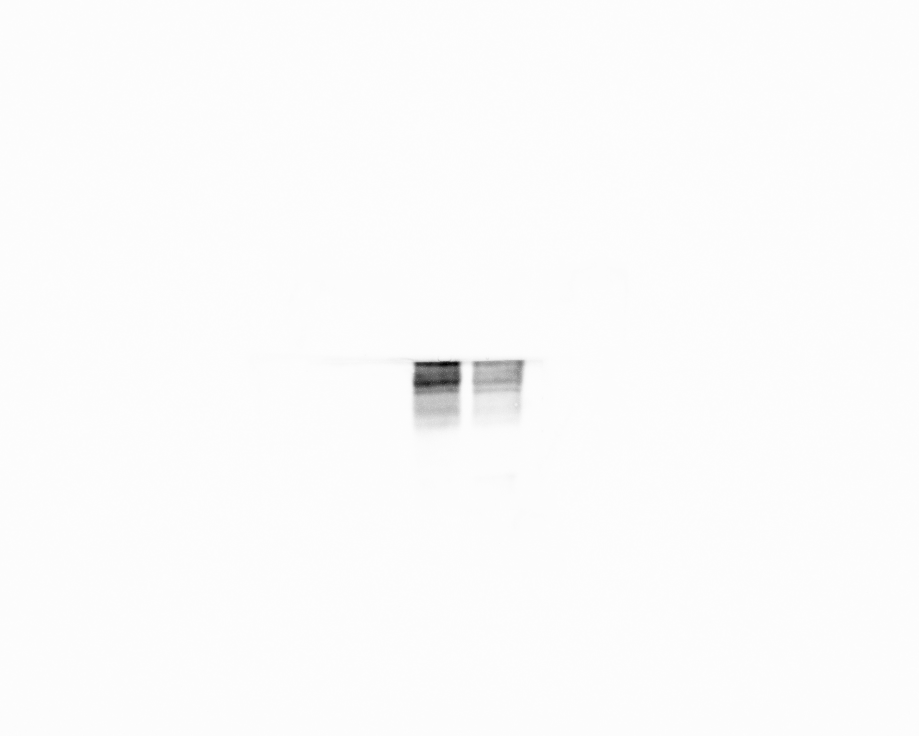

Supplement: Figure 7—source data 3. [file elife-92757-fig7-data3.zip › Figure 7 D-F source data 1/4-15 blot 1/comm raw.tif]

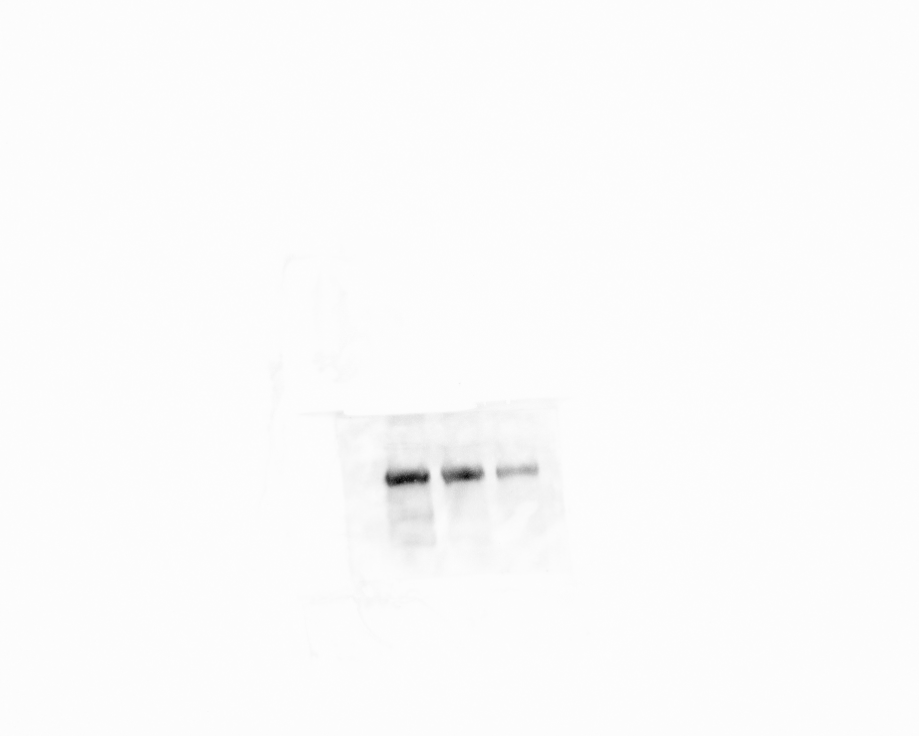

Supplement: Figure 7—source data 3. [file elife-92757-fig7-data3.zip › Figure 7 D-F source data 1/4-18 blot 3/Robo raw.tif]

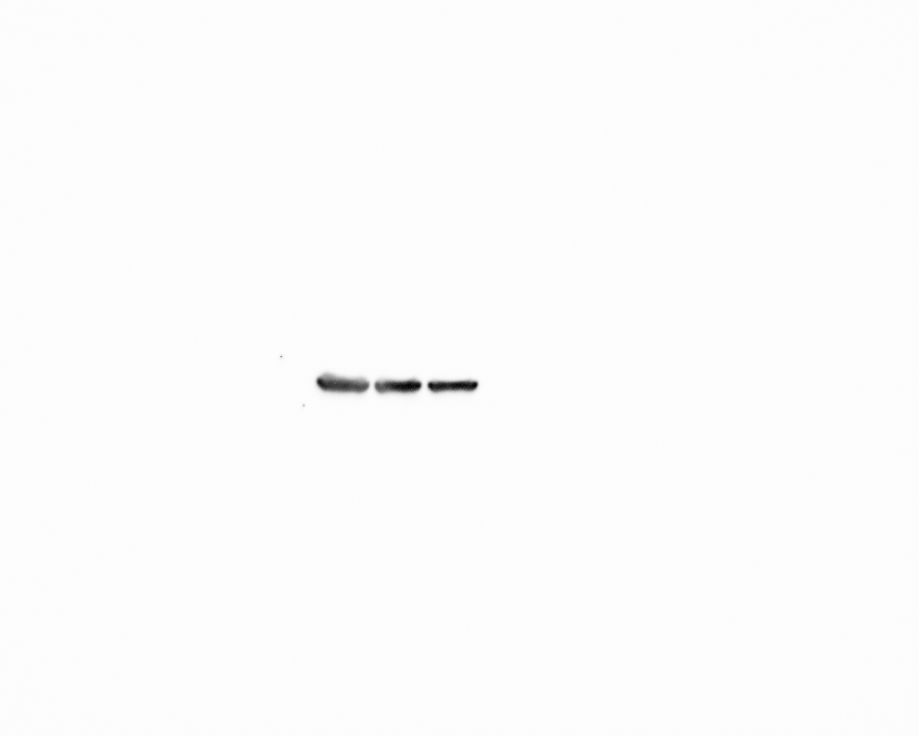

Supplement: Figure 7—source data 3. [file elife-92757-fig7-data3.zip › Figure 7 D-F source data 1/4-18 blot 3/tubulin raw.tif]

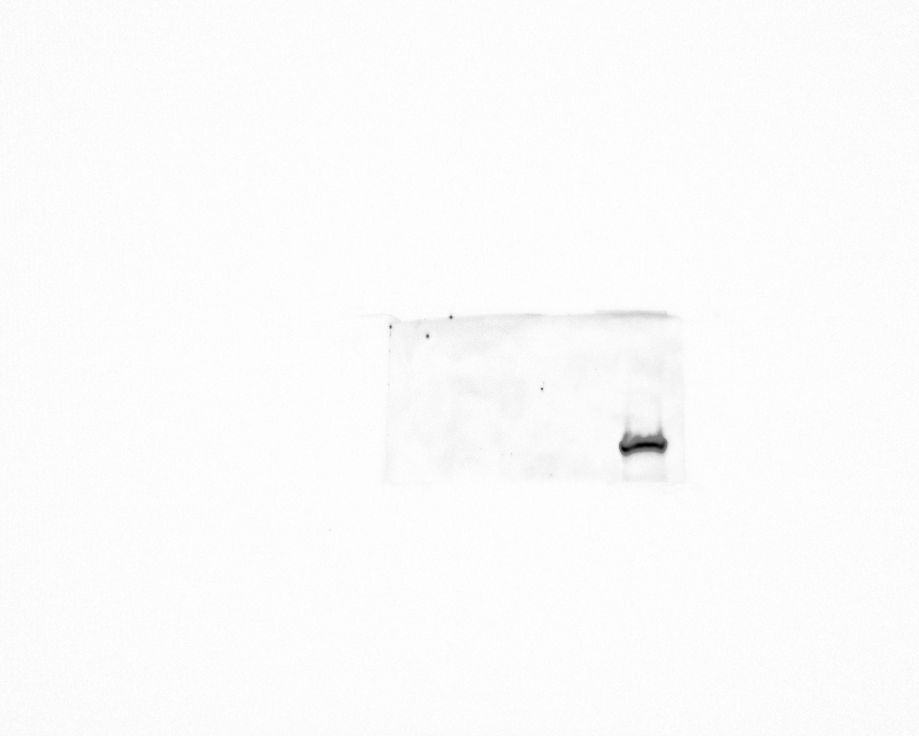

Supplement: Figure 7—source data 3. [file elife-92757-fig7-data3.zip › Figure 7 D-F source data 1/4-18 blot 3/nedd4 raw.tif]

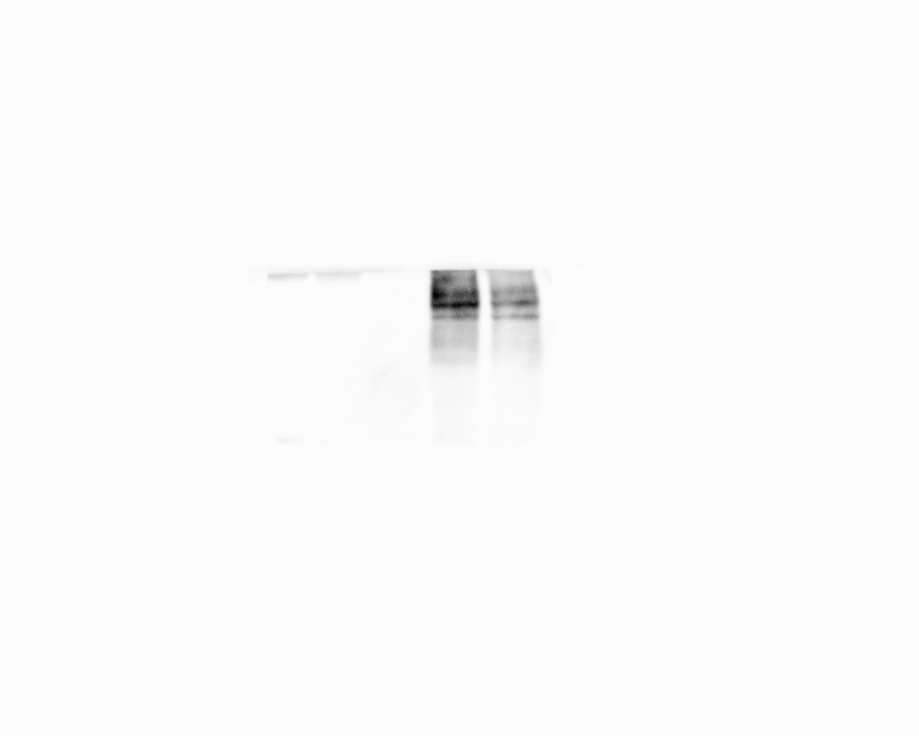

Supplement: Figure 7—source data 3. [file elife-92757-fig7-data3.zip › Figure 7 D-F source data 1/4-18 blot 3/comm raw.tif]

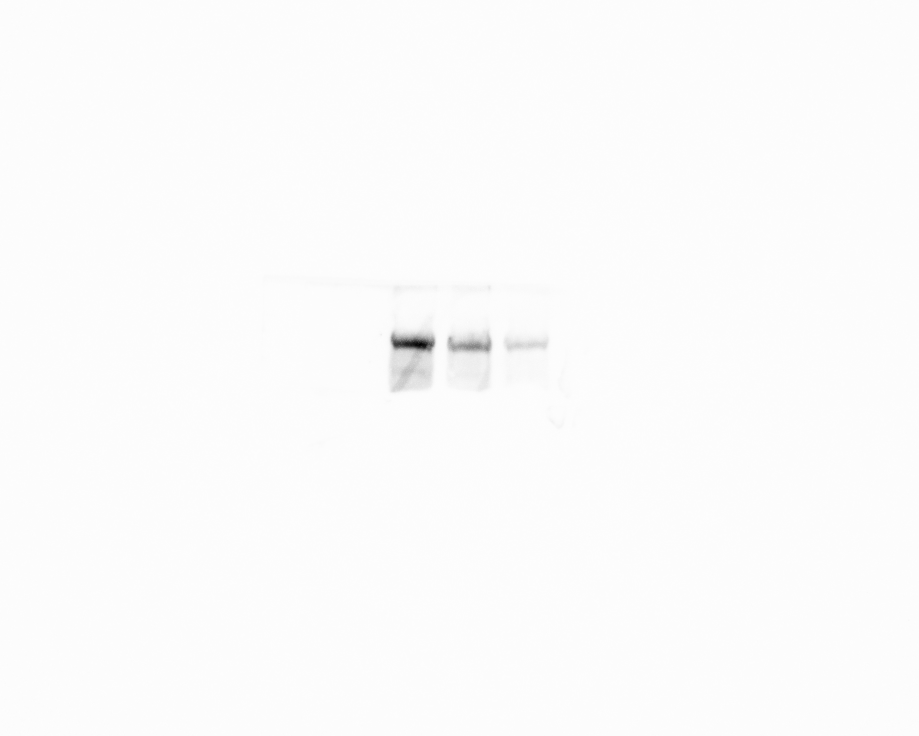

Supplement: Figure 7—source data 3. [file elife-92757-fig7-data3.zip › Figure 7 D-F source data 1/4-17 blot 2/robo raw.tif]

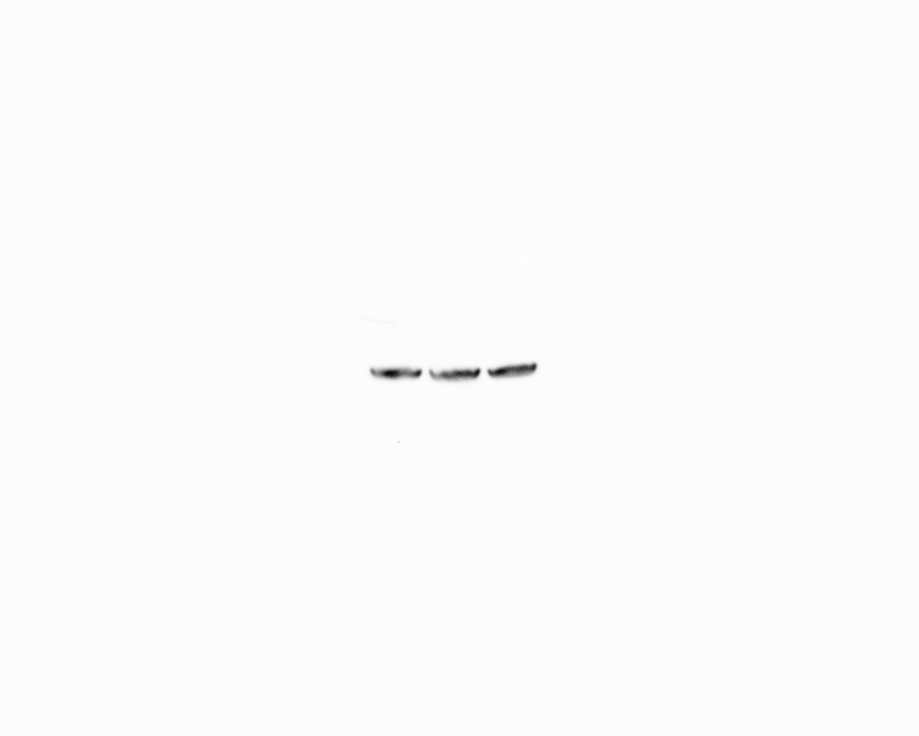

Supplement: Figure 7—source data 3. [file elife-92757-fig7-data3.zip › Figure 7 D-F source data 1/4-17 blot 2/tubulin raw.tif]

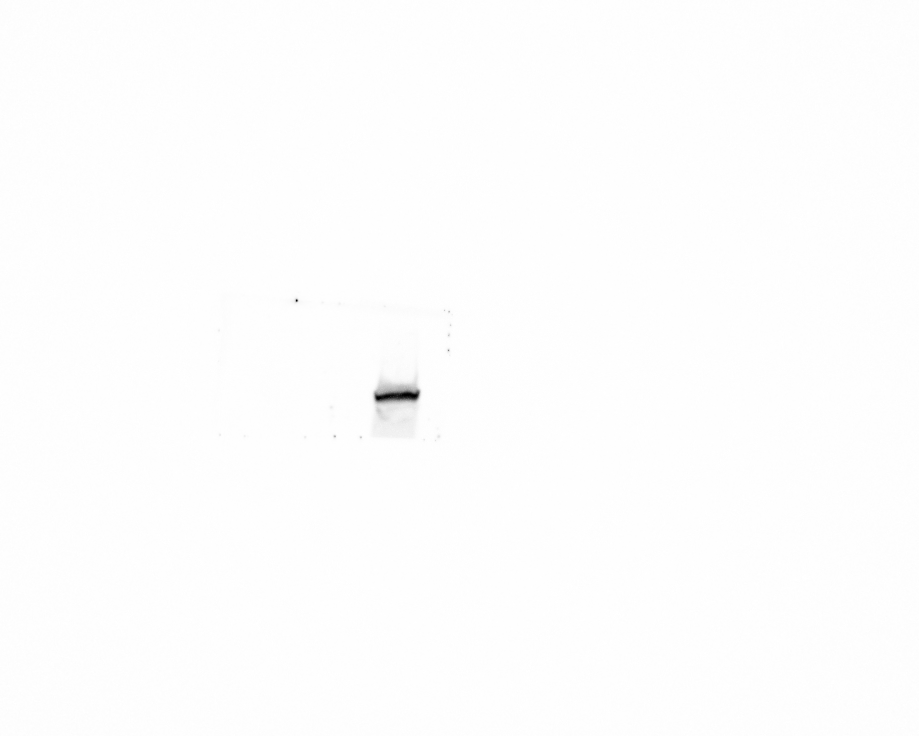

Supplement: Figure 7—source data 3. [file elife-92757-fig7-data3.zip › Figure 7 D-F source data 1/4-17 blot 2/Nedd4 raw.tif]

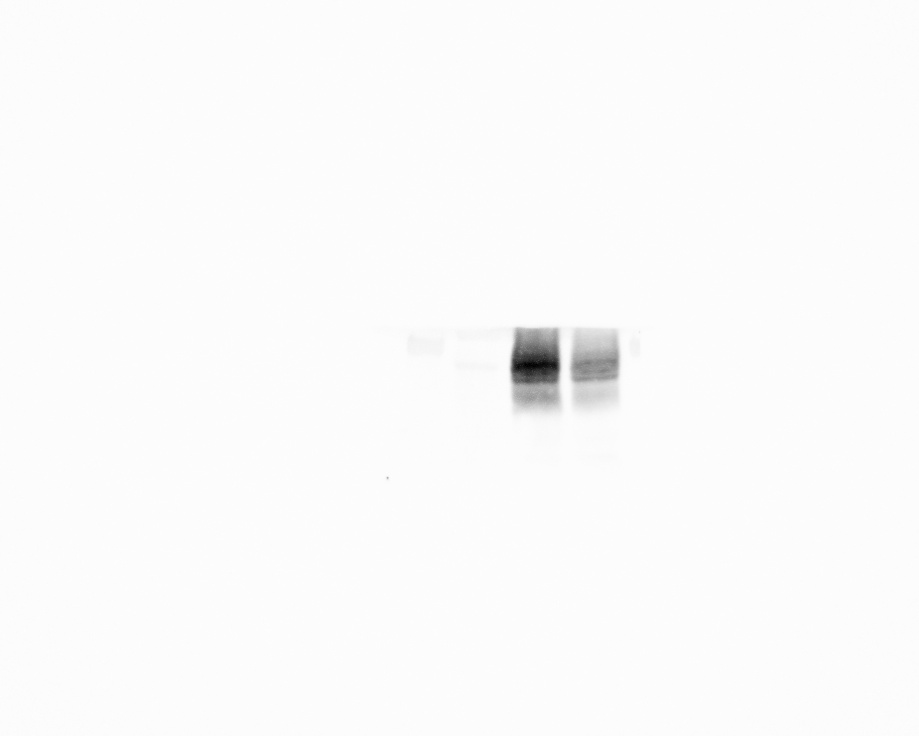

Supplement: Figure 7—source data 3. [file elife-92757-fig7-data3.zip › Figure 7 D-F source data 1/4-17 blot 2/Comm raw.tif]

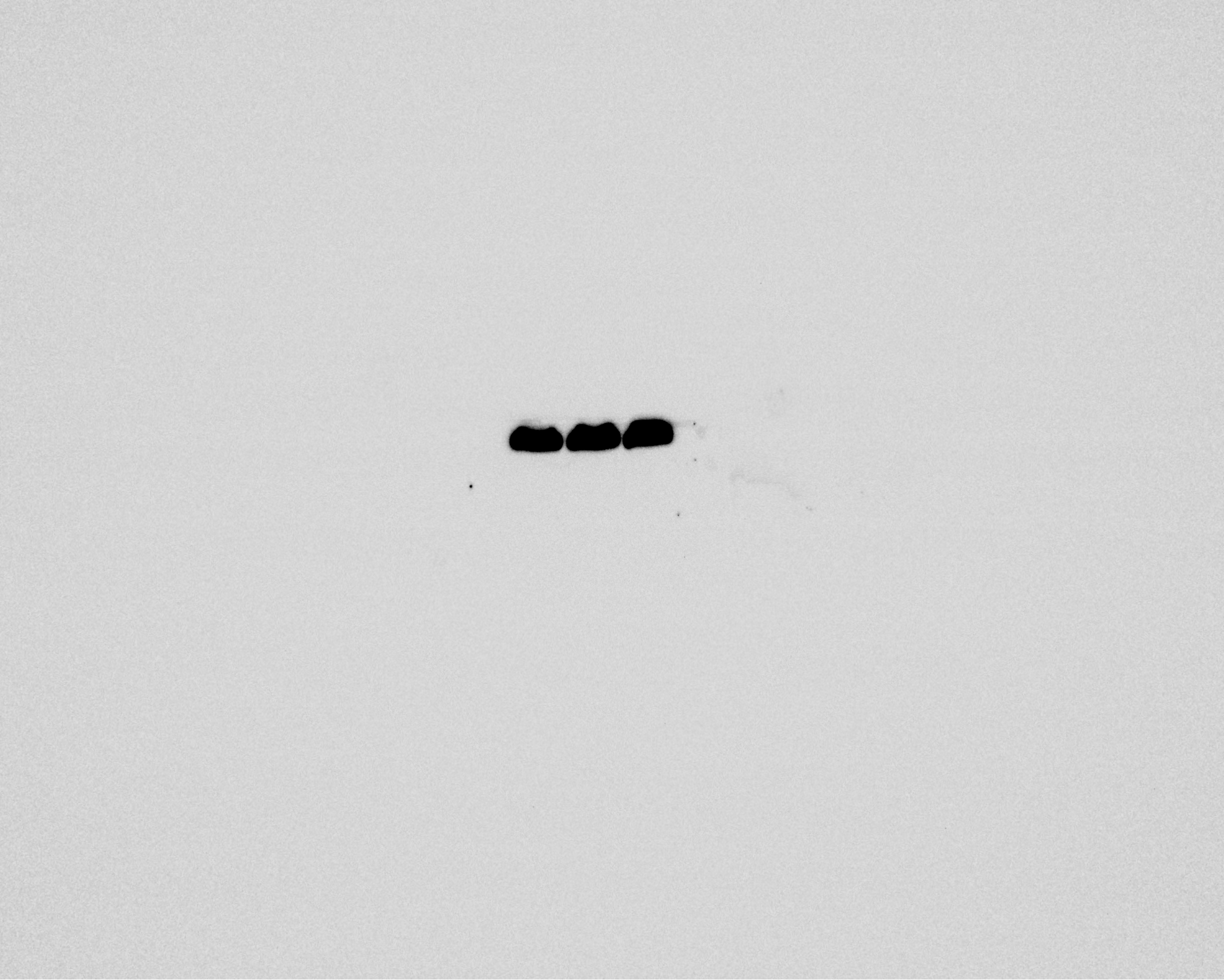

Supplement: Figure 7—figure supplement 1—source data 1. [file elife-92757-fig7-figsupp1-data1.zip › Figure 7 supplement 1 source data 1/tubulin.tif]

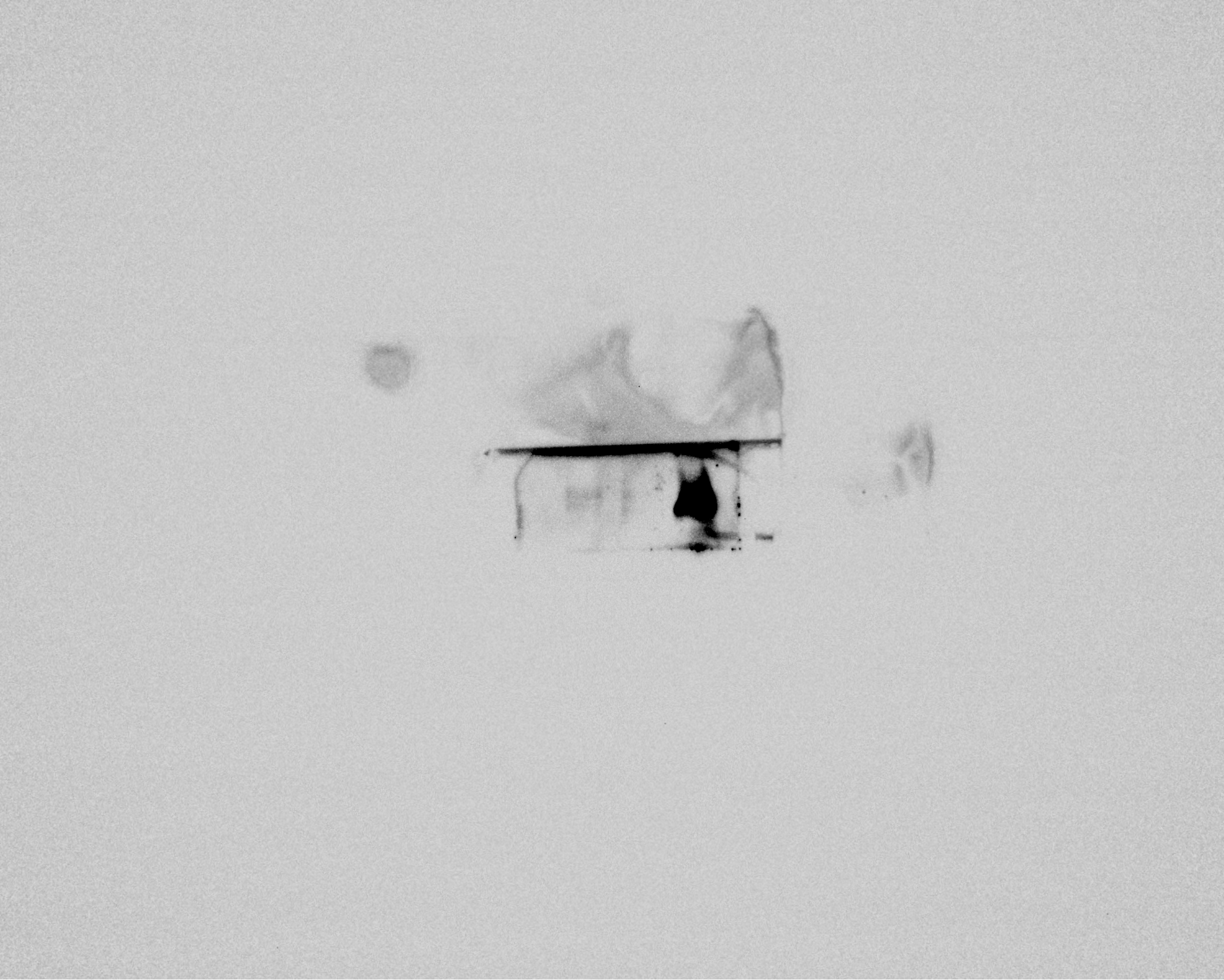

Supplement: Figure 7—figure supplement 1—source data 1. [file elife-92757-fig7-figsupp1-data1.zip › Figure 7 supplement 1 source data 1/nedd4 lysate.tif]

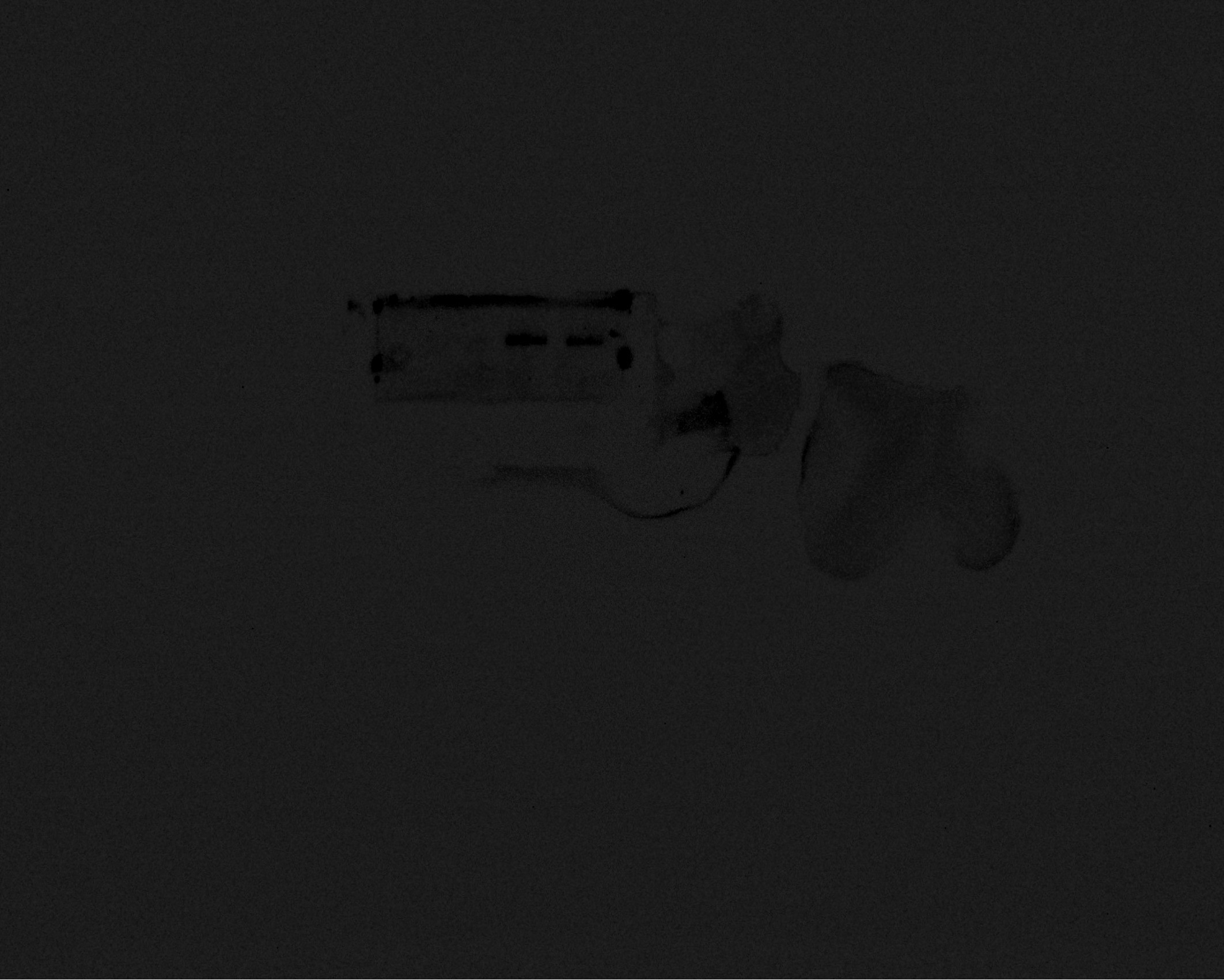

Supplement: Figure 7—figure supplement 1—source data 1. [file elife-92757-fig7-figsupp1-data1.zip › Figure 7 supplement 1 source data 1/ip robo.tif]

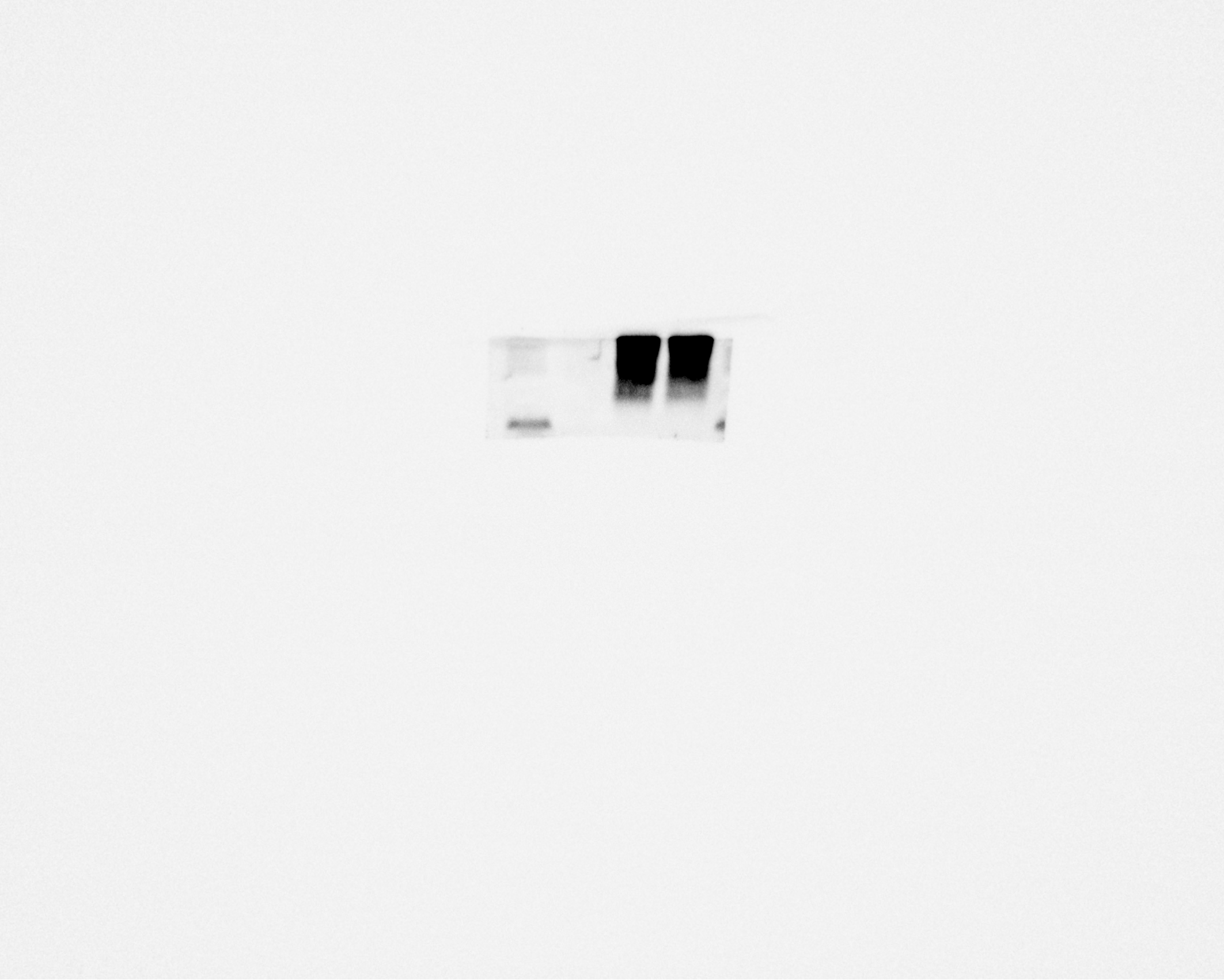

Supplement: Figure 7—figure supplement 1—source data 1. [file elife-92757-fig7-figsupp1-data1.zip › Figure 7 supplement 1 source data 1/IP comm.tif]

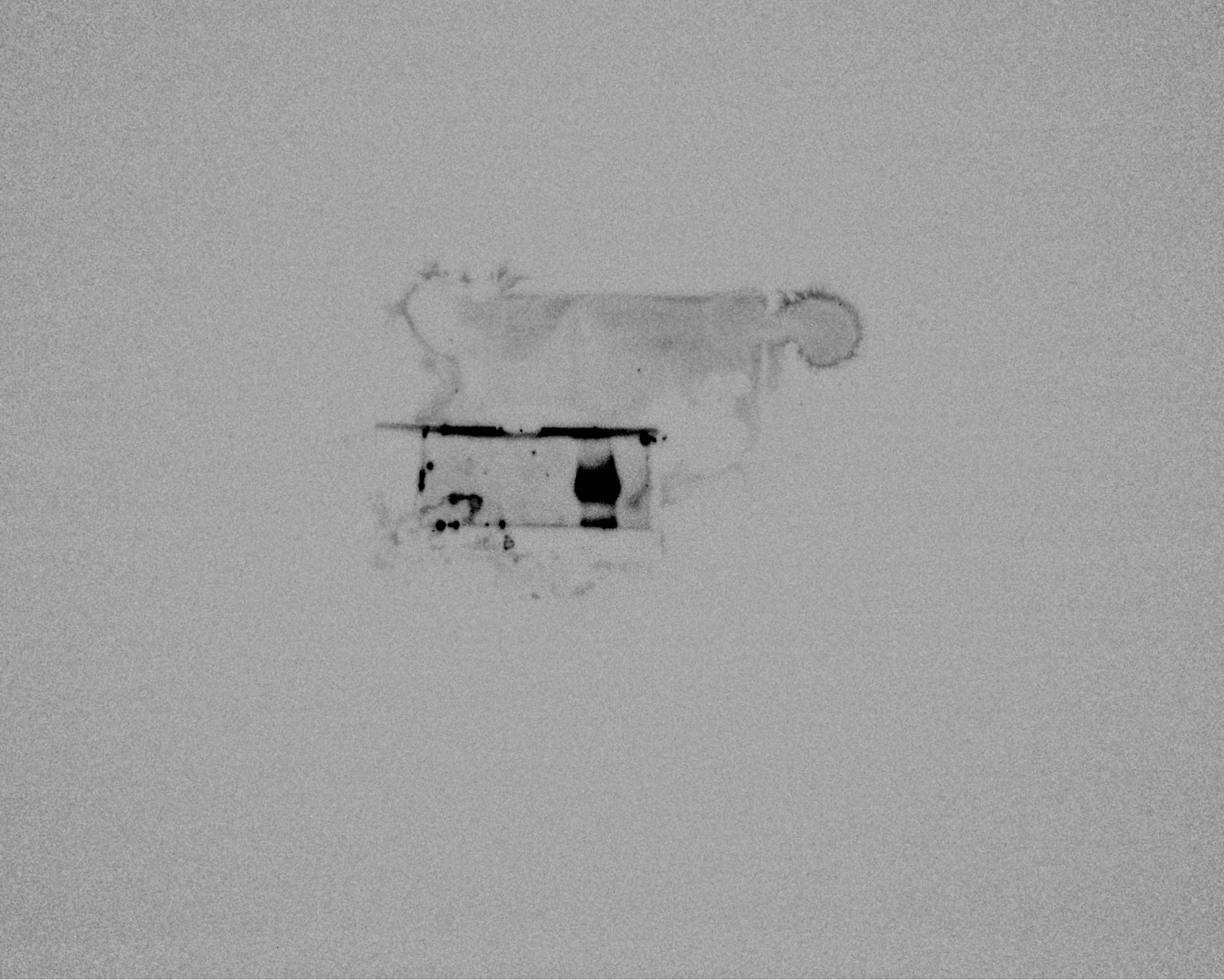

Supplement: Figure 7—figure supplement 1—source data 1. [file elife-92757-fig7-figsupp1-data1.zip › Figure 7 supplement 1 source data 1/IP nedd4.tif]

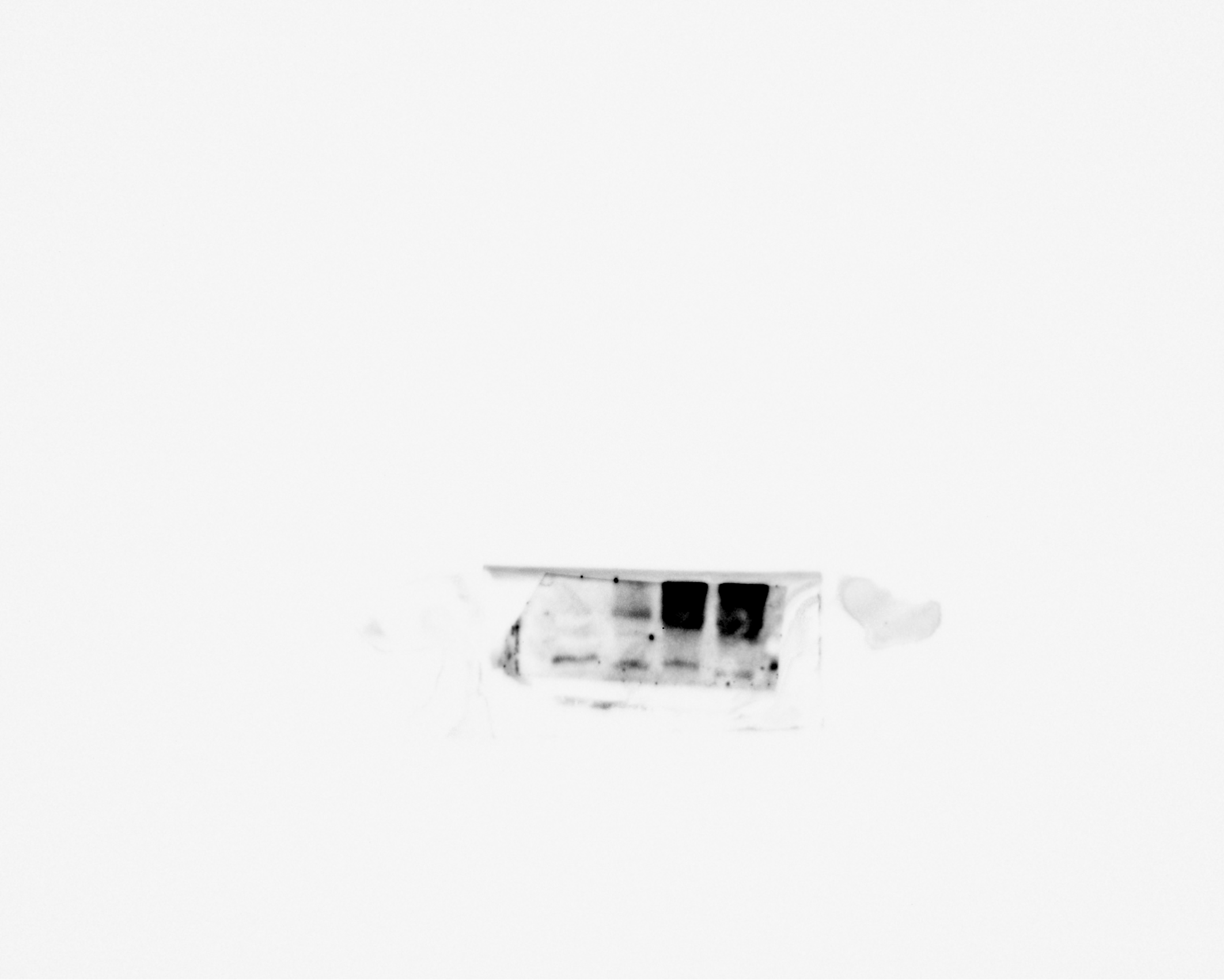

Supplement: Figure 7—figure supplement 1—source data 1. [file elife-92757-fig7-figsupp1-data1.zip › Figure 7 supplement 1 source data 1/lysate comm.tif]

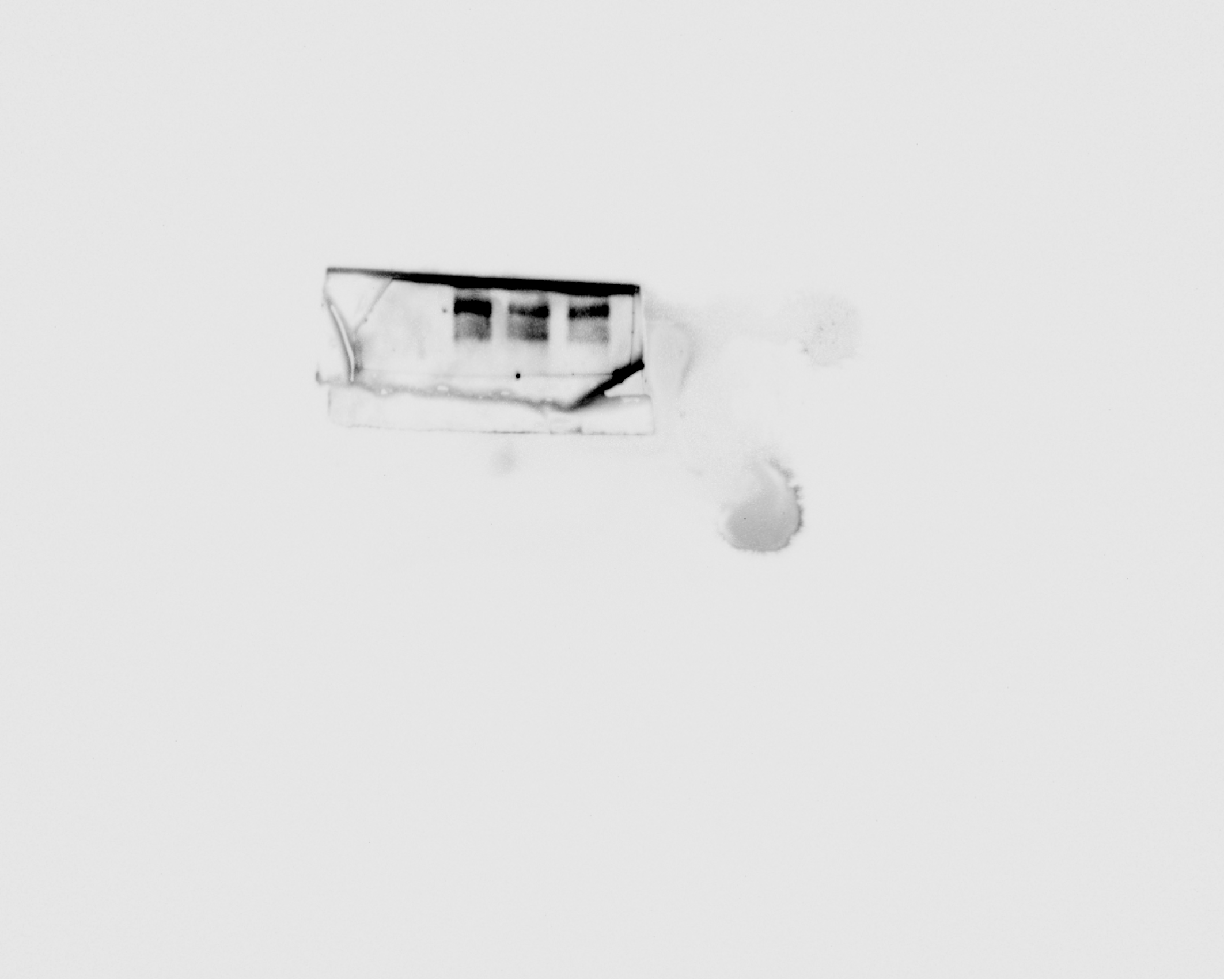

Supplement: Figure 7—figure supplement 1—source data 1. [file elife-92757-fig7-figsupp1-data1.zip › Figure 7 supplement 1 source data 1/lysate robo.tif]

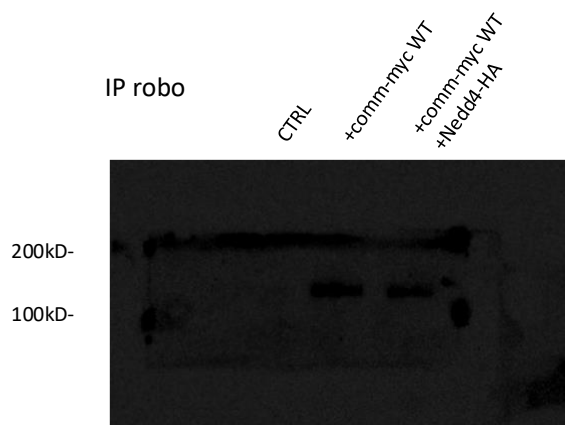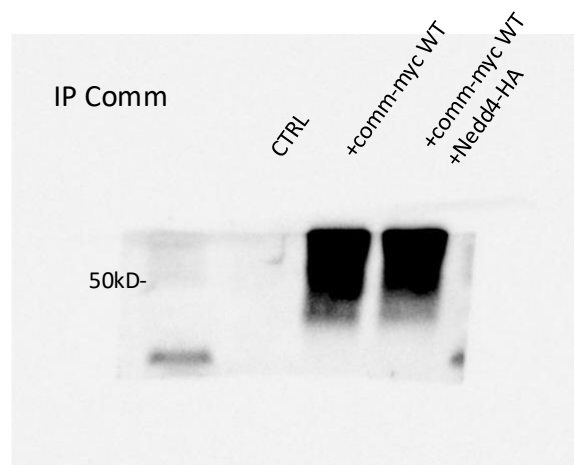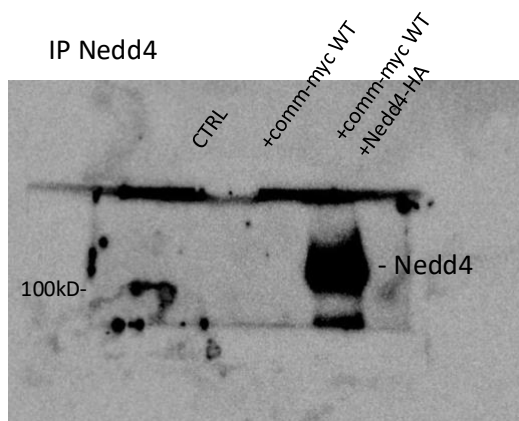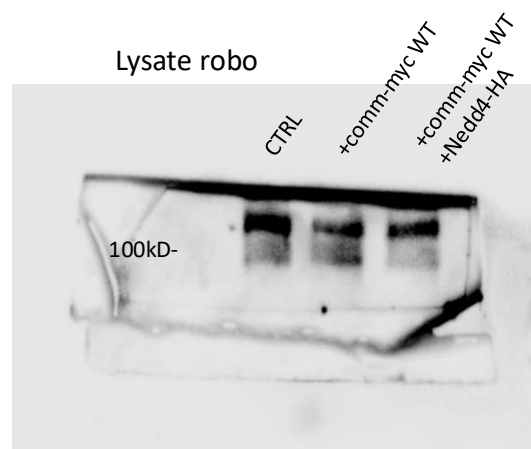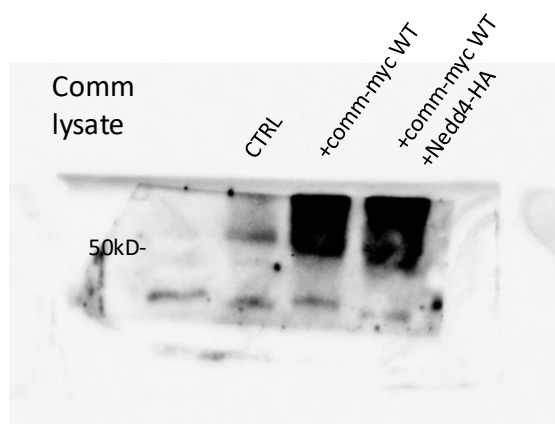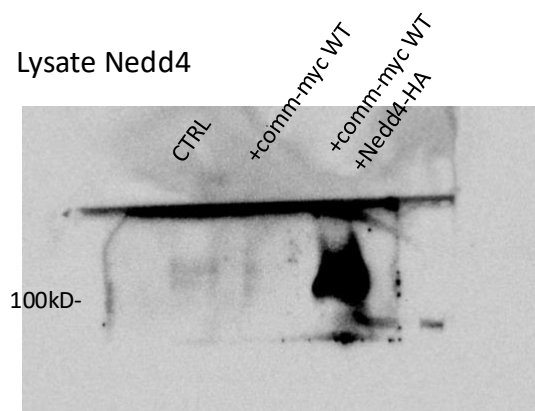

Supplement: Figure 7—figure supplement 1—source data 2. [file elife-92757-fig7-figsupp1-data2.pdf]
