# Supplementary material for: Commissureless acts as a substrate adapter in a conserved Nedd4 E3 ubiquitin ligase pathway to promote axon growth across the midline
Source: eLife. 2025 May 23;13:RP92757. doi: 10.7554/eLife.92757 (PMC12101832; doi:10.7554/eLife.92757)
Supplement: Figure 7—source data 4. [file elife-92757-fig7-data4.zip › Figure 7 D-F source data 2/Fig7 D-F labelled blots.docx]

4-15 blot 1

Blot 2 4-17

Blot 3 4-18
